# Development of a multi-route physiologically based pharmacokinetic (PBPK) model for nanomaterials: a comparison between a traditional versus a new route-specific approach using gold nanoparticles in rats

**DOI:** 10.1186/s12989-022-00489-4

**Published:** 2022-07-08

**Authors:** Wei-Chun Chou, Yi-Hsien Cheng, Jim E. Riviere, Nancy A. Monteiro-Riviere, Wolfgang G. Kreyling, Zhoumeng Lin

**Affiliations:** 1 Department of Environmental and Global Health, College of Public Health and Health Professions, University of Florida, 1225 Center Drive, Gainesville, FL 32610 USA; 2 Center for Environmental and Human Toxicology, University of Florida, Gainesville, FL 32608 USA; 3 Institute of Computational Comparative Medicine, Kansas State University, Manhattan, KS 66506 USA; 4 Nanotechnology Innovation Center of Kansas State, Kansas State University, Manhattan, KS 66506 USA; 5 1Data Consortium, Kansas State University, Olathe, KS 66061 USA; 6 Helmholtz Zentrum München, German Research Center for Environmental Health, Institute of Epidemiology, Ingolstaedter Landstrasse 1, Neuherberg, 85764 Munich, Germany

**Keywords:** Biodistribution, Endocytosis, Gold nanoparticles, Nanomedicine, Nanotoxicology, Physiologically based pharmacokinetic (PBPK) modeling

## Abstract

**Background:**

Physiologically based pharmacokinetic (PBPK) modeling is an important tool in predicting target organ dosimetry and risk assessment of nanoparticles (NPs). The methodology of building a multi-route PBPK model for NPs has not been established, nor systematically evaluated. In this study, we hypothesized that the traditional route-to-route extrapolation approach of PBPK modeling that is typically used for small molecules may not be appropriate for NPs. To test this hypothesis, the objective of this study was to develop a multi-route PBPK model for different sizes (1.4–200 nm) of gold nanoparticles (AuNPs) in adult rats following different routes of administration (i.e., intravenous (IV), oral gavage, intratracheal instillation, and endotracheal inhalation) using two approaches: a traditional route-to-route extrapolation approach for small molecules and a new approach that is based on route-specific data that we propose to be applied generally to NPs.

**Results:**

We found that the PBPK model using this new approach had superior performance than the traditional approach. The final PBPK model was optimized rigorously using a Bayesian hierarchical approach with Markov chain Monte Carlo simulations, and then converted to a web-based interface using R Shiny. In addition, quantitative structure–activity relationships (QSAR) based multivariate linear regressions were established to predict the route-specific key biodistribution parameters (e.g., maximum uptake rate) based on the physicochemical properties of AuNPs (e.g., size, surface area, dose, Zeta potential, and NP numbers). These results showed the size and surface area of AuNPs were the main determinants for endocytic/phagocytic uptake rates regardless of the route of administration, while Zeta potential was an important parameter for the estimation of the exocytic release rates following IV administration.

**Conclusions:**

This study suggests that traditional route-to-route extrapolation approaches for PBPK modeling of small molecules are not applicable to NPs. Therefore, multi-route PBPK models for NPs should be developed using route-specific data. This novel PBPK-based web interface serves as a foundation for extrapolating to other NPs and to humans to facilitate biodistribution estimation, safety, and risk assessment of NPs.

**Supplementary Information:**

The online version contains supplementary material available at 10.1186/s12989-022-00489-4.

## Background

Nanoparticles (NPs) have been widely applied in a number of areas, including as consumer products and carriers for the delivery of drugs [1, 2]. There is a growing interest in applying nanotechnology products to cancer-treatment due to their unique physicochemical characteristics when utilized in drug delivery, diagnosis, imaging, and in some cases the inherent therapeutic properties of some nanomaterials themselves [2]. Despite the significant advancement in the synthesis and design of cancer-targeting NPs, the development of NPs-based drug formulations are still hindered partially due to their low delivery efficiency to the target tissues, such as tumors [3]. Recent meta-analysis studies showed that on average only 0.7% of the injected NPs dose reaches the tumor [4, 5]. This is generally attributed to the poor understanding of the pharmacokinetics and accumulation of the designed NP carrier systems in target organs in vivo and how the physicochemical properties of the designed NPs affect their pharmacokinetic behavior in the biological systems. Animal studies are useful in characterizing the general pharmacokinetics of nanomedicines, but it would be both time- and cost-prohibitive and impractical to perform animal pharmacokinetic studies for every new type of NPs. The increasing use of NPs has increased human exposures through different routes and raises concern about potential adverse effects on human health. In order to design safe and therapeutically effective NPs, and to properly assess potential risks of NPs, there is a pressing need for a quantitative tool that can predict the pharmacokinetics and tissue distribution of NPs based on their physicochemical properties following different routes of exposure. This model will improve our understanding of the various roles of the physicochemical factors on the absorption, cellular uptake, disposition, target organ dosimetry, and elimination of NPs following different routes of exposure to support risk assessment and clinical translation of NPs from their design to treatment strategies.

Physiologically based pharmacokinetic (PBPK) modeling is a mechanistic approach based upon the physicochemical properties of the modeled substance coupled with realistic anatomy and physiology of a living system with organs and tissues interconnected by blood flow to characterize internal organ dosimetry after external exposure. Within the PBPK-simulated living system, kinetic processes of mass transport, such as uneven distribution between tissue and blood, membrane permeability, cellular uptake, and clearance can be included and described mathematically based on NP- and species-specific characteristics. A well-developed PBPK model can integrate available pharmacokinetic and toxicological data and gain in-depth insights into the target tissue dosimetry, potential risk, species differences, and facilitate in vitro to in vivo extrapolations (IVIVE) of NPs [6–10].

Recently, great progress has been made in developing and employing PBPK models to predict internal dosimetry of different NPs (including pharmaceutical NPs) after various routes of exposure [10–12], such as quantum dots [13], silver [14], titanium dioxide [15], polyacrylamide [16], dendrimers [17], nanocrystals [18], and gold [8, 19–21]. Among available PBPK models, most were based upon intravenous (IV) injection-derived pharmacokinetic data in laboratory animals, with only a few incorporating multiple exposure routes [14, 22]. These multi-route models were built using a traditional route-to-route extrapolation approach that is applicable to small molecules, but whether this approach is equally applicable to NPs has not been systematically tested. Unlike small molecules, upon contact with different body fluids following different routes of administration, NPs will be covered with different proteins and other biomolecules, producing different biomolecular coronas and resulting in different patterns of biodistribution [23–31]. Based on this fact, we hypothesize that the traditional route-to-route extrapolation approaches of PBPK models for small molecules may be inappropriate for NPs.

Existing NP PBPK models were built based on data from various studies (i.e., different labs with different experimental designs, measurement methods, and physicochemical characteristics of the NPs), which introduces high uncertainty and variability into the models and often prevents an investigator from integrating available data to gain deeper insights into the role of the physicochemical properties on NPs tissue dosimetry. PBPK models for different routes of administration with different sizes of well-characterized NPs have not been reported, in part, due to lack of consistent and comprehensive pharmacokinetic data. Without such models, the roles of NP physicochemical characteristics, including surface area, number of NPs, Zeta potential, surface functionalization, and dosages on tissue dosimetry as well as cellular endocytosis/phagocytosis are difficult to systemically and quantitatively investigate. Finally, and relevant to the actual adoption and utilization of these approaches in cancer nanomedicine and risk assessment, none of the existing NP PBPK models have been implemented in a web-based interface, which is extremely important to facilitate model applications.

Previously, our group has developed PBPK models of gold nanoparticles (AuNPs) in mice, rats, pigs, and humans following IV administration [8, 19, 32]. These models provide a foundation to extrapolate to other routes of exposure. In recent years, one team member (WGK) has collected pharmacokinetic data for different sizes of AuNPs in adult rats following different routes of administration, including IV [33], oral [34], intratracheal instillation (IT) [35], and endotracheal inhalation (IH) [36]. These data were based on well-characterized AuNPs that were synthesized using the same method and conducted in the same laboratory with consistent experimental protocols. Built upon our earlier PBPK models and based on the data gaps described above as well as the availability of these comprehensive and consistent pharmacokinetic datasets for AuNPs [33–36], the objectives of this study were fourfold: (1) to test our hypothesis and to determine whether the traditional route-to-route extrapolation approaches of PBPK models for small molecules are appropriate for NPs; (2) to develop a user-friendly interactive PBPK web application, Nano-iPBPK (https://pbpk.shinyapps.io/NanoiPBPK/), to predict the biodistribution of AuNPs in rats following different routes of exposure; (3) to determine the role of different physicochemical factors on the absorption, cellular uptake, disposition, target organ dosimetry, and elimination of NPs; and (4) to apply this model to support risk assessment and the design and clinical translation of new NPs.

## Results

### Workflow for developing a user-friendly nano-iPBPK interface to predict the biodistribution of AuNPs

**Fig. 1:**
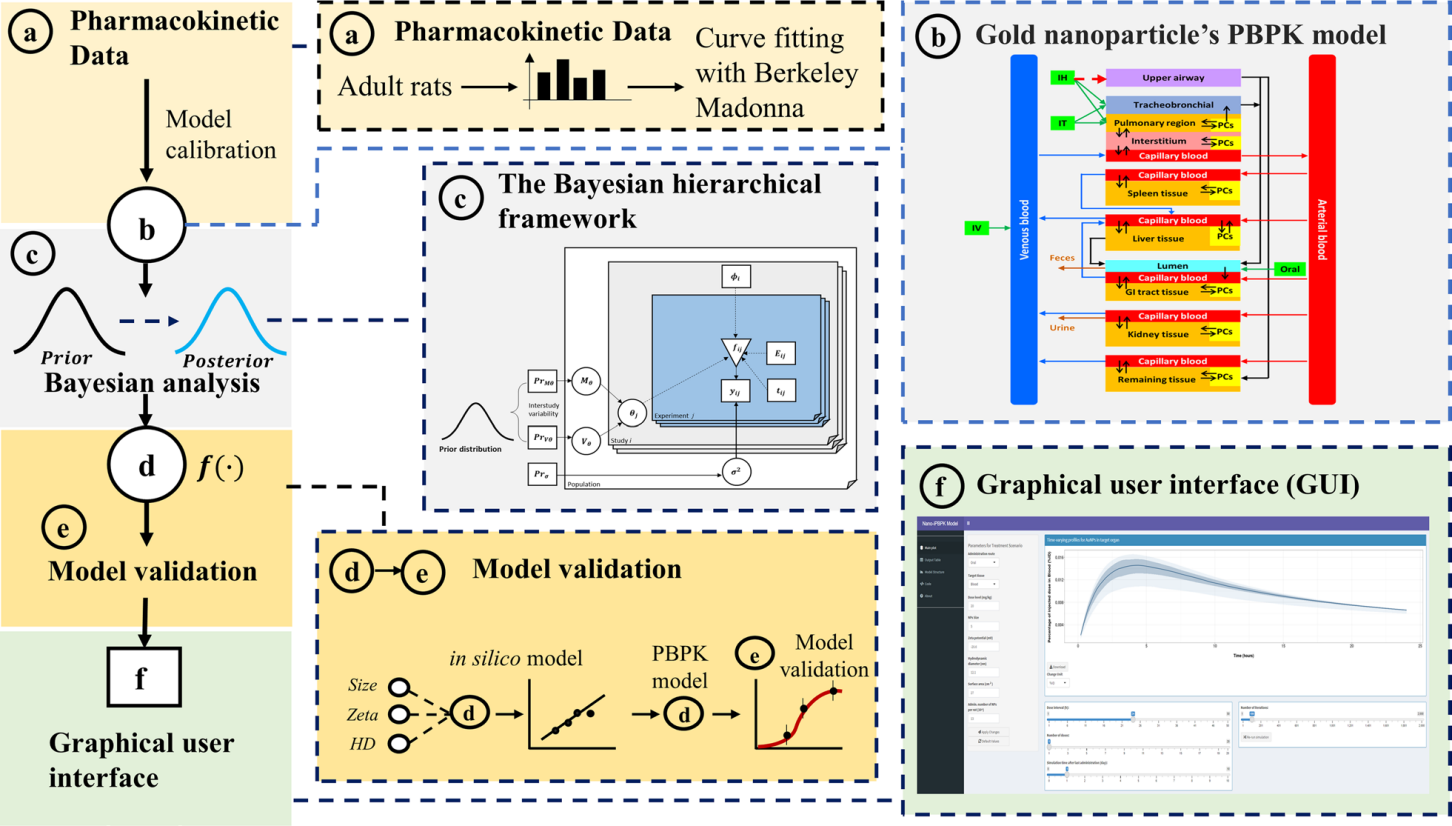
Workflow for the nanoparticle interactive physiologically based pharmacokinetic (Nano-iPBPK) model development. **a** Pharmacokinetic data of gold nanoparticles (AuNPs) in adult female rats were collected. **b** A mechanistic-based PBPK model was calibrated with experimental datasets. **c** The model was optimized and parameter uncertainty and variability were characterized within a Bayesian framework via Markov chain Monte Carlo (MCMC) simulations. **d** A multiple-linear regression-based quantitative structure–activity relation (QSAR) model was developed to determine the relationship between key biodistribution parameters and physicochemical properties of AuNPs. **e** The multi-variate linear regression-based in silico QSAR model was integrated into the PBPK model, and the final model was subjected to evaluation/validation with independent data. **f** The final PBPK model was converted to a web-based graphical user interface termed Nano-iPBPK

The workflow for the development of the Nano-iPBPK application is shown in Fig. 1. In the first step, AuNPs pharmacokinetic data were collected and analyzed in adult female rats following various routes of administration, including IV [33], oral [34], IT [35], and IH [36] (Table 1). Details of these pharmacokinetic studies are provided in Additional file 1: Table S1. With these pharmacokinetic data, a multiple-route NP PBPK model was established based on the recently published model structure [8, 19, 32] and was initially calibrated with the “Curve Fitting Module” in the Berkeley Madonna mathematical modelling software package (shown in Fig. 1a and Fig. 1b). The model structure is provided in Additional file 1: Fig. S1 and model parameters are listed in Additional file 1: Tables S2 and S3. Next, the model was translated to the R program and a Bayesian approach with Markov chain Monte Carlo (MCMC) simulation [37] was then incorporated into the model to optimize model parameters, characterize uncertainty, and improve performance (Fig. 1c). Next, multiple-linear regression equations describing the relationships between physicochemical properties and the key cellular uptake-related organ-specific pharmacokinetic parameters of AuNPs were incorporated into the PBPK model; and then the final model was evaluated with independent/external data following the established guidelines [38] (Fig. 1d, e). This model can be used to simulate the tissue distribution of different types of AuNPs in multiple target organs following different routes of exposure based on the physicochemical properties of AuNPs in rats. To facilitate model application, this final model was converted to a web-based application, Nano-iPBPK (Fig. 1f).

**Table 1:** Summary of pharmacokinetic studies in rats after oral, intravenous (IV), intratracheal instillation (IT) and inhalation (IH) administration for model calibration and evaluation

Exposure route	Size (nm)^a^	Coating	Dose (µg)	Post-exposure sampling times (hours)	Tissues sampled	Strain	References
*Calibration*							
IV	1.4, 5, 18, 80, 200	Citrate	3.2–32.9	1, 24	Bl, GI, Lu, Li, Sp, Ki, Rt, U	Wistar − Kyoto rats	[33]
Oral	5, 18, 80, 200	Citrate	2.6–28.8	1, 24	Bl, GI, Lu, Li, Sp, Ki, Rt, U	Wistar − Kyoto rats	[34]
IT	1.4, 5, 18, 80, 200	Citrate	3.0–40.7	1, 3, 24	Bl, GI, Lu, Li, Sp, Ki, Rt, U	Wistar − Kyoto rats	[35]
IH	23	Citrate	20.1	2, 4, 24, 168, 672	Bl, GI, Lu, Li, Sp, Ki, Rt	Wistar − Kyoto rats	[36]
*Validation*							
IV	22.4, 33.2, 33.7, 35.1, 34.9	Citrate, 11-MUA, CALNN, CALND, CALNS	1	24	Bl, GI, Lu, Li, Sp, Ki, Mu, Br, Bo, Rt	Wistar rats	[47]
IV	16.1	Citrate	0.7	672	Bl, GI, Lu, Li, Sp, Ki, Mu, Br, Bo, Rt	Wistar rats	[48]

*11-MUA* 11-mercaptoundecanoic acid, *CALNN* Cys-Ala-Leu-Asn-Asn, *CALNS* Cys-Ala-Leu-Asn-Ser, *CALND* Cys-Ala-Leu-Asn-Asp, *Bl* blood, *Br* brain, *IV* intravenous, *Ki* kidney, *Lu* lung, *Li* liver, *Mu* muscle, *GI* Gastrointestinal tract, *Rt* remaining tissues, *Sp* spleen, *U* urine

^a^The size represents the core diameter of AuNPs

### Parameter estimation within Bayesian framework

**Fig. 2:**
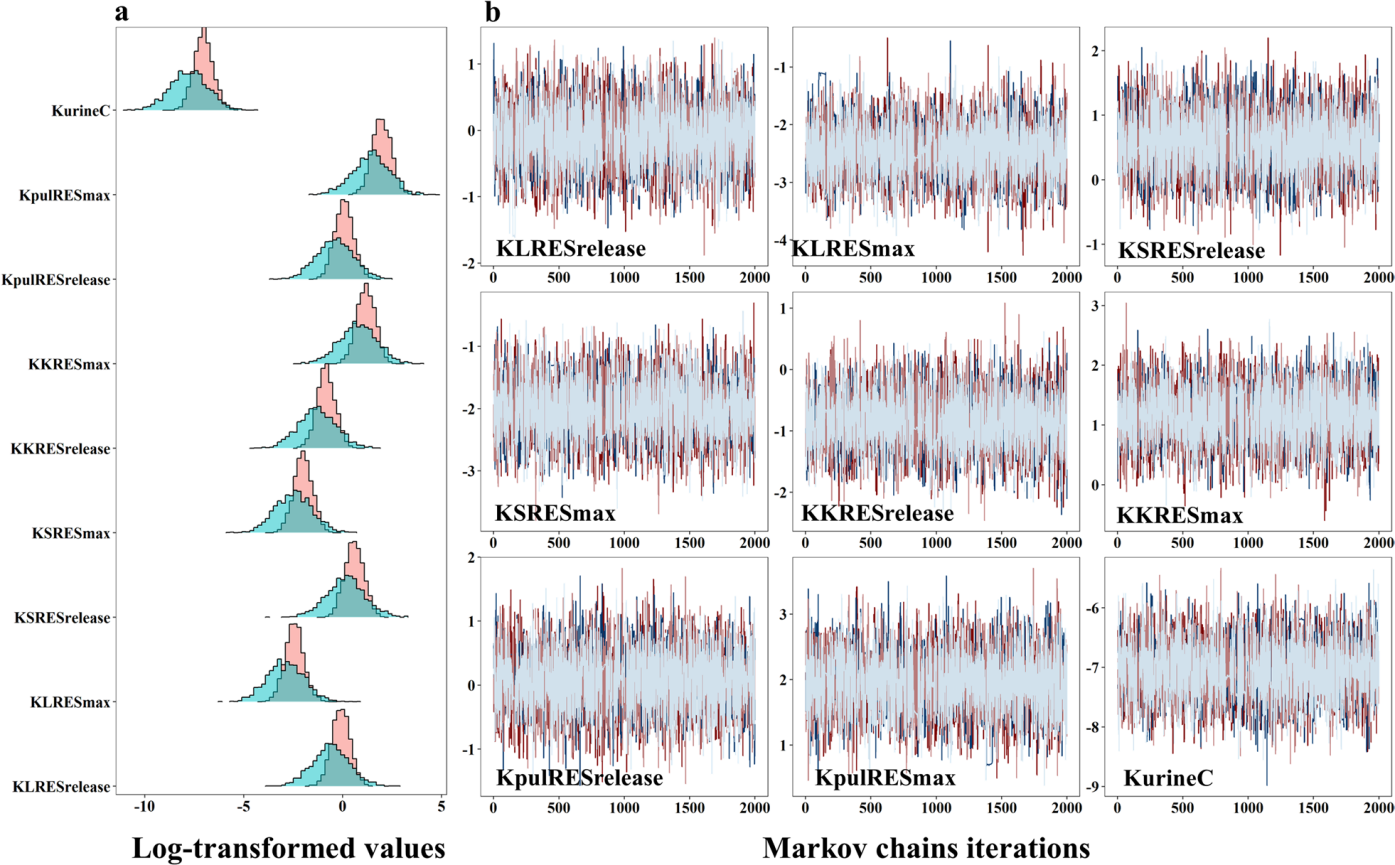
The density and traces plots for posterior parameters. **a** Densities of prior (blue color) and posterior (pink color) parameter uncertainty distributions of log-transformed population means. **b** The Bayesian traces plot of four Markov chains of the last 2000 iterations of the Markov chain Monte Carlo (MCMC) simulations from the PBPK model in rats orally receiving 19.6 μg of 80 nm AuNPs. Gelman and Rubin Shrink Factors: Potential scale reduction factors: $\hat{R}$ = 1.0–1.02

The results from preliminary calibration in Berkeley Madonna are shown in Additional file 1: Figs. S2 for IV, S3 for oral, S4 for IT and S5 for IH exposures. Overall, the initial version of the model was able to adequately simulate the biodistribution of different sizes of AuNPs after different routes of exposure in rats. The parameter values estimated from the preliminary calibration were used as the prior parameters (Additional file 1: Table S3) for the subsequent model optimization via the Bayesian-MCMC analysis. The sensitive model parameters including release rate constants of phagocytic cells (KLRESrelease, KSRESrelease, KKRESrelease and KpulRESrelease), maximum uptake rate constants (KLRESmax, KSRESmax, KKRESmax and KpulRESmax) and elimination rate constant (KurineC) were included in the Bayesian-MCMC analysis (these parameters are further defined in Additional file 1: Table S4). Figure 2 depicts a representative figure for the densities of prior and posterior uncertainty distributions and traces plots for these parameters from the PBPK model in rats receiving the 80 nm AuNPs orally. The results showed that all posterior uncertainty distributions were narrower than prior distributions, indicating the prior parameters had been updated by experimental data and then turned into the more stable posterior parameters (Fig. 2a). Figure 2b depicts the convergences of four Markov chains for several representative parameters in the oral PBPK model. The well-mixed traces plots with a value of ${ }{\hat{\mathbf{R}}}$ < 1.2 for all chains suggested that convergences were achieved (Fig. 2b). In summary, the statistics of the posterior distributions of population geometric mean, population geometric standard deviation of model parameters for the PBPK model with different routes of administration for different sizes of AuNPs is provided in Additional file 1: Tables S4 for oral, S5 for IV, S6 for IT, and S7 for IH exposures.

### Global evaluation of goodness of model fit

**Fig. 3:**
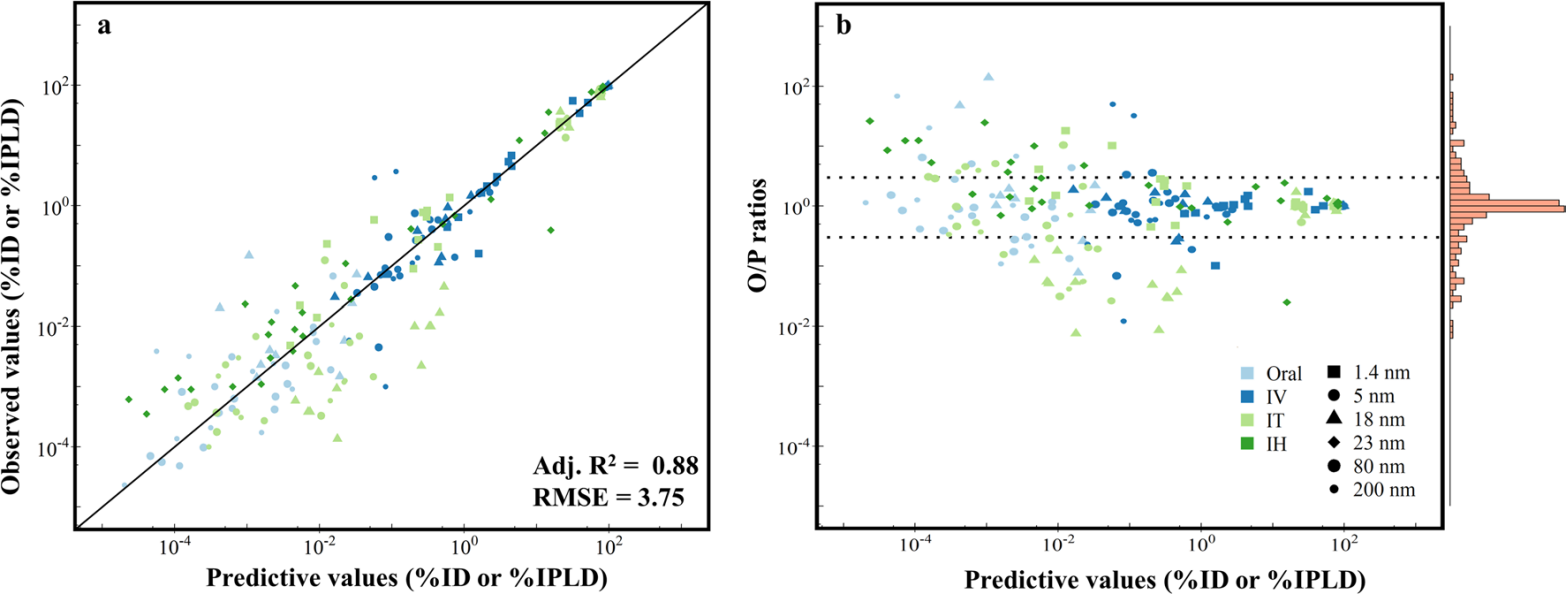
Comparisons of model prediction (x-axis) with observed data (y-axis). **a** Global evaluation of goodness of model fit from the PBPK model calibration results for pharmacokinetic data from rats after oral administration (Oral), intravenous injection (IV), intratracheal instillation (IT) and inhalation administration (IH) of AuNPs. **b** Observed-to-predicted (O/P) ratio versus model prediction plot. In both panels, the different symbol shapes and colors are used for different NPs’ sizes (1.4, 5, 18, 23, 80 and 200 nm) and exposure routes (Oral, IV, IT and IH), respectively. In panel b, the dashed line represents over a O/P ratio of 2 or lower 0.5, and the histogram of residuals is shown on the right of the panel

The goodness of model fit was evaluated by comparing model-predicted median concentrations or amounts with measured mean values (given in percent of the initial dose [%ID] or percent of the initial peripheral lung dose [%IPLD]) in selected tissues and organs of rats after different routes of administration for different sizes of AuNPs (Fig. 3a). Overall, the model simulations were in good agreement with the observed data [Adjusted *R*-square value was 0.88 and the root mean square error (RMSE) was 3.75]. Additionally, most predictions were within 2- or threefold difference of the observed data. Specifically, 58% and 67% of the predictions were within < twofold and < threefold error, respectively, suggesting adequate model predictions (Fig. 3b). However, the visualized agreements between model predictions and observed values (Fig. 3a) were more scattering at the area of low observed values (10^–4^–10^–2^ %ID) than at the larger observed values (10^–2^–10^2^ %ID), indicating that the model may not predict well at the low dose or concentration range, possibly due to the detection limit or the higher inter-individual variability in some of the collected data.

### Comparisons of measured vs. model-predicted time-course kinetic profiles

**Fig. 4:**
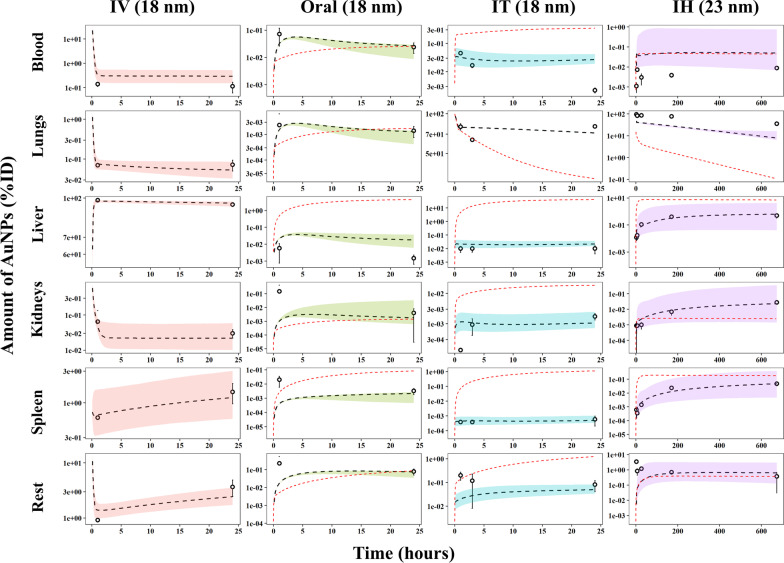
The probabilistic time-course predictions [median (black-dashed line) and 95% confidence interval (CI) (color shadows)] from the nano-iPBPK model calibration results compared with pharmacokinetic data in rats after intravenous injection (IV), oral administration (Oral), intratracheal instillation (IT) of 18 nm AuNPs and endotracheally inhaled (IH) exposure of 23 nm AuNPs. Symbols and error bars (mean ± SD) represent measured amounts and dashed lines represent simulated amounts of AuNPs (percent of the initial dose [%ID] or initial peripheral lung dose [%IPLD]) in blood, lungs, liver, kidneys, spleen, remaining tissues. The read-dashed lines represent the simulation results from the PBPK model derived using the traditional route-to-route extrapolation approach

To visually confirm predictability, the probabilistic time-course predictions [median and 95% confidence interval (CI)] based on the posterior parameters after Bayesian-MCMC analysis were generated and compared with measured amounts of Au in selected tissues and organs of rats after different routes of administration for different sizes of AuNPs. The final PBPK model adequately predicted the biodistribution of AuNPs after IV (18 nm), oral (18 nm), IT (18 nm) and IH (23 nm) administration in most tissues except slightly underestimating at the earlier time point in kidney, spleen and rest of body after oral exposure to 18 nm AuNPs and underestimating in lungs and overestimating in blood for 23 nm AuNPs after IH administration (Fig. 4). Figure 4 presents representative results from a subset of particles based on the selection criterion of similar particle sizes of nearly 20 nm (i.e., 18–23 nm) from each of the administration routes. Results on other sizes of AuNPs following different routes of exposure are provided in the Additional file 1: Figs. S6 for IV, S7 for oral, and S8 for IT exposures.

### Comparisons of the traditional route-to-route extrapolation approach for small molecules vs. a new route-specific approach for NPs

The present multi-route model was calibrated using route-specific pharmacokinetic data, which is the proposed new approach for NPs in this study. In contrast, the traditional route-to-route extrapolation of PBPK models for small molecules was performed by keeping chemical-specific parameters from one administration route (i.e., typically the IV route) the same for the other routes (e.g., oral, IT, and IH). To assess whether the traditional route-to-route extrapolation approach is applicable for AuNPs, particle-specific parameters such as endocytosis-related parameters, distribution coefficients and elimination rate constants that were optimized following IV administration were used directly to simulate the pharmacokinetics of AuNPs following oral, IT, and IH exposure. The comparisons between the simulation results using the traditional approach versus the new route-specific approach used in this current study for AuNPs are presented in Fig. 4. The model simulation results using the traditional approach either overestimated or underestimated the measured data for AuNPs following oral of 18 nm, IT of 18 nm, and IH of 23 nm exposure doses (red dashed lines in Fig. 4). Results from the final model were optimized using the administration route-specific pharmacokinetic data which appeared better than the traditional model extrapolations.

### Sensitivity analysis for route- and tissue-specific biodistribution parameters

**Fig. 5:**
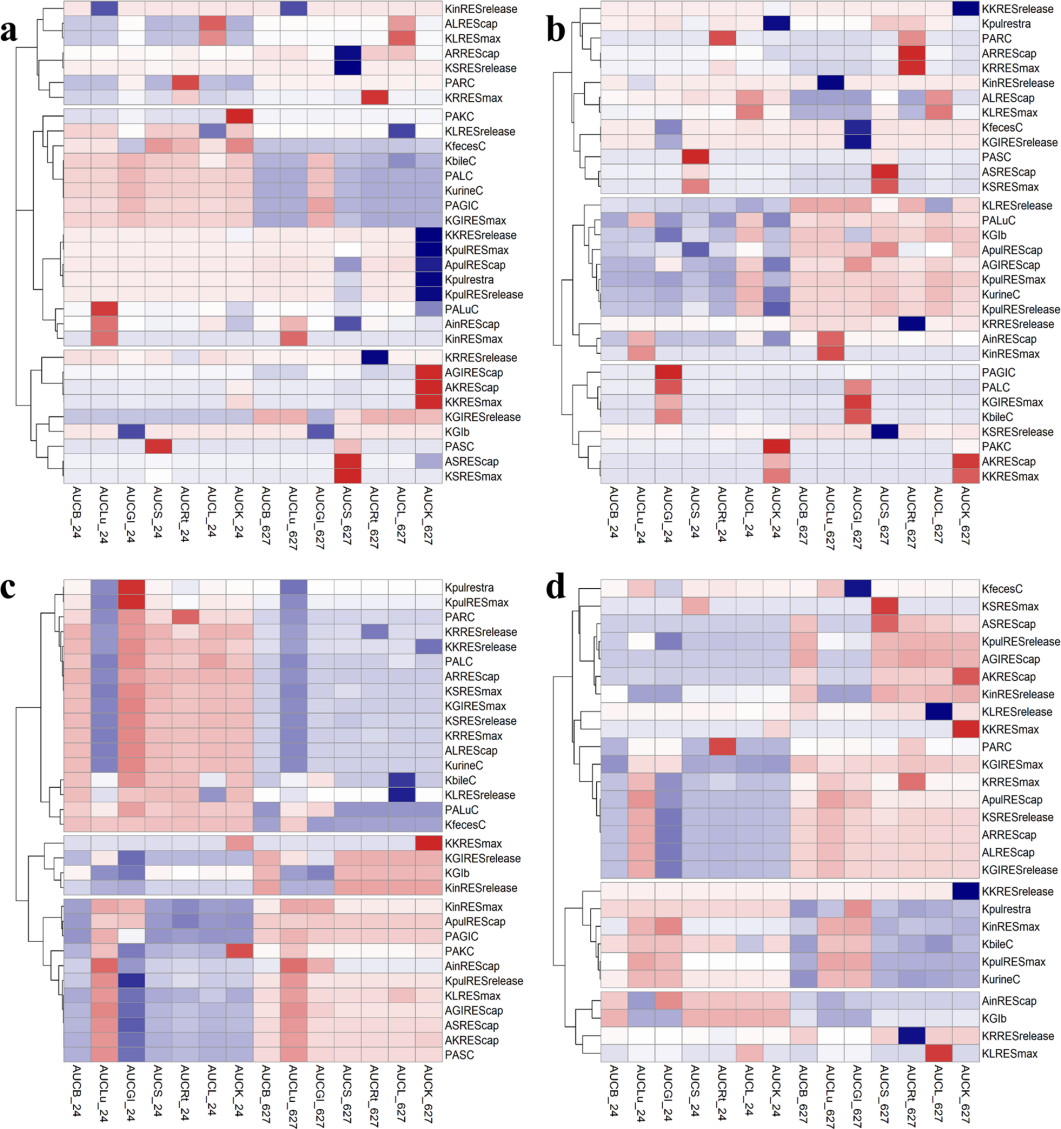
Heat map plot of the normalized sensitivity coefficient (NSC) for comparative sensitivity analyses for 5 nm AuNPs at 24 h and 672 h following: **a** oral administration (Oral), **b** intravenous injection (IV), **c** intratracheal instillation (IT), and **d** 23 nm AuNPs following inhalation administration (IH). The plot identifies highly influential parameters with darker colors (red or blue) to the 24 h and 672 h dose metrics. Values of NSCs are provided in Additional file 1: Tables S8–S11. AUCB, AUCLu, AUCGI, AUCS, AUCL, AUCK and AUCRt represent area-under-the-concentration curve of gold nanoparticles (AuNPs) in blood, lungs, gastrointestinal (GI) tract, spleen, liver, and kidneys and remaining tissues, respectively

To determine which parameters (i.e., physiological and AuNP-specific parameters) play important roles in governing the route- and time-specific dose metrics in rats, local sensitivity analyses were conducted. Specifically, positive values of normalized sensitivity coefficient (NSC) estimates indicate that an increase in the parameter value would increase the dose metrics (i.e., area-under-the-concentration curve, AUC). Figure 5 displays the heat map plot for the NSC values of 5 nm AuNPs at short-term (24 h) and long-term (672 h) AUCs following oral (Fig. 5a), IV (Fig. 5b), and IT (Fig. 5c), as well as 23 nm AuNPs after IH (Fig. 5d) administration. The heat map plot for NSC values of 18, 80 and 200 nm AuNPs at 24 h and 672 h AUC following oral (A, D, G), IV (B, E, H) and IT (C, F, I) are displayed in Additional file 1: Fig. S9. The specific NSC values after different routes of exposure are provided in Additional file 1: Tables S8 for oral, S9 for IV, S10 for IT, and S11 for IH exposures. Our results indicate that the set of sensitive parameters were similar with the same administration route across different particle sizes, but the sensitive parameters were varied with different administration routes (Fig. 5 and Additional file 1: Fig. S9). The NSC results for 5 nm AuNPs following oral, IV, and IT administrations and for 23 nm following IH exposure were used as representative results and explained further below.

For oral administration with 5 nm AuNPs (Fig. 5a and Additional file 1: Table S8), an increase in parameter values, including maximum uptake rate (KLRESmax, KKRESmax) and release rate constant (KLRESrelease, KKRESrelease) of phagocytotic cells in liver and kidney, fecal excretion rate constant (KfecesC) and urinary clearance constant (KurineC), contributed significantly to the overall predicted AuNP kinetics both positively and negatively (Fig. 5a and Additional file 1: Table S8). However, change in endocytic/phagocytic uptake capacity had negligible impact on the AuNP distribution after oral administration. Similar to the oral exposure, maximum endocytic/phagocytic uptake rate (KLRESmax) and the release rate constant of phagocytic cells in the liver (KLRESrelease) were identified to have significant impacts on the overall AUC estimates after IV administration (Fig. 5b and Additional file 1: Table S9). The endocytic/phagocytic uptake capacity in the liver also showed greater impact (NSC ≥ 0.5) to both short-term and long-term AUCs after IV administration. For IT administration, the changes in phagocytic uptake capacity in the lung (APulREScap) had a relatively substantial impact (NSC ≥ 0.5) on the short-term dose for blood, lung, liver, spleen, kidneys, and remaining tissues for 5 nm of AuNPs (Fig. 5c and Additional file 1: Table S10). The uptake rates of phagocytic cells in liver, kidney, GI tract and spleen also had a significant impact on the short-term dose.

Inhalation-associated NSC estimates revealed that a 1% change in maximum endocytic uptake rate and release rate in lungs (KPulRESmax and KPulRESrelease) had significant impacts on both short-term (i.e., 24 h) and long-term (i.e., 672 h) dose (Fig. 5d and Additional file 1: Table S11); whereas the parameters of endocytic uptake capacity in lungs, liver and remaining tissues mainly affected the short-term dose. In addition, the other parameters, including urinary and fecal elimination rate constants also had low-to-high contribution to the short-term and long-term AUCs.

### Quantitative structure–activity relationship (QSAR) models with multiple linear regression equations to predict route-specific key biodistribution parameters based on the physicochemical properties of AuNPs

To enable predictions of route-specific key biodistribution parameters based on the physicochemical properties that are usually readily available when new NPs are synthesized and characterized, QSAR models based on multivariate linear regression analyses were established using stepwise selection and statistical criteria [4]. Table 2 shows the multivariate linear regression results (i.e., slope of selected variables (*β*_*i*_)) and statistical criteria for IV administration, and the results for oral and IT administrations are shown in Additional file 1: Table S12 for oral exposure and Table S13 for IT exposure. Note that multivariate linear regression models of three or more variables were unable (large *p* values of > 0.05) to describe the relationships between AuNP characteristics and estimated distribution parameters. Not all multivariate relationships between AuNP characteristics and tissue distribution parameters were statistically significant (*p* < 0.05) for individual tissues, some apparent associations of the maximum uptake rate (*K*_*max*_) and uptake capacity (*A*_*cap*_) with properties such as hydrodynamic diameter (HD), surface area (SA), and number of NPs [log(NPs)] were found after different routes of administration (Table 2, Additional file 1: Tables S12 and S13). For excretion following oral and IT administrations, only HD and SA were significant determinants for the prediction of urinary, fecal and biliary excretion rate constants (Additional file 1: Tables S12 and S13).

**Table 2:** Final multivariate linear regression models describing relationships between the physicochemical properties of AuNPs and biodistribution parameters following intravenous injection in rats

Tissue/Organ	*β*_0_	*β*_1_	*β*_2_	*β*_3_	*β*_4_	*β*_5_	*β*_6_	*β*_7_	*BIC*	Adj-*R*^2^	*p*-value
Intravenous injection (IV)											
Maximum endocytosis/phagocytosis uptake rate											
Lung	3.77	0.01						− 0.11	5.54	0.93	0.03
GI tract	48.2	− 0.08						− 1.47	15.8	0.99	0.02
Liver	317		4.69					− 10.7	41.8	0.91	0.04
Spleen	− 1.23E4			178			5.28E3		50.9	0.80	0.09
Kidney	2.02		− 0.49			3.59			11.0	0.93	0.03
Rest of body	0.067		0.12			− 0.38			5.21	0.83	0.08
Endocytosis/phagocytosis uptake capacity											
Lung	− 448		3.01			44.7			35.8	0.97	0.01
GI tract	29.4		− 3.04			46.6			34.7	0.98	0.01
Liver	8.21E3					1.08E3		− 251	69.7	0.95	0.03
Spleen	− 76.1		131					− 151	74.7	0.91	0.05
Kidney	228		− 64			467			57.2	0.96	0.02
Rest of body	− 126					12.2		6.01	36.7	0.98	0.01
Release rate constant of phagocytic cells											
Lung	− 4.17			0.058			1.78		− 24.3	0.48	0.26
GI tract	− 1.17		0.015	− 0.052					− 23.5	0.99	0.001
Liver	0.07	− 3E−4				− 0.029			− 15.4	0.07	0.49
Spleen	− 0.22		0.004	− 0.009					− 56.5	0.99	3.64E-5
Kidney	4.56	− 0.03				1.56			26.8	0.14	0.43
Rest of body	− 0.11	3E−3	0.031						− 11.8	0.96	0.018
Urinary and fecal excretion constant rate											
Urine	3.5E−4		− 1E−4			7.4E−4			− 76.4	0.96	0.019
Feces	− 1.36	0.002						0.054	− 18.1	0.97	0.015
Biliary	0.03	1.4E−4		0.001					− 58.6	0.98	0.001

*β*_0_ and *β*_*i*_ represent intercept and slope of variables included in the multivariate linear regression model, respectively [1: hydrodynamic diameter (HD); 2: surface area (SA); 3: Zeta potential (Z); 4: log(HD); 5: log(SA); 6: log(Z); 7: log-transformed number of NPs, log(NPs)]. The values in bold represent statistical significance of *p* < 0.05

*BIC* Bayesian information criterion, *Adj-R*^*2*^ adjusted *R*^2^

### Model evaluation/validation with independent data

**Fig. 6:**
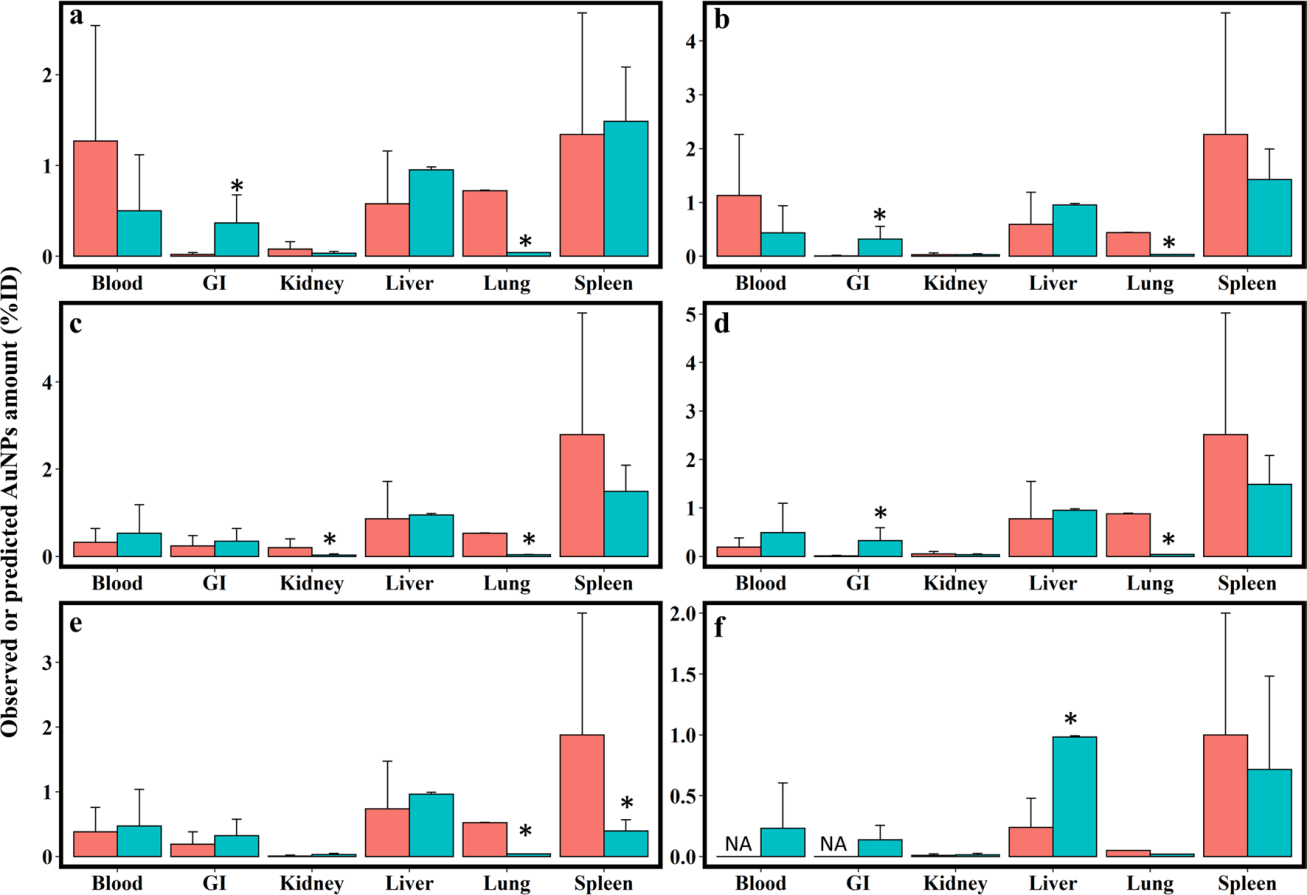
Model evaluation results with pharmacokinetic data in rats following intravenous injection (IV) of AuNPs [47, 48] with different surface coatings, including: **a** citrate (the same coating as our calibration data), **b** 11-mercaptoundecanoic acid (11-MUA), **c** Cys-Ala-Leu-Asn-Asn (CALNN), **d** Cys-Ala-Leu-Asn-Asp (CALND), **e** Cys-Ala-Leu-Asn-Ser (CALNS) and **f** citrate. The red and light green bars represent the observed and predicted values, respectively. The pharmacokinetic data of **a**–**e** and **f** were collected at 24 h and 28 days after administration of AuNPs from Morais et al. [47] and Fraga et al. [48], respectively. The physicochemical characteristics of AuNPs are summarized in Table S14 in the Additional file 1. *Indicates that the difference between the simulated mean concentration and the observed mean concentration was more than twofold

To further evaluate Nano-iPBPK’s predictability, this model was evaluated with several independent pharmacokinetic datasets (i.e., not used in the model calibration) for various characteristics of AuNPs (Additional file 1: Table S14). The model simulations were compared against measured data for adult rats exposed to AuNPs with different surface coatings (Cit-AuNPs, MUA-AuNPs, CALNN-AuNPs, CALND-AuNPs and CALNS-AuNPs) (abbreviations defined in Table 1 footnote), sizes (from 22.4 to 34.9 nm) and Zeta potentials (from − 47.1 to − 37.3 mV) through IV administrations in different tissues (blood, GI, kidney, liver, lung and spleen) (Fig. 6 and Additional file 1: Table S15). The model was evaluated with short-term (24 h) (Figs. 6a–e) and long-term (28 days) (Fig. 6f) kinetic data. These results showed that the simulated results properly captured the data points for AuNPs in these pharmacokinetic datasets for blood and most tissues (the ratio between predicted and observed values were generally within the twofold error range), except for the lungs and GI tract. The model-predicted %ID of AuNPs underestimated in the lungs across all types of AuNPs, while model overpredicted the concentrations in GI tracts for the NPs with surface coatings of citrate, 11-MUACALND and CALNS (Fig. 6 and Additional file 1: Table S15).

### Overview of the nano-iPBPK and its applications

**Fig. 7:**
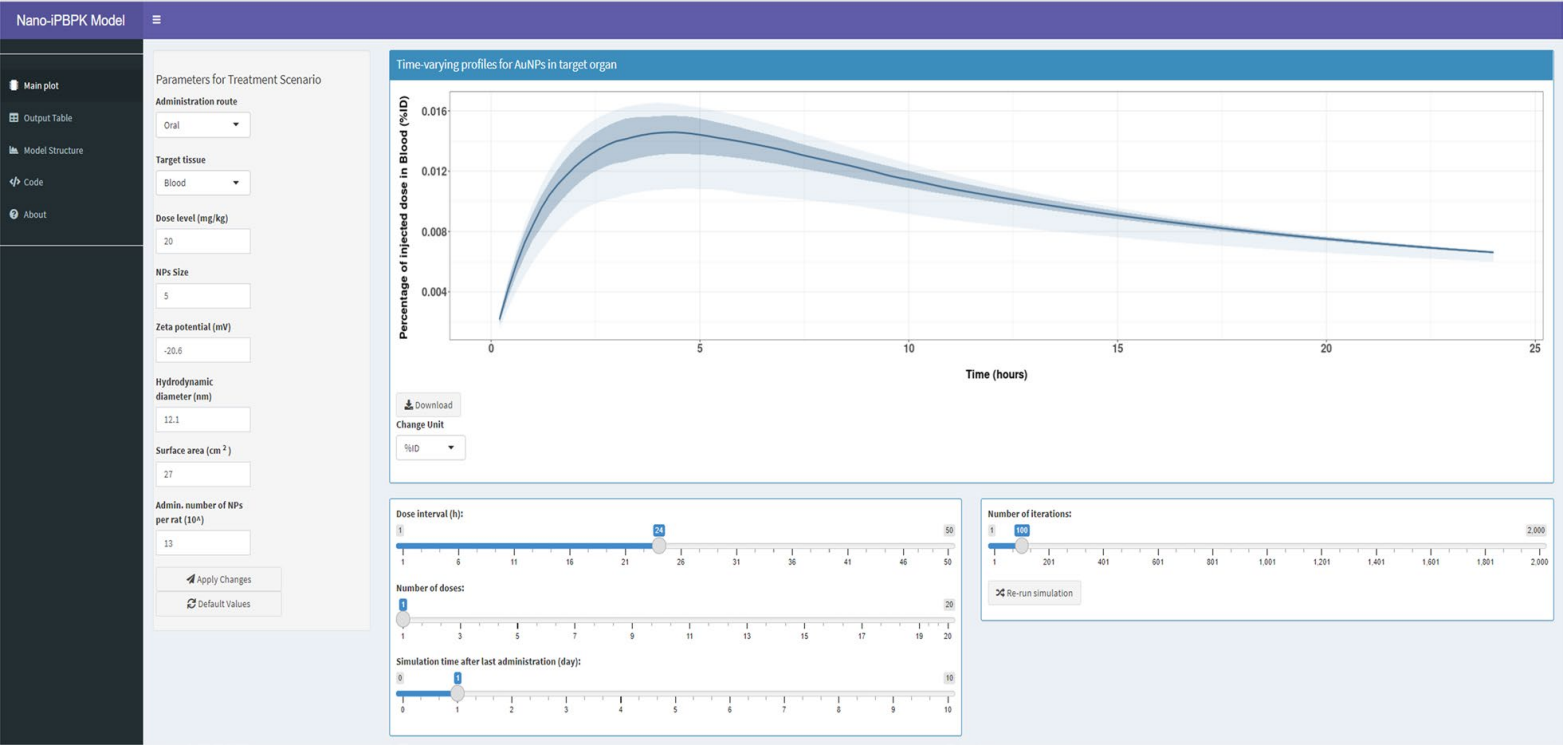
A screenshot of the developed web-based nanoparticle interactive physiologically based pharmacokinetic (Nano-iPBPK) interface for gold nanoparticles (AuNPs) for adult rats

By integrating the above-mentioned multi-route PBPK model and the in silico multivariate regression-based QSAR model applied in the prediction of biodistribution parameters for AuNPs, the final Nano-iPBPK was implemented with the R Shiny framework (https://pbpk.shinyapps.io/NanoiPBPK/). This enables the final model to leverage the computational power of R program and the user-friendliness and web interactivity of R Shiny. Nano-iPBPK provides a prediction platform for simulating biodistribution of different types of AuNPs with different physicochemical properties in different target organs following multiple exposure scenarios. A screenshot of the Nano-iPBPK interface is shown in Fig. 7. Applying this web application, once a new NP is synthesized and characterized, researchers can use it to simulate pharmacokinetics and biodistribution after different routes of administration by inputting the physicochemical and dosing parameter values. Users can view the simulation results online and download the results in a TIFF image format or Excel file format. These results will assist researchers to select the most promising type of NPs to progress to animal experimentation.

## Discussion

In the field of PBPK modeling, traditional route-to-route extrapolation for small molecules is typically performed by using administration route-specific parameters and keeping other chemical-specific parameters the same. However, such approaches might not be appropriate for the NPs because NPs and small molecules are very different in their physicochemical properties and mechanisms underlying their pharmacokinetic profiles. One main difference is that upon contact with body fluids, NPs can be immediately covered by proteins and other biomolecules, forming a biomolecular corona, which determines the biological identity and fate of NPs [23–31]. Therefore, following different routes of administration, NPs could have different pharmacokinetic patterns because they will be covered with different molecules, generating different biomolecular coronas. This may be especially relevant since the biomolecular milieu of the respiratory and digestive tract are very different from each other and from the IV scenario where protein corona formation may dominate. Thus, there are large uncertainties if multi-route NP PBPK models are developed based on the traditional route-to-route extrapolation for small molecules. This knowledge gap is important and should be addressed before the prediction of target tissue dosimetry following different routes of exposure. This study demonstrated that the simulation results of the final multi-route PBPK model that was calibrated using route-specific data were more accurate than the simulation results obtained from the model that was extrapolated using the traditional method. These results suggest that when developing multi-route PBPK models for NPs, the traditional route-to-route extrapolation approach for small molecules may not be appropriate. Multi-route PBPK models for NPs should be developed using route-specific data. Our results provide a rational approach for conducting route-to-route extrapolation specifically for AuNPs and this approach is likely applicable to other metal and metal oxide NPs or even organic NPs, although this requires further experimental and PBPK modeling studies to verify. Overall, this study improves our fundamental understanding in the methodology of PBPK modeling for NPs.

This study constructed a QSAR model with multiple linear regression equations to predict the biodistribution parameters as well as to estimate the potential correlations between the physicochemical characteristics of AuNPs and their corresponding PBPK parameters. The regression analysis results suggest that, in general, the HD, SA and number of NPs were found to play a significant role associated with maximum uptake rate and uptake capacity of phagocytic cells despite the different routes of administration (i.e., IV, oral, and IT). For the clearance/excretion of NPs, this study indicated that the HD and SA were associated with the urinary, fecal and biliary excretion rate constants following oral and IT administrations. Several studies reported that the physical and chemical properties of NPs, size, and surface charge, are critical factors that determine their pharmacokinetics and biodistribution [10, 39, 40]. Surface functionalization, such as Zeta potential, was found to associate with the type and scale of biological molecules absorbed that lead to the formation of protein coronas, which in turn had an impact on the uptake of phagocytic cells and their pharmacokinetic behaviors [41–44]. NP size, strongly correlated with renal clearance/excretion due to the pore size limitation of the glomerular filtration in the kidney [45, 46]. The above-mentioned results from previous references are consistent with and support our findings.

In our study, the multivariate regression analysis also showed that the HD was associated with PBPK model parameters describing the endocytosis process, but the association was unseen with Zeta potential. This result might be because AuNPs in our datasets had similar Zeta potentials (Additional file 1: Table S1), thus leading to Zeta potential as an insignificant predictor variable in the model. The multivariate regression model, however, only reflects the association of AuNP properties with endocytosis parameters, it does not rule out of the possibility of Zeta potential having influence on the biokinetics of AuNPs. On the other hand, our analysis showed that the surface area was also significantly associated with the model parameters describing endocytosis and urinary excretion. In addition, we found different combinations of surface properties and biodistribution parameters of AuNPs following different routes of administration. These results indirectly supported our rationale that NPs have different pharmacokinetic behaviors following different exposure routes, potentially due to different biomolecular coronas or differences in the absorption environment affecting particle charge or state and extent of aggregation. Overall, we suggest that the present integrative framework of PBPK and QSAR model can serve as a new approach for predictive simulations of pharmacokinetics and tissue distribution of AuNPs despite differences in the kinetic behaviors and physicochemical properties following diverse routes of administrations. This research can be extrapolated to other types of NPs and to tumor-bearing animals to support the assessment of potential nanotoxicity and the design of new nanomedicines.

Sensitivity analysis results show that the sensitive parameters were similar across different sizes with the same administration route, but they were varied with different administration routes for the same or similar particle size (Fig. 5 and Additional file 1: Fig. S9). The parameter values were also different between different administration routes, even for the similar size of AuNPs (e.g., 18 nm IT vs. 23 nm IH exposure) (Additional file 1: Tables S3–S7). For example, the maximum uptake rate and release rate constant in kidney and urinary and fecal excretion rate constant had a high impact on the model output for oral administration, but they were not sensitive after IV administration. The phagocytic uptake capacity constant in the lung had significant impacts on the overall AUC estimated after IT and IH administration, but the uptake rates of phagocytic cells in liver and kidney were only sensitive in the AUC estimate after IT administration compared with the predictions after IH administration. Despite there were common sensitive parameters between administrations routes, the sensitive parameters contributed differentially to the biokinetics of AuNPs. These results indirectly support our hypothesis that traditional route-to-route extrapolation method may not be applicable to NPs, in part, because the critical kinetic parameters are varied with the administration routes.

In order to demonstrate the predictive validity of the Nano-iPBPK model, the model was evaluated with multiple independent datasets [47, 48] from studies that were not used in the model calibration, albeit with different surface functionalizations. Despite the differences in NP types, the model was able to simulate the measured concentration data of different types of AuNPs from independent datasets in plasma and multiple organs, except for the lungs and GI tracts (Fig. 6). The reason for the misestimation of the accumulated dose in lungs and GI tract is unknown, but it might be due to some organ-specific cellular uptake and transport mechanisms of NPs in the lungs, or the presence of surfactants, that have not been accounted for in the model [49]. These results suggest that additional in vitro cellular uptake studies in major phagocytic cell types in major reticuloendothelial systems (e.g., liver, spleen, and lungs) are needed in the future to improve our understanding of the cellular uptake and transport mechanisms of NPs [50]. Also, the validation datasets did not have data on the HD of AuNPs, which plays an important role in the overall tissue distribution of AuNPs. In addition, our model did not account for biokinetic changes of AuNPs with different surface coatings because only citrate-coated AuNPs data were considered in the model calibration. Thus, the predictions might be uncertain in the validation data if the kinetic profiles were observed based on the AuNPs coated with other surface coatings (Fig. 6a and f were based on citrate-coated AuNPs, whereas Fig. 6b–e were based on AuNPs with other surface coatings). On the other hand, our model might fail at predicting low %ID values because the visualized agreement between predictions and observed values was scattered at the area of low %ID (Fig. 3a). In some observed data, the measured %ID values in lungs or GI tract were very low (< 0.1 %ID), especially in the dataset following IV administration (Fig. 3b). This might be lower than the detection limit and had high variability, which could be difficult to be predicted and thus leading to the inaccuracy of model predictions. Similarly, the model also predicted a low accuracy at the low %ID values in the validation set (Additional file 1: Fig. S10).

Our PBPK model platform can be used to predict the biodistribution of NPs with different size and properties, but there are still several limitations. Firstly, only one type of NPs (i.e., AuNPs) and species (i.e., rats) were considered in our PBPK model platform, thus the model remains to be validated with additional experimental studies in various types of NPs and in different species. Second, the present model was trained with data of AuNPs from a size range of 1.4–200 nm (except IH exposure that only had one size of 23 nm). The model should be able to generate reasonable predictions in the concentration or amount of AuNPs in different organs if the size is within this applicable domain following oral, IT, or IV exposure, but additional validation with more data will help improve the confidence of the model predictions. However, if a user wants to generate simulation results for AuNPs with a size that is outside this applicable domain, then the results may be uncertain and require additional experimental studies to verify. Third, the validation of the present PBPK model was only based on the datasets limited to 16–34 nm of AuNPs. Additional validation analyses with datasets on other sizes of AuNPs will further improve this model. Fourth, our study is difficult to estimate the dose-dependent changes on biokinetics of AuNPs despite the dose effect has been described as having the ability to influence the translocation of NPs from human and mouse lung cells to blood [20]. Bachler et al. [20] has reported that the translocation fraction in alveolar cellular monolayers (human A549 cells) had a significant decrease of the AuNP dose above a dose of 100 ng/cm^2^. However, the datasets used in this study aimed to observe the difference of biokinetics of AuNPs across different routes of administration. Only one dose but different sizes based on different administrated routes was applied in our study, thus it is difficult to estimate the influences of dose effects on biokinetics of AuNPs.

Fifth, it is possible that NPs may agglomerate or aggregate and alter the size and pharmacokinetic properties. This possibility is low as our pharmacokinetic studies always aimed to prevent any significant agglomeration or aggregation of the NPs [33–36]. Nevertheless, additional studies are still needed to consider the impact of possible agglomeration or aggregation in the PBPK modeling of NPs. Sixth, in our iPBPK interface, we provide the option to input the particle size, number, and surface area separately. However, for spherical particles with a specific surface roughness, the particle size, number, surface area, and mass/volume are dependent on each other, and it is possible to calculate surface area based on the particle size and other parameters [51]. Parameter reduction could be considered in future studies to further improve this model if it is only for spherical AuNPs. However, the present model includes the option to input various physicochemical properties, so that the model is generic and can be extrapolated to other types of NPs that are not spherical. Seventh, the present model is in healthy rats, and thus cannot be used to predict delivery efficiency of AuNPs to tumor. However, the present model provides a basis to be extrapolated to tumor-bearing animals by adding a tumor compartment to help address low tumor delivery efficiency issue [4, 52]. Finally, the model does not include the lymphatic system due to the lack of pharmacokinetic data of AuNPs in lymph nodes. Theoretically, lymph nodes can take up NPs and delay the entry of NPs to the bloodstream via the vena cava [53–55]. Simulations suggest that early interactions with the reticuloendothelial system could modulate subsequent systemic distributions [56]. That may induce a delayed effect in which the NPs are distributed to the blood and organs and may cause some uncertainty in the model simulation. However, the present general workflow for the development of the multi-route Nano-iPBPK platform can serve as a proof-of-concept and lay the foundation for extrapolating to other types of NPs and to other species.

## Conclusions

In conclusion, we developed a web-based Nano-iPBPK application for simulating the biodistribution of AuNPs after different routes of administration in adult rats following a comprehensive study workflow encompassing pharmacokinetic data collection to NP PBPK development and optimization. We developed this multi-route PBPK model using two different approaches: a traditional route-to-route extrapolation approach that is applicable for small molecules and a new route-specific approach that we proposed for NPs. Our results suggest that the traditional approach for small molecules is not applicable to NPs, and multi-route PBPK models for NPs should be developed using route-specific data. Bayesian analysis with MCMC simulations was incorporated into the framework so that the final model parameters were rigorously optimized and that the model can be used to characterize the inter-study and inter-individual variability.

This multi-route PBPK model was rigorously calibrated for spherical (non-agglomerated) AuNPs of 1.4, 5, 18, 80, and 200 nm and validated with independent data for AuNPs of 16–35 nm (not convincing for all organs). The model predictions at the low dose/concentration range were not quite confident. A factor of 2–3 or even 10 of deviation is possible at the low dose levels in a specific organ (< 0.01 %ID). The applicable size domain of the model was 1.4–200 nm. Whether the model can be extrapolated to reliably predict tissue distribution of AuNPs with sizes that are outside this range remain to be tested in the future. Also, the model can only be used to predict concentrations or amounts of AuNPs in an organ at the unit of mass and mass per organ volume, respectively. The model still cannot predict the concentration or amount of AuNPs in the unit of number, surface area, or particle volume, which warrants future studies.

The final PBPK model was converted into web-based Nano-iPBPK interface. The Nano-iPBPK application enables users from different disciplines with or without programming expertise to easily and efficiently use the PBPK model to predict the biodistribution of AuNPs with various physicochemical properties. In order to generate simulation results in real time, parallel computing was incorporated into the final PBPK to speed up the simulation and analysis using the “mrgsolve” R package [57]. In addition, the in silico multivariate regression-based QSAR model (a built-in module within the PBPK model) allows researchers to predict the kinetic parameters of AuNPs with different properties for use in the PBPK model simulation. This tool will help the design and testing of newly synthesized NP-based drug delivery and therapeutic systems, which could help make informed decisions on which nanomedicine formulations should proceed to a preclinical trial. This tool can also be used to predict target organ dosimetry of different types of AuNPs to support risk assessment of AuNPs-based products. This proposed new route-specific approach for developing multi-route PBPK models for NPs represents a revolutionary change in the methodology of PBPK modeling for NPs, and can be extrapolated to other types of NPs. Additionally, the developed Nano-iPBPK web-based application represents a significant advancement in the field of nanomedicine and nanotoxicology and represent a future direction in the use of PBPK models for risk assessment of NPs.

## Methods

### Pharmacokinetic data

Pharmacokinetic data of different types of AuNPs in adult rats following various routes of administration including IV [33], oral [34], IT [35], and IH [36] were used in the model development. In brief, 8–10 weeks old female Wistar-Kyoto rats (~ 250 g) were gently administered by syringe solutions containing low-dose suspensions of monodisperse AuNPs of various sizes (1.4, 5, 18, 80, or 200 nm) via the tail vein (3–33 μg per rat) [33], intraesophageal instillation (3–29 μg per rat) [34], and intratracheal instillation (3–41 μg per rat) [35]. In addition, aerosolized AuNPs with a count median diameter (CMD) of 23 nm were inhaled intratracheally by female rats for 2 h with estimated mass concentration of 1.1 mg/m^3^ via an endotracheal tube through a rodent inhalation apparatus as described previously [58, 59]. All AuNPs in suspensions were radioactively labeled with ^198^Au by neutron irradiation before administration by IV, oral, and IT routes. For inhalation (i.e., IH), the ^195^Au radio-labeled AuNP aerosol was freshly produced by spark-ignition between two gold electrodes that had been proton irradiated [59]. Radioactivity of dissected tissue samples in rats was measured by gamma-spectroscopy and converted into tissue concentrations. All biodistribution data in rats were reported at 1 h and 24 h after IV, oral, and IT administrations except for rats receiving 1.4 nm and 18 nm AuNPs intratracheally where additional data at 3 h after exposure were also collected. Tissue distribution data in rats inhaling 23 nm AuNPs were measured at 0, 4, 24, 168, and 672 h after inhalation exposure. Table 1 lists key study information on these selected pharmacokinetic datasets. Additional information on the physicochemical properties of the studied AuNPs, such as dose, hydrodynamic diameter, surface area, surface functionalization, Zeta potential, and number of AuNPs is summarized in Additional file 1: Table S1. It is important to note that the hydrodynamic diameter was used in this study to reflect the size of particles in the solution or biological media. Please refer to the original publications for additional information about these studies [33–36].

### PBPK model development and preliminary calibration

Multi-route PBPK models were developed based on size-specific AuNPs and all simulations were performed using Berkeley Madonna™ (Version 8.3.23.0, University of California at Berkeley, CA, USA) to obtain visually reasonable fits to the pharmacokinetic data. The PBPK model was developed based on our recently published rat PBPK model for AuNPs [8] with minor modifications in compartments and administration routes according to newly available pharmacokinetic data [33–36] and on the NP tissue distribution pattern in lungs after inhalation. Briefly, the PBPK model consisted of seven compartments including blood, lungs, liver, spleen, GI tract, kidneys, and remaining tissues (Additional file 1: Fig. S1). In the present model, tissue distribution of AuNPs was described by considering membrane-limited transcapillary transport, uneven distribution between blood and tissue using the term “distribution coefficient”, endocytosis/phagocytosis using non-linear functions, and exocytic release of AuNPs [8, 19, 32]. To be more specific, this study termed a subcompartment “endocytic/phagocytic cells (PCs)” inside a tissue compartment containing a variety of cells that involve in endocytosis of AuNPs, such as Kupffer cells and hepatocytes in the liver, macrophages, epithelial cells and fibroblasts in lungs, and splenic macrophages [16, 60]. Note that the present model cannot distinguish different endocytosis/phagocytosis uptake mechanisms. This study used the term “endocytosis” as an operational term to represent different types of cellular uptake mechanisms. The rate constants describing urinary, biliary, and fecal excretion, respiratory transport, and intestinal reabsorption were considered to be a first-order process to reduce model complexity. Additional details on the model development and preliminary calibration were described in Additional file 1.

### Model parameterization and optimization

The Bayesian approach with Markov chain Monte Carlo (MCMC) simulation was conducted by *Stan* [61] and used to further optimize the model with the pharmacokinetic data listed in Table 1 for each size of the AuNPs. To improve the efficiency of the model optimization and provide the basis of the prior knowledge in the MCMC algorithm, preliminary model calibration was conducted by optimally fitting size- and route-specific physicochemical parameters with the “Curve Fitting Module” in Berkeley Madonna. These initial estimated parameter values were used as priors in the Bayesian-MCMC optimization. Details regarding the preliminary model calibration were described in Additional file 1.

The uncertainty and variability of model parameters in the multi-route NP PBPK model for each size were characterized by using a hierarchical Bayesian framework (Fig. 1c) [37, 62, 63]. Because the focus of this study was to characterize/optimize some key and unmeasured or unknown parameters rather than estimating every possible parameter, only the maximum uptake rate, uptake capacity, release rate of phagocytic cells and elimination rate constant parameters in target organs (e.g., liver, spleen, lungs, and kidneys) were included in the Bayesian-MCMC analysis. The Bayesian-MCMC processes were described by a three-stage hierarchical model to characterize both the inter-study variability and uncertainty of the selected parameters, as well as the quantification of the residual variability which was mainly a result of model misspecification or measured error. The model likelihood was described in first stage of model, where the proportional errors were modeled by a log-normal distribution (Eq. 1).

1 $$p({\text{log}}(y_{ij} |\theta_{i} ,\sigma^{2} ) \propto N\left( {\log \left( {f\left( {D_{i} ;t_{ij} ;\theta_{i} } \right)} \right),\sigma^{2} } \right)$$

Assuming that *N* blood or tissue samples from animal studies, indexed by *j*, were drawn for each of the *M* individuals indexed by *i*. Let the *j*th measurement of individual *i* be denoted by $y_{ij}$, the associated time by $t_{ij}$ and the related individual dose by $D_{i}$. Denote the p-dimensional vector of selected parameters of individual *i* as $\theta_{i}$, the residual variance of the model as $\sigma^{2}$, and the $f\left( \cdot \right)$ was used to represent the model function (i.e., the PBPK model).

The second stage of model was used to describe the individual level, where log-normal population mean ($\theta_{{lr_{i} }}$) was assumed for all selected parameters (Eq. 2).

2 $$\begin{gathered} p\left( {\theta_{{tr_{i} }} {|}\mu ,\sum^{2} } \right) = MVN_{p} \left( {\mu , \sum^{2} } \right) \hfill \\ \theta_{i} = e^{{\theta_{{tr_{i} }} }} \hfill \\ \end{gathered}$$

where $MVN_{p}$ can be seen as for the *p*-variate multivariate normal distribution, $\mu$ is the population mean and $\sum^{2}$ is the population variance–covariance matrix (size: p x p).

The third stage consisted of distribution assumption of the population level (i.e., priors placed on the population parameters) (Eq. 3).

3 $$\begin{aligned} & p\left( \mu \right) = MVN_{p} \left( {M, S^{2} } \right) \\ & {\updelta }^{2} = diag\left( s \right) \cdot C \cdot diag\left( s \right) \\ & p\left( s \right) = N_{half} \left( {0,1} \right) \\ & p\left( C \right) = LKJ\left( a \right) \\ & p\left( \sigma \right) = N_{half} \left( {0,1} \right) \\ \end{aligned}$$

where the prior distribution for the population mean (*μ*) was specified based on the hyperparameter mean value (*M*) and the variance (*S*^*2*^). The M value for each model parameter was taken from the literature or estimated from the preliminary model calibration (Additional file 1: Tables S2 and S3); s is a vector of dimension *p*, $N_{half}$ is the half-normal distribution, and *C* a prior correlation matrix. Based on the previous study [64], a Lewandowski–Kurowicka–Joe (LKJ) prior was assigned to the correlation matrix. The estimated posterior parameters included cellular uptake and release rate constants for lungs, liver, kidneys, and spleen, as well as the urinary elimination rate constant parameters (Additional file 1: Tables S4 for oral, for IV, S6 for IT, and S7 for IH exposures).

### Convergence of model parameters estimates

The model parameters sampled from MCMC simulations within the Bayesian framework should be inspected to verify that equilibrium has been achieved. The equilibrium of the parameters is called “convergences of posterior parameters” and could be diagnosed by the potential scale reduction factor ($\hat{R}$) [65]. With each MC chain achieves the convergences (the MC chains move together and towards to the common distribution), the $\hat{R}$ ratio is decreased to the unity. The $\hat{R}$ value of 1.2 or less for each parameter is typically considered as a criterion of acceptable convergence [66].

### Sensitivity analyses

Local sensitivity analyses were conducted to identify highly influential parameters governing the overall pharmacokinetics after individual administration routes. Specifically, each parameter (*p*) was increased by 1% at a time, and the corresponding area-under-the-concentration curve (AUC) of AuNPs in artery blood, lungs, liver, spleen, GI tracts, kidneys, and remaining were computed for 5 nm AuNPs at 24 and 672 h after IV, oral, and IT administrations, as well as for 23 nm AuNPs after IH administration. Normalized sensitivity coefficient (NSC) was calculated by dividing the relative change in AUC (*d*AUC/AUC) with the relative change in each parameter (*dp*/*p*) [19]. Parameters with at least one calculated absolute NSC value of around or greater than 0.5 were considered highly sensitive.

### Comparison of the traditional route-to-route extrapolation approach for small molecules versus the new route-specific approach for NPs

To test our hypothesis to see whether the traditional route-to-route extrapolation approach for small molecules is applicable for NPs, we extrapolated the PBPK model from IV route to other routes using the traditional approach, i.e., keeping all chemical-specific parameters the same as in the IV route and only adding necessary route-related parameters such as the oral absorption rate. We then compared the results from the models derived from the traditional approach versus the new approach proposed for NPs described above.

### Model application: multivariate linear regression analyses to build a quantitative structure–activity relationship (QSAR) model

The optimized values for key biodistribution parameters were then used to develop an in silico QSAR model to describe the relationship between the key biodistribution parameter values and the physicochemical properties of AuNPs. Specifically, available measured physicochemical characteristics of administered AuNPs listed in Additional file 1: Table S1 were included in the multiple regression-based QSAR models as variables with or without natural logarithms (as appropriate), including hydrodynamic diameter size (HD), surface area (SA), Zeta potential (ZP), administered NP numbers (log(NPs)), log(HD) and log(SA)). The biodistribution parameters selected for the regression analyses were based on the sensitivity analysis results and included maximum uptake rate, endocytic/phagocytic uptake capacity, exocytosis rate, biliary, urinary and fecal excretion rate constants (Additional file 1: Tables S8 for oral, S9 for IV, S10 for IT, and S11 for IH exposures). The Bayesian information criteria (BIC), adjusted *R*^2^ and *p* value (*p* < 0.05 was considered statistically significant) were chosen to evaluate the adequacy of stepwise constructed regression models.

### Model evaluation/validation with independent datasets

The evaluation/validation of the PBPK model was performed by comparing model simulations with independent pharmacokinetic studies that were not used in the model calibration. The predictability of the model was considered be adequate and reasonable if the simulated values are within a factor of two of the measured mean value based on the basis of World Health Organization PBPK modeling guidance [38]. Six IV datasets for AuNPs were extracted from previous studies [47, 48] and used for model validation (Table 1). These datasets covered dose of 0.7–1 µg per rat, short-term (24 h) and long-term (28 days) exposure durations, size of 16.1–34.9 nm and other information (Table 1). The NPs were either uncoated or coated with citrate, 11-mercaptoundecanoic acid (11-MUA), Cys-Ala-Leu-Asn-Asn (CALNN), Cys-Ala-Leu-Asn-Asp (CALND) and Cys-Ala-Leu-Asn-Ser (CALNS). A summary of the physicochemical properties of the selected AuNPs is available in Additional file 1: Table S14.

### Translation of the combined PBPK model with the in silico multivariate regression-based QSAR model into a user-friendly Nano-iPBPK interface

Two different ODE solver packages, “mrgsolve” and “STAN”, were used to solve the differential equations in the R code. The “STAN” package was used for the optimization of model parameters within Bayesian framework. Next, the optimized model parameters were incorporated into the “mrgsolve” package for coding the user-friendly Nano-iPBPK interface. The Nano-iPBPK interface was constructed with the “Shiny” package based on the R model code. A screenshot of the design of the Nano-iPBPK interface is shown in Fig. 7. Please refer to Additional file 1 for more details about this interface, including a detailed tutorial and an example output report.

## Supplementary Information

**Additional file 1. Section 1**: Equations and codes for the multiple-route AuNPs PBPK model. **Section 2**: Methods on preliminary model calibration. **Section 3**: Methods on estimation of posterior parameter within Bayesian framework. **Section 4**: Model calibration results. Section 5. Sensitivity analysis methods. **Table S1**: Physicochemical characteristics of gold nanoparticles and dosing information of pharmacokinetic studies used in the PBPK model. **Table S2**: Physiological parameters used in the multi-route PBPK model for gold nanoparticles in rats. **Table S3**: Gold nanoparticle-specific parameters used in the PBPK model after various routes of administration in rats. **Table S4**: Posterior uncertainty distributions for the population mean (µ) and variance (${\Sigma }^{2}$) of the PBPK model parameters following oral administration. **Table S5**: Posterior uncertainty distributions for the population mean (µ) and variance (${\Sigma }^{2}$) of the PBPK model parameters following intravenous administration. **Table S6**: Posterior uncertainty distributions for the population mean (µ) and variance (${\Sigma }^{2}$) of the PBPK model parameters following intratracheal instillation. **Table S7**: Posterior uncertainty distributions for the population mean (µ) and variance (${\Sigma }^{2}$) of the PBPK model parameters following endotracheal inhalation for 23 nm AuNPs. **Table S8**: Normalized sensitivity coefficients (NSCs) of highly influential parameters after oral administration to 5 nm AuNPs. **Table S9**: Normalized sensitivity coefficients (NSCs) of highly influential parameters after intravenous (IV) administration to 5 nm AuNPs. **Table S10**: Normalized sensitivity coefficients (NSCs) of highly influential parameters after intratracheal instillation to 5 nm AuNPs. **Table S11**: Normalized sensitivity coefficients (NSCs) of highly influential parameters after inhalation administration to 23 nm AuNPs. **Table S12**: Final multivariate linear regression models describing relationships between the physicochemical properties of AuNPs and biodistribution parameters following oral gavage. **Table S13**: Final multivariate linear regression models describing relationships between the physicochemical properties of AuNPs and biodistribution parameters following intratracheal instillation. **Table S14**: Main physicochemical characteristics of the AuNPs in the selected pharmacokinetic studies used for model evaluation in this study. **Table S15**: Model-predicted and measured amount of AuNPs in blood, GI, Kidneys, Liver, Lungs and spleen. **Figure S1**: Schematic diagram of the multi-route physiologically based pharmacokinetic (PBPK) model for 1.4, 5, 18, 23, 80, and 200 nm gold nanoparticles (AuNPs) in adult rats. **Figure S2**: PBPK model simulation results compared with 24-h IV-based pharmacokinetic data of 1.4, 5, 18, 80, and 200 nm AuNPs in healthy rats. **Figure S3**: PBPK model simulation results compared with 24-h pharmacokinetic data of 5, 18, 80, and 200 nm AuNPs in rats following oral gavage. **Figure S4**: PBPK model simulation results compared with 24-h pharmacokinetic data of 5, 18, 80, and 200 nm AuNPs in rats following oral gavage. **Figure S5**: PBPK model calibration with 28-d pharmacokinetic data in rats after inhalation of 23 nm aerosolized AuNPs via endotracheal tube for 2 h. **Figure S6**: PBPK model calibration results with pharmacokinetic data of 1.4, 5, 18, 80 and 200 nm AuNPs in rats after intravenous injection. **Figure S7**: PBPK model calibration results with pharmacokinetic data of 5, 18, 80 and 200 nm AuNPs in rats after oral administration. **Figure S8**: PBPK model calibration results with pharmacokinetic data of 1.4, 5, 18, 80 and 200 nm AuNPs in rats after intratracheal instillation. **Figure S9**: Heat map plot of the normalized sensitivity coefficient (NSC) for comparative sensitivity analyses for 18, 80 and 200 nm AuNPs at 24 h and 672 h following oral administration, IV injection, and IT instillation. **Figure S10**: Comparisons of model prediction (x-axis) with observed data (y-axis) for the validation datasets. **Section 8**: Tutorial for Nano-iPBPK interface. **Section 9**: Supplementary references.

## Data Availability

All raw data used for model development are available from the original publications [33–36] and are also provided at https://github.com/UFPBPK/Nano-iPBPK. The Nano-iPBPK web-based application is available at https://pbpk.shinyapps.io/NanoiPBPK/. Nano-iPBPK application can be deployed by users at their own web server. The corresponding source code is available at https://github.com/UFPBPK/Nano-iPBPK.

## Abbreviations

AuNPs: Gold nanoparticles
Cl: Confidence interval
HD: Hydrodynamic diameter
ID: Initial dose
IH: Endotracheal inhalation
IPLD: Initial peripheral lung dose
IT: Intratracheal instillation
IV: Intravenous
IVIVE: In vitro to in vivo extrapolation
Nano-iPBPK: Nanoparticle interactive physiologically based pharmacokinetic
NPs: Nanoparticles
MCMC: Markov chain Monte Carlo
O/P ratio: Observed-to-predicted ratio
PBPK: Physiologically based pharmacokinetic
QSAR: Quantitative structure–activity relationship
RMSE: Root mean square error
SA: Surface area
