# Supplementary material for: Development of a multi-route physiologically based pharmacokinetic (PBPK) model for nanomaterials: a comparison between a traditional versus a new route-specific approach using gold nanoparticles in rats
Source: Part Fibre Toxicol. 2022 Jul 8;19:47. doi: 10.1186/s12989-022-00489-4 (PMC9264615; doi:10.1186/s12989-022-00489-4)
Supplement: Supplementary file 1 — Additional file 1. Section 1: Equations and codes for the multiple-route AuNPs PBPK model. Section 2: Methods on preliminary model calibration. Section 3: Methods on estimation of posterior parameter within Bayesian framework. Section 4: Model calibration results. Section 5. Sensitivity analysis methods. Table S1: Physicochemical characteristics of gold nanoparticles and dosing information of pharmacokinetic studies used in the PBPK model. Table S2: Physiological parameters used in the multi-route PBPK model for gold nanoparticles in rats. Table S3: Gold nanoparticle-specific parameters used in the PBPK model after various routes of administration in rats. Table S4: Posterior uncertainty distributions for the population mean (µ) and variance (${\Sigma }^{2}$Σ2) of the PBPK model parameters following oral administration. Table S5: Posterior uncertainty distributions for the population mean (µ) and variance (${\Sigma }^{2}$Σ2) of the PBPK model parameters following intravenous administration. Table S6: Posterior uncertainty distributions for the population mean (µ) and variance (${\Sigma }^{2}$Σ2) of the PBPK model parameters following intratracheal instillation. Table S7: Posterior uncertainty distributions for the population mean (µ) and variance ( [file 12989_2022_489_MOESM1_ESM.docx]

**Supplementary Information**

**Development of a multi-route physiologically based pharmacokinetic (PBPK) model for nanomaterials: a comparison between a traditional versus a new route-specific approach using gold nanoparticles in rats**

Wei-Chun Chou^1,2,3,7^, Yi-Hsien Cheng^3,4,7^, Jim E. Riviere^3,4,5^, Nancy A. Monteiro-Riviere^4^, Wolfgang G. Kreyling^6^, Zhoumeng Lin^1,2,3,4,^*

^1^Department of Environmental and Global Health, College of Public Health and Health Professions, University of Florida, Gainesville, FL 32610, USA

^2^Center for Environmental and Human Toxicology, University of Florida, Gainesville, FL 32608, USA

^3^Institute of Computational Comparative Medicine, Kansas State University, Manhattan, KS 66506, USA

^4^Nanotechnology Innovation Center of Kansas State, Kansas State University, Manhattan, KS 66506, USA

^5^1Data Consortium, Kansas State University, Olathe, KS 66061, USA

^6^Helmholtz Zentrum München, German Research Center for Environmental Health, Institute of Epidemiology, Ingolstaedter Landstrasse 1, Neuherberg, 85764 Munich, Germany

^7^These authors contributed equally.

Wei-Chun Chou: [w.chou@ufl.edu](mailto:w.chou@ufl.edu); Yi-Hsien Cheng: [yhcheng1987@ksu.edu](mailto:yhcheng1987@ksu.edu); Jim E. Riviere: [jriviere@ksu.edu](mailto:jriviere@ksu.edu); Nancy A. Monteiro-Riviere: [nmonteiro@ksu.edu](mailto:nmonteiro@ksu.edu); Wolfgang G. Kreyling: [kreyling@helmholtz-muenchen.de](mailto:kreyling@helmholtz-muenchen.de); Zhoumeng Lin: [linzhoumeng@ufl.edu](mailto:linzhoumeng@ufl.edu)

* Corresponding author: Department of Environmental and Global Health, College of Public Health and Health Professions, University of Florida, 1225 Center Drive, Gainesville, FL 32610, USA. Email: [linzhoumeng@ufl.edu](mailto:linzhoumeng@ufl.edu). Phone: +1-352-273-9188.

**Table of Contents**

[**1.** **Equations and codes for the multiple-route AuNPs PBPK model** 3](#_Toc103849903)

[**2.** **Preliminary model calibration** 6](#_Toc103849904)

[**3.** **Estimation of posterior parameter within Bayesian framework** 8](#_Toc103849905)

[**4.** **The results for model calibration** 8](#_Toc103849906)

[**4.1 Preliminary model calibration** 8](#_Toc103849907)

[**4.2 Model calibration with Bayesian approach** 10](#_Toc103849908)

[**5.** **Sensitivity analysis** 11](#_Toc103849909)

[**6.** **Supplementary Tables** 13](#_Toc103849910)

[**Table S1.** 13](#_Toc103849911)

[**Table S2.** 15](#_Toc103849912)

[**Table S3.** 17](#_Toc103849913)

[**Table S4** 20](#_Toc103849914)

[**Table S5** 21](#_Toc103849915)

[**Table S6** 23](#_Toc103849916)

[**Table S7** 25](#_Toc103849917)

[**Table S8** 26](#_Toc103849918)

[**Table S9.** 27](#_Toc103849919)

[**Table S10** 28](#_Toc103849920)

[**Table S11** 29](#_Toc103849921)

[**Table S12.** 30](#_Toc103849922)

[**Table S13.** 31](#_Toc103849923)

[**Table S14.** 32](#_Toc103849924)

[**Table S15** 33](#_Toc103849925)

[**7.** **Supplementary Figures** 34](#_Toc103849926)

[**Fig. S1** 34](#_Toc103849927)

[**Fig. S2** 35](#_Toc103849928)

[**Fig. S3** 36](#_Toc103849929)

[**Fig. S4** 37](#_Toc103849930)

[**Fig. S5** 38](#_Toc103849931)

[**Fig. S6** 39](#_Toc103849932)

[**Fig. S7** 40](#_Toc103849933)

[**Fig. S8** 41](#_Toc103849934)

[**Fig. S9** 42](#_Toc103849935)

[**Fig. S10** 43](#_Toc103849936)

[**8.** **Tutorial for Nano-iPBPK** 45](#_Toc103849937)

[**8.1** **Introduction** 45](#_Toc103849938)

[**8.2** **Instruction** 45](#_Toc103849939)

[**8.3** **Example** 47](#_Toc103849940)

[**9.** **References** 51](#_Toc103849941)

1. **Equations and codes for the multiple-route AuNPs PBPK model**

The equations below, along with the parameters in Tables S1-S3, specify the AuNPs PBPK model in Berkeley Madonna. Since the model was built primarily based on earlier models [1, 2], only key or new mathematical equations are described in detail below. The distribution of AuNPs between sub-compartments of capillary blood, tissue interstitium, and phagocytic cells (PCs) in the spleen, lung, kidney, GI tract, and remaining tissues were described using the following equations:

$\frac{d{A_{Blood}}_{i}}{dt}=Q_{i}\times\left( C_{a}-{CV}_{i} \right)-{PA}_{i}\times{CV}_{i}+\frac{{PA}_{i}\times{C_{T}}_{i}}{P_{i}}$ (S1)

$\frac{dA_{T_{i}}}{dt}={PA}_{i}\times{CV}_{i}-\frac{{PA}_{i}\times{C_{T}}_{i}}{P_{i}}-K_{up_{i}}\times A_{T_{i}}+K_{exo_{i}}\times A_{PC_{i}}$ (S2)

$\frac{dA_{{PC}_{i}}}{dt}=K_{up_{i}}\times A_{T_{i}}-K_{exo_{i}}\times A_{PC_{i}}$ (S3)

$K_{up_{i}}=K_{max_{i}}\times(1-\frac{A_{PC_{i}}}{A_{cap_{i}}\times V_{i}})$ (S4)

where $A_{Blood_{i}}$ [μg] and$A_{T_{i}}$ [μg] represents amount of AuNPs in the capillary blood and tissue interstitium of the organ *i*, respectively; *Q_i_* [L/h] is the regional blood flow to the organ *i*; *C_a_* [μg/L] is the concentration of AuNPs in the arterial blood; *CV_i_* [μg/L] is the concentration of AuNPs in the venous blood in the organ *i*; *PA_i_* [L/h] is the permeability area cross product between the capillary blood and tissue membrane in the organ *i* (*PA_i_* is approximated to the product of permeability coefficient between capillary blood and tissue membrane [*PAC_i_*: unitless] and regional blood flow [*Q_i_*: L/h]); $C_{T_{i}}$ [μg/kg tissue] is the concentration of AuNPs in the tissue interstitium of the organ *i*; *P_i_* [unitless] is the tissue:blood distribution coefficient for the organ *i*; $K_{up_{i}}$ [1/h] is the uptake rate of endocytic or phagocytic cells that can be estimated based on the maximum uptake rate constant [$K_{max_{i}}$: 1/h] and capacity [$A_{cap_{i}}$: μg/kg tissue] of endocytic or phagocytic cells, amount of AuNPs endocytosed [$A_{{PC}_{i}}$: μg], and the organ volume [*V_i_*: kg]; and $K_{{exo}_{i}}$ [1/h] is the exocytic release rate of AuNPs from endocytic or phagocytic cells. Similar equations were used to simulate the distribution of AuNPs among different sub-compartments in the liver. The only difference in the liver compared to other organs is that Kupffer cells (macrophages) exists and can directly phagocytize AuNPs from the blood sub-compartment because Kupffer cells are located in the sinusoids and directly exposed to the blood [3, 4].

Shortly after inhalation, AuNPs will be transported either from head airways to the brain contained in remaining tissues via the olfactory bulb, or from head airways and tracheobronchial region of lungs towards the larynx through mucociliary clearance and then swallowed into the GI tract lumen and eventually excreted via the feces [5-7]. To develop a general model integrating multiple exposure scenarios, the olfactory migration pathway of AuNPs remained in our PBPK modeling framework even though it was zero followed by IT and IH routes of exposure which excluded head airway exposures [5, 7]. Therefore, we differentiated the respiratory system accordingly as upper and lower airways (i.e., tracheobronchial and pulmonary region (with PCs), pulmonary interstitium (with PCs) and capillary blood). In addition, to describe both oral and inhalation administrations, the GI tract was divided into 4 subcompartments: capillary blood, GI tract lumen, interstitium, and PCs (Fig. S1).

Specifically, uptake of AuNPs and transportation processes inside upper airways and lungs (Eqs. S5 – S9) after inhalation were described using the following equations:

$\frac{dA_{ua}}{dt}=-(K_{uab}+K_{uagi})\times A_{ua}$ (S5)

$\frac{dA_{tra}}{dt}=K_{pulPCtra}\times A_{PC_{pul}}-K_{tragi}\times A_{tra}$ (S6)

$\frac{dA_{pul}}{dt}=K_{inpul}\times A_{in}-K_{pulin}\times A_{pul}-K_{up_{pul}}\times A_{pul}+K_{exo_{pul}}\times A_{PC_{pul}}$ (S7)

$\frac{dA_{in}}{dt}=K_{pulin}\times A_{pul}-K_{inpul}\times A_{in}+{PA}_{Lu}\times{CV}_{Lu}-\frac{{PA}_{Lu}\times C_{in}}{P_{Lu}}$

$-K_{up_{in}}\times A_{in}+K_{exo_{in}}\times A_{PC_{in}}$ (S8)

$\frac{d{A_{blood}}_{Lu}}{dt}=Q_{C}\times\left( C_{a}-{CV}_{Lu} \right)-{PA}_{Lu}\times{CV}_{Lu}+\frac{{PA}_{Lu}\times C_{in}}{P_{Lu}}$ (S9)

where *A_ua_* [μg], *A_tra_* [μg], *A_pul_* [μg], *A_in_* [μg], and ${A_{blood}}_{Lu}$ [μg] represent distributed or transported amounts of AuNPs to upper airways, tracheobronchial region, pulmonary region, lung interstitium, and capillary blood of lungs in rats, respectively. The initial values of A_ua_ [μg], A_tra_ [μg] and A_pul_ [μg] were defined as the administrated mass of AuNPs (Table S1) multiplied by the deposition fractions in upper airways (F_ua_), tracheobronchial (F_tra_) and pulmonary (F_pul_) regions, respectively (Table S2). The deposition fractions in different lung regions were estimated by the MPPD model. However, the MPPD model didn’t include the module of intratracheal instillation of a bulk liquid (the method used in our data for IT administration route). Instead, we estimated the deposition fraction of AuNPs following IT exposure by selecting the “endotracheal” option under the “Breathing Scenario” module in the MPPD model. This is one of the limitations of our study.

*K_uab_* [1/h] and *K_uagi_* [1/h] are transportation rate constants from upper airways to brain and GI lumen, respectively; *K_pulPCtra_* [1/h] is the transportation rate constant of inactive pulmonary PCs to the tracheobronchial region by the mucociliary escalator; *K_tragi_* [1/h] is the transfer rate constant of AuNPs from tracheobronchial region to the GI lumen; *K_pulin_* [1/h] and *K_inpul_* [1/h] represent interexchange rate constants from pulmonary region to lung interstitium and from lung interstitium to the pulmonary region, respectively; *C_in_* [μg/kg] is the concentration of AuNPs in lung interstitium; *Q_C_* [L/h] is the cardiac output.

The mass transport of AuNPs in the GI lumen was described by considering various exposure routes, including oral gavage, IT and IH exposures using the equation below:

$\frac{dA_{lumen}}{dt}=K_{uagi}\times A_{ua}+K_{tragi}\times A_{tra}-K_{GIb}\times A_{lumen}+K_{bile}\times C_{T_{Liver}}$

$-K_{feces}\times A_{lumen}$ (S10)

where *A_lumen_* [μg] is the amount of AuNPs transported to the GI lumen; *K_GIb_* [1/h] is the absorption rate constant of AuNPs from the GI lumen to the capillary blood in the GI tract; *K_bile_* [L/h] is the biliary excretion rate of liver; $C_{T_{Liver}}$ [μg/kg] is the concentration of AuNPs inside liver interstitium; *K_feces_* [1/h] is the fecal elimination rate constant from the GI lumen.

1. **Preliminary model calibration**

To provide the basis of the prior knowledge in the Bayesian analysis, the preliminary model calibration was conducted by optimally fitting size- and route-specific physicochemical parameters with “Curve Fitting Module” in Berkeley Madonna. Specifically, to develop the most parsimonious model for different exposure routes, most physiological parameters were kept consistent with the literature [8, 9] except for the tissue or organ volumes that were directly calculated based on the selected pharmacokinetic studies [10-13]. Whereas for physicochemical parameters, distribution coefficient and permeability coefficient, generic values were used as described previously [2] to create the most parsimonious PBPK model unless otherwise mentioned (Tables S2-S3). Taking into account that various administration routes and AuNP sizes may have differential target tissue distributions in rats, size- and route-specific physicochemical parameters were optimally fitted using the Curve Fitting Module in Berkeley Madonna. The estimated parameters included uptake, transportation and excretion rate constants for lungs, liver, kidneys, and GI tract, as well as distribution coefficients, permeability coefficients, and endocytosis- and exocytosis-related parameters (referred to Tables S2–S3). To estimate the deposition fraction (DF) of AuNPs in tracheobronchial (TB) and pulmonary (P) region of lungs, respectively, following IT and IH administrations, the multiple-path particle dosimetry (MPPD) model was employed (version 3.04, ARA Inc., VA, USA). Specifically, asymmetric Sprague-Dawley rat model was selected to estimate lung region-specific DF by assigning measured body weight (BW) of rats followed with defaulted or estimated breathing frequency and tidal volume in adult rats intratracheally instilled or endotracheally inhaled with AuNPs, respectively (Table S3). The relative deposition fraction of AuNPs in TB and P were thus estimated as DF_TB_/(DF_TB_+DF_P_) and DF_P_/(DF_TB_+DF_P_), respectively. In addition, the “SQUAREPULSE” function in Berkeley Madonna was implemented to describe the fast- and slow-phase transportation rate constant of AuNPs from the respiratory tract to GI tract as $K_{slow}+K_{fast}\times SQUAREPULSE\left( t_{0},t_{D} \right)$, where $K_{slow}$ is the baseline transportation rate constant, $t_{0}$ and $t_{D}$ are the start time and duration, respectively, for fast-phase mucociliary clearance rate constant ($K_{fast}$).

1. **Estimation of posterior parameter within Bayesian framework**

The Bayesian approach with Markov chain Monte Carlo (MCMC) simulation was conducted by *Stan[14]*. Based on the optimized parameter values from preliminary calibration, the prior distributions were defined and incorporated into the parameter optimization within Bayesian framework. Four Markov chains of 4,000 iterations each, for the IV, Oral, IT and inhalation, respectively, were run with the first 2,000 iterations as “burn-in” iterations and the last 2,000 iterations were used as output iterations to check convergences. Corrected potential scale reduction factors ($\hat{R}$) were calculated for the four chains to diagnose the convergences of Markov chains based on the method of Brooks and Gelman [15]. $\hat{R}$ values were used to assess whether the independent MCMC chains have converged to a common distribution. The $\hat{R}$ values of population mean (µ) and population variance (∑2) in the model following oral, IV, IT and IH administration are provided in Tables S4-S7.

1. **The results for model calibration**

**4.1 Preliminary model calibration**

Based on the optimized parameters from the initial model calibration, the model simulations of short-term (i.e., IV, oral, and IT) and long-term (i.e., IH) kinetic profiles were compared with measured amounts (given in percent of the initial dose [%ID] or percent of the initial peripheral lung dose [%IPLD]) in selected tissues and organs of rats after different routes of administration for different sizes of AuNPs (Figs S2–S5). Specifically, PBPK model predicted-values were in good agreement with reported size-specific tissue distribution data in blood, liver, and spleen at 24 h after IV administration as indicated by the coefficient of determination (*R*^2^) of 0.88 – 0.95 obtained from the linear regression analyses (Fig. S2). The calibrated PBPK model adequately predicted the biodistribution of AuNPs after IV administration in most tissues except for slight over estimation in lungs (Fig. S2D), kidneys (Fig. S2P), and remaining tissues (Fig. S2D and T), i.e., deviations from the unity of linear regression line.

In rats orally administered 5 – 200 nm AuNPs, PBPK model simulation results reasonably correlated with the observed amounts of AuNPs in the primary tissues, e.g., blood and GI tract with acceptable accuracy (*R*^2^ > 0.75) (Fig. S3). The PBPK model simulations showed that more than 99% of the administered AuNPs was deposited in the GI tract, which is consistent with the measured data [11]. The model had slight under estimation of the amount of 18 and 200 nm AuNPs in the kidney and for 200 nm AuNPs in remaining tissues, as well as an overestimation for 18 nm AuNPs in liver (Fig. S3F and L).

In rats after IT administration, pharmacokinetic data measured at 1, 3, and 24 h (1.4 and 18 nm) as well as at 1 and 24 h (5, 80, and 200 nm) were reasonably described by the size-dependent PBPK model with an overall *R*^2^ of 0.79-0.92 (Fig. S4). In general, the calibrated PBPK model well captured the biodistribution data in primary tissues (lungs and GI tracts) of rats following IT exposure, but overestimated the distribution and translocation of AuNPs to the secondary tissues, including blood, liver, kidneys, spleen, and remaining tissue (Fig. S4F, I, L, and O).

Comparing PBPK model-predicted results with tissue distribution data in rats inhaled with 23 nm AuNPs, the calibrated PBPK model adequately characterized AuNP distribution in both primary tissue (e.g., lungs) and secondary tissues (e.g., liver, spleen, and kidneys) with acceptable accuracy (*R*^2^ > 0.85) (Fig. S5). To be more specific, the calibrated PBPK model could not fully capture AuNP distribution to blood, GI tract, and remaining tissues at earlier time points, but properly capture the kinetic profiles in these organs at later time points (Fig. S5B, E, and F).

**4.2 Model calibration with Bayesian approach**

Based on the posterior parameters after Bayesian-MCMC analysis, the probabilistic time-course predictions [median and 95% confidence interval (CI)] were generated and compared with measured amounts in selected tissues and organs of rats after different routes of administration for different sizes of AuNPs (Fig. 4, Figs. S6-S8). The final PBPK model adequately predicted the biodistribution of AuNPs after IV administration in most tissues except over estimations in lungs for 1.4 nm AuNPs, kidneys for 200 nm AuNPs, and remaining tissues for 200 nm AuNPs (Fig. S6). In rats orally administered 5 – 200 nm AuNPs, PBPK model simulations showed that the model had slight under estimation of the amount of 18 and 200 nm AuNPs in the kidney and for 200 nm AuNPs in remaining tissues, as well as an overestimation for 18 nm AuNPs in liver (Fig. S7). However, it is worth to note that after oral gavage, the distribution of AuNPs to other organs was rather minor (~0.1% ID). Thus, this slight under or over estimation did not substantially alter the overall model simulation results.

In rats after IT administration, the PBPK model well captured the biodistribution data in primary tissues (lungs) of rats following IT exposure, but it overestimated the distribution and translocation of AuNPs to the secondary tissues, including blood, liver, kidneys, spleen, and remaining tissue (Fig. S8). This might be due in part to the high distribution or retention in primary tissues after 1 h for rats instilled with AuNPs, adding up to ~99% IPLD. Additionally, except for rats instilled with 1.4 nm AuNPs, other pharmacokinetic profiles (i.e., 5, 18, 80, and 200 nm) demonstrated that comparing to AuNPs retained in lungs, AuNPs translocation to the secondary tissues were minor with a summation of <0.2% IPLD.

Comparing PBPK model-predicted results with tissue distribution data in rats inhaled with 23 nm AuNPs, the calibrated PBPK model could not fully capture AuNP distribution to lungs, liver, spleen and remaining tissues, but properly capture the kinetic profiles in blood and kidney (Fig. 4). The reasons of the differences between simulated and measured pharmacokinetic profiles were unclear. These results suggest that there might be other unknown mechanisms involved in deposition and translocation of AuNPs in the respiratory system of rats that have not been characterized in this model.

1. **Sensitivity analysis**

Local sensitivity analyses were conducted to identify highly influential parameters governing the overall pharmacokinetics after individual administration routes. Specifically, each parameter (*p*) was increased by 1% at a time, and the corresponded area-under-the-concentration curve (AUC) of AuNPs in artery blood, lungs, liver, spleen, GI tracts, kidneys, and remaining were computed for 5 nm AuNPs at 24 and 672 h after IV, oral, IT and IH administrations (except 23 nm for IH). Normalized sensitivity coefficient (NSC) was calculated by dividing the relative change in AUC (*d*AUC/AUC) with the relative change in each parameter (*dp*/*p*) [1]. Parameters with at least one calculated absolute NSC value of around or greater than 0.5 were considered highly sensitive.

1. **Supplementary Tables**

| **Table S1.** | Physicochemical characteristics of gold nanoparticles and dosing information of pharmacokinetic studies used in the PBPK model. | | | | | | |
| --- | --- | --- | --- | --- | --- | --- | --- |
| AuNP core diameter (nm) | | **1.4** | **5** | **18** | **80** | **200** | **23** |
| Ligand (charged surface group) ^a-c^ | | TPPMS (SO_3_^-^) | TPPMS (SO_3_^-^) | TPPMS (SO_3_^-^) | TPPMS (SO_3_^-^) | TPPMS (SO_3_^-^) | – |
| Hydrodynamic diameter (nm) ^a-c^ | | 2.9 | 12.1 | 21 | 85 | 205 | – |
| Zeta potential (mV) ^a-c^ | | -20.6±0.5 | -21.1±1.4 | -22.8±3.1 | -22.3±1.6 | -41.3±4.5 | – |
| **Intravenous injection (IV)** ^a^ | |  |  |  |  |  |  |
| Admin. mass of ^198^AuNP per rat (μg) | | 32.9±32.2 | 40.5±5.7 | 3.2±0.6 | 19.9±3.4 | 19.5±3.7 | NA |
| Admin. number of ^198^AuNP per rat | | 1.1±0.2×10^14^ | 3.5±0.4×10^13^ | 5.1±2.6×10^10^ | 3.6±0.5×10^9^ | 2.49±0.2×10^8^ | NA |
| Surface area (cm^2^) | | 25.2±19.8 | 17.6±1.1 | 0.39±0.26 | 0.5±0.26 | 0.27±0.06 | NA |
| Volume of ^198^AuNP (cm^3^) | | 0.16±0.03 | 0.08±0.01 | 2.29±0.28 | 0.19±0.03 | 0.97±0.12 | NA |
| **Oral gavage** ^b^ | |  |  |  |  |  |  |
| Admin. mass of ^198^AuNP per rat (μg) | | 2.6±0.4 | 28.8±3.6 | 23.7±22.9 | 19.6±2.5 | 17.9±1.9 | NA |
| Admin. number of ^198^AuNP per rat | | 3.8±1.7×10^13^ | 2.2±0.2×10^13^ | 4.1±0.1×10^10^ | 3.9±0.1×10^9^ | 3.8±0.0×10^8^ | NA |
| Surface area (cm^2^) | | 2.4±1.0 | 17.3±1.4 | 1.47±1.13 | 0.51±0.29 | 0.25±0.05 | NA |
| Volume of ^198^AuNP (cm^3^) | | 0.14±0.02 | 0.07±0.01 | 1.44±0.23 | 0.13±0.08 | 1.04±0.04 |  |
| **Intratracheal instillation (IT)** ^c^ | |  |  |  |  |  |  |
| Received mass of ^198^AuNP per rat (μg) | | 30.7±20.2 | 40.7±5.3 | 3.0±0.3 | 23.0±3.0 | 21.1±3.4 | NA |
| Received number of ^198^AuNP per rat | | 9.5±1.4×10^13^ | 2.8±0.2×10^13^ | 4.4±0.4×10^10^ | 3.5±0.1×10^9^ | 1.8±0.3×10^8^ | NA |
| Surface area of ^198^AuNP (cm^2^) | | 14.5±9.35 | 13.8±8.62 | 0.28±0.17 | 0.48±0.23 | 0.21±0.02 | NA |
| Volume of ^198^AuNP (cm^3^) | | 0.14±0.02 | 0.04±0.004 | 1.81±0.11 | 0.13±0.01 | 0.92±0.03 |  |
| **Endotracheal inhalation (IH)** ^d^ | |  |  |  |  |  |  |
| Received mass of ^195^AuNP per rat (μg) | | NA | NA | NA | NA | NA | 20.1±9.9 |
| Received number of ^195^AuNP per rat | | NA | NA | NA | NA | NA | 2.4±1.2×10^11^ |
| Surface area of ^195^AuNP (cm^2^) | | NA | NA | NA | NA | NA | 15.8±7.7 |
| Volume of ^195^AuNP (cm^3^) | | NA | NA | NA | NA | NA | 1.04±0.04 |

**Abbreviations:** AuNP, gold nanoparticle; TPPMS, triphenylphosphine m-monosulfonate; NA, not available; Admin., administered.

^a^ Adopted from Hirn et al (2011) [10]. ^b^ Adopted from Schleh et al (2012) [11]. ^c^ Adopted from Kreyling et al (2014) [13]. ^d^ Adopted from Kreyling et al (2018) [12]. Note that the hydrodynamic diameter and Zeta potential were determined in the original aqueous suspensions containing only sulfonated triphenylphosphine after rigorous washing resulting in the final sulfonated triphenylphosphine concentration below 0.1 ng/mL of the AuNP suspension. Dose metrics of particle number, surface area, mass, volume were all derived from the radiometric measurements of the ^195^Au radioactivity of each sample (i.e., each total organ, specimens of bone and soft tissue, a large sample of blood, lavage fluids, and the remaining rest of the carcass; also total urine and feces from immediately after exposure until the rat was killed and dissected). This means that the sum of all samples represents the balanced radioactivity dose of the deposited AuNPs given in Bq and calculated for a fixed date of the [^195^Au]AuNP. Since the specific ^195^Au radioactivity per Au mass was by the specific radioactivity of the irradiated Au-rods used for the spark ignition, vaporization and condensation and was constant in all samples, the dose metrics of mass can easily be calculated. Those metrics of particle number, surface area, volume need assumptions of NP-sphericity, their density and the particle distribution of the aerosol.

| **Table S2.** | Physiological parameters used in the multi-route PBPK model for gold nanoparticles in rats. | | | | | | |
| --- | --- | --- | --- | --- | --- | --- | --- |
| Parameter | | Symbol | Administration route | | | |  |
|  |  |  | IV ^a^ | Oral ^b^ | IT ^c^ | IH ^d^ |  |
| Body weight (kg) | | BW | 0.273 (0.013) ^d^ | 0.273 (0.018) | 0.275 (0.025) | 0.216 (0.014) | Calculated |
| Cardiac output (L/h/kg^0.75^) | | QCC | 15 (Generic) | | | | Brown et al. (1997) [9] |
| Blood flow to organ (fractional cardiac output, unitless) | | | | | | | |
| Lungs | | QLuC | 1 (Generic) | | | | Brown et al. (1997) [9] |
| GI tract | | QGIC | 0.101 (Generic) | | | | Davies and Morris (1993) [8] |
| Liver | | QLC | 0.021 (Generic) | | | | Brown et al. (1997) [9] |
| Spleen | | QSC | 0.009 (Generic) | | | | Davies and Morris (1993) [8] |
| Kidneys | | QKC | 0.141 (Generic) | | | | Brown et al. (1997) [9] |
| Rest of body | | QRC | 0.728 (Generic) | | | | Calculated |
| Tissue or organ volumes (fractional body weight, unitless) | | | | | | | |
| Lungs | | VLuC | 0.006 (0.001) | 0.007 (0.004) | 0.005 (0.001) | 0.004 (0.00004) | Calculated |
| GI tract | | VGIC | 0.100 (0.013) | 0.101 (0.015) | 0.095 (0.012) | 0.094 (0.010) | Calculated |
| Liver | | VLC | 0.034 (0.003) | 0.035 (0.005) | 0.034 (0.004) | 0.036 (0.003) | Calculated |
| Spleen | | VSC | 0.003 (0.001) | 0.004 (0.003) | 0.003 (0.001) | 0.002 (0.0002) | Calculated |
| Kidneys | | VKC | 0.009 (0.001) | 0.009 (0.003) | 0.009 (0.001) | 0.009 (0.001) | Calculated |
| Blood | | VBloodC | 0.067 (0.00006) | 0.072 (0.012) | 0.067 (0) | 0.064 (0.0002) | Calculated |
| Rest of body | | VRC | 0.781 | 0.772 | 0.787 | 0.791 | Calculated |
| Volume fraction of blood in organs (unitless) | | | | | | | |
| Lungs | | BVLu | 0.36 (Generic) | | | | Brown et al. (1997) [9] |
| GI tract | | BVGI | 0.04 (Generic) ^f^ | | | | Brown et al. (1997) [9] |
| Liver | | BVL | 0.21 (Generic) | | | | Brown et al. (1997) [9] |
| Spleen | | BVS | 0.22 (Generic) | | | | Brown et al. (1997) [9] |
| Kidneys | | BVK | 0.16 (Generic) | | | | Brown et al. (1997) [9] |
| Rest of body | | BVR | 0.04 (Generic) ^f^ | | | | Brown et al. (1997) [9] |
| Breath frequency (1/s) | | BF | NA | NA | NA | 1 | Estimated^g^ |
| Tidal volume (L/kg) | | TV | NA | NA | NA | 0.008 | Saiki et al. (1994)[16] |
| Deposition fraction (unitless) | |  |  |  |  |  |  |
| Upper airways | | F_ua_ | NA | NA | 0 | 0 | Estimated^g^ |
| Tracheobronchial | | F_tra_ | NA | NA | 0.1 | 0.16 | Estimated^g^ |
| Pulmonary | | F_pul_ | NA | NA | 0.074 | 0.19 | Estimated^g^ |

**Abbreviation:** GI, gastrointestinal; NA, not applicable.

^a^ Hirn et al (2011) [10]. ^b^ Schleh et al (2012) [11]. ^c^ Kreyling et al (2014) [13]. ^d^ Kreyling et al (2018) [12]. ^e^ Mean (SD). ^f^ Assumed the same as the muscle. ^g^ Parameters estimated from MPPD (v3.04) further applied to estimate relative deposition fraction of AuNPs in tracheobronchial and pulmonary regions of lungs. Tissue or organ volumes (fractional body weight, unitless) were calculated as the ratio of organ weight divided by body weight of the animals assuming the organ density to be 1 g/cm^3^ based on the data from Hirn et al (2011) [10], Schleh et al (2012) [11], Kreyling et al (2014) [13], and Kreyling et al (2018) [12], respectively, and then converted the organ mass fraction to the volume fraction out of the total body weight.

| **Table S3.** | Gold nanoparticle-specific parameters used in the PBPK model after various routes of administration in rats. | | | | | | | |
| --- | --- | --- | --- | --- | --- | --- | --- | --- |
| Parameter (unit) | | Routes | Liver | Spleen | Kidneys | Lungs | GI tract | Rest of body |
| *P* (unitless) | | All | 0.08 | 0.15 | 0.15 | 0.15 | 0.15 | 0.15 |
| *PAC* (unitless) | | IV | 0.001 | 0.08 (1.4 nm)  0.25 (5 nm)  0.25 (18 nm)  0.3 (80 nm)  0.3 (200 nm) | 0.001 | 0.001 | 0.001 | 0.001 |
|  |  | Oral | 0.001 | 0.001 | 0.001 | 0.001 | 0.001 | 0.001 |
|  |  | IT | 0.001 | 0.001 | 0.001 | 0.001 | 0.001 | 0.001 |
|  |  | IH | 0.001 | 0.001 | 0.001 | 0.001 | 0.001 | 0.001 |
| *K_max_* (1/h) ^b^ | | IV | 0.95 (1.4 nm)  113 (5 nm)  46.2 (18 nm)  99.5 (80 nm)  79.9 (200 nm) | 4.57 (1.4 nm)  78.3 (5 nm)  15.9 (18 nm)  100 (80 nm)  2.37 (200 nm) | 5.67 (1.4 nm)  0.46 (5 nm)  0.18 (18 nm)  0.76 (80 nm)  0.13 (200 nm) | 0.34 (1.4 nm)  0.19 (5 nm)  1.00 (18 nm)  2.46 (80 nm)  3.27 (200 nm) | 0.20 (1.4 nm)  0.50 (5 nm)  10 (18 nm)  10 (80 nm)  0.2 (200 nm) | 0.20 (1.4 nm)  2 (5 nm)  0.7 (18 nm)  0.1 (80 nm)  0.01 (200 nm) |
|  | | Oral | 16.58 (5 nm)  0.088 (18 nm)  0.088 (80 nm)  7.58 (200 nm) | 4.39 (5 nm)  9.09 (18 nm)  0.131 (80 nm)  3.865 (200 nm) | 13.51 (5 nm)  0.91 (18 nm)  3.16 (80 nm)  4.23 (200 nm) | 18.34 (5 nm)  0.44 (18 nm)  6.98 (80 nm)  1.88 (200 nm) | 1 | 0.1 (5 nm)  0.5 (18 nm)  1 (80 nm)  2 (200 nm) |
|  | | IT | 0.01 | 0.01 | 46.8 (1.4 nm)  0.05 (5 nm)  0.04 (18 nm)  4e-5 (80 nm)  0.009 (200 nm) | 49 (1.4 nm)  278 (5 nm)  4.92 (18 nm)  211 (80 nm)  211 (200 nm) | 1 | 0.01 |
|  | | IH | 0.07 | 1 | 0.07 | 300 | 0.1 | 1 |
| *K_exo_* (1/h) ^b^ | | IV | 0.01 (1.4 nm)  0.01 (5 nm)  0.11 (18 nm)  0.01 (80 nm)  0.027 (200 nm) | 0.01 (1.4 nm)  0.1 (5 nm)  0.008 (18 nm)  0.005 (80 nm)  0.193 (200 nm) | 0.009 (1.4 nm)  0.009 (5 nm)  7.18 (18 nm)  0.04 (80 nm)  0.32 (200 nm) | 0.012 (1.4 nm)  0.02 (5 nm)  0.07 (18 nm)  0.03 (80 nm)  0.03 (200 nm) | 0.03 (1.4 nm)  0.35 (5 nm)  0.01 (18 nm)  0.01 (80 nm)  1 (200 nm) | 0.05 (1.4 nm)  0.8 (5 nm)  0.01 (18 nm)  0.2 (80 nm)  0.5 (200 nm) |
|  | | Oral | 9.59 (5 nm)  1.84 (18 nm)  0.84 (80 nm)  4.07 (200 nm) | 0.89 (5 nm)  0.09 (18 nm)  1.77 (80 nm)  1.04 (200 nm) | 0.09 (5 nm)  0.44 (18 nm)  0.43 (80 nm)  0.82 (200 nm) | 6.21 (5 nm)  4.5 (18 nm)  1.04 (80 nm)  4.67 (200 nm) | 0.001 | 0.2 (5 nm)  1.5 (18 nm)  0.25 (80 nm)  0.2(200 nm) |
|  | | IT | 0.5 | 0.009 (1.4 nm)  0.13 (5 nm)  0.45 (18 nm)  0.46 (80 nm)  0.46 (200 nm) | 0.3 (1.4 nm)  0.07 (5 nm)  0.44 (18 nm)  0.46 (80 nm)  0.46 (200 nm) | 0.008 (1.4 nm)  0.002 (5 nm)  0.008 (18 nm)  0.008 (80 nm)  0.008 (200 nm) | 0.1 | 0.5 |
|  | | IH | 0.004 | 0.001 | 0.001 | 0.1 | 0.5 | 0.1 |
| *A_cap_* (μg/kg tissue) ^b^ | | IV | 2000 (1.4 nm)  4000 (5 nm)  1500 (18 nm)  2500 (80 nm)  2200 (200 nm) | 1000 (1.4 nm)  4000 (5 nm)  1500 (18 nm)  1500 (80 nm)  2200 (200 nm) | 700 (1.4 nm)  10 (5 nm)  10 (18 nm)  10 (80 nm)  10 (200 nm) | 100 (1.4 nm)  100 (5 nm)  10 (18 nm)  10 (80 nm)  30 (200 nm) | 100 (1.4 nm)  100 (5 nm)  10 (18 nm)  10 (80 nm)  10 (200 nm) | 100 (1.4 nm)  100 (5 nm)  10 (18 nm)  10 (80 nm)  1.5 (200 nm) |
|  | | Oral | 100 | 100 | 100 | 100 | 100 | 1.5 |
|  | | IT | 10 | 10 | 100 (1.4 nm)  10 (5 nm)  10 (18 nm)  10 (80 nm)  10 (200 nm) | 4000 (1.4 nm)  6500 (5 nm)  1200 (18 nm)  5000 (80 nm)  5000 (200 nm) | 10 | 1.5 |
|  | | IH | 100 | 100 | 100 | 5000 | 100 | 1.5 |
| *K_bile_/K_urine_* (L/h) or *K_feces_* (1/h) ^b^ | | IV | 8e-4 (1.4 nm)  8e-4 (5 nm)  8e-4 (18 nm)  0.01 (80 nm)  1e-4 (200 nm) | NA | 1.1e-3 (1.4 nm)  1.5e-5 (5 nm)  8.9e-6 (18 nm)  2.1e-6 (80 nm)  1.1e-5 (200 nm) | NA | 0.387 (1.4 nm)  0.373 (5 nm)  2.6e-7 (18 nm)  0.002 (80 nm)  0.265 (200 nm) | NA |
|  | | Oral | 0.05 (5 nm)  0.05 (18 nm)  0.2 (80 nm)  0.2 (200 nm) | NA | 0.001 (5 nm)  4e-4 (18 nm)  6.9e-4 (80 nm)  8.2e-4 (200 nm) | NA | 1.32 (5 nm)  1.32 (18 nm)  0.66 (80 nm)  0.53 (200 nm) | NA |
|  | | IT | 1e-4 (1.4 nm)  8e-4 (5 nm)  8e-4 (18 nm)  0.05 (80 nm)  0.05 (200 nm) | NA | 9.1e-4 (1.4 nm)  4.3e-5 (5 nm)  7.2e-6 (18 nm)  5.2e-6 (80 nm)  5.2e-6 (200 nm) | NA | 2.6e-4 | NA |
|  | | IH | 0.0003 | NA | 0.005 | NA | 0.05 | NA |
| *K_GIb_* (1/h) ^b^ | | Oral | NA | NA | NA | NA | 0.0004 (5 nm)  0.001 (18 nm)  0.0001 (80 nm)  0.00006 (200 nm) | NA |
|  | | IH | NA | NA | NA | NA | 0.0001 (IH) | NA |
| *K_pulPCtra_* or *K_uagi_/K_tragi_* (1/h) ^b^ | | IT | NA | NA | NA | 0.05 (1.4 nm)  0.05 (5 nm)  0.1 (18 nm)  0.01 (80 nm)  0.03 (200 nm) | 0.002+0.88×SqPulse(0, 0.6) (1.4 nm) ^b^  0.002+0.8×SqPulse(0, 0.6) (5 nm)  0.002+0.52×SqPulse(0, 0.6) (18 nm)  0.002+0.55×SqPulse(0, 0.6) (80 nm)  0.002+0.7×SqPulse(0, 0.6) (200 nm) | NA |
|  | | IH | NA | NA | NA | 0.003 | 0.002+0.2×SqPulse(0, 1) ^b^ | NA |

Please note that the parameters listed in the Table S3 were used as initial values for the subsequent model optimization via the Bayesian-MCMC analysis.

**Abbreviation:** *P*, tissue:blood distribution coefficient; *PAC*, permeability coefficient; *K_max_*, maximum endocytic uptake rate constant; *K*_exo_, exocytic rate constant; *A_cap_*, endocytosis uptake capacity; *K_bile_*, *K_urine_*, and *K_feces_*, biliary, urinary, and fecal excretion rate constants; *K_GIb_*, absorption rate constant from GI lumen to capillary blood; *K_pulPCtra_*, transport rate constant from inactive pulmonary endocytic cells to tracheobronchial region of lungs; *K_uagi_* and *K_tragi_*, clearance rate from upper airways and tracheobronchial regions to GI tract, respectively.

^a^ PACs in spleen were estimated as 0.008, 0.25, 0.25, 0.3, and 0.3 in rats intravenously injected with 1.4, 5, 18, 80, and 200 nm AuNPs, respectively.

^b^ Mucociliary clearance of AuNPs described by a SQUAREPULSE function in Berkeley Madonna as *K_slow_*+*K_fast_*×SQUAREPULSE(0, *t_D_*), where *K_slow_* is the baseline transport rate constant and *t_D_* is the duration for the fast-phase clearance rate constant (*K_fast_*).

| **Table S4** | Posterior uncertainty distributions for the population mean (µ) and variance ($\Sigma^{2}$) of the PBPK model parameters following oral administration. | | | | |
| --- | --- | --- | --- | --- | --- |
|  | | **Population Mean (µ)** |  | **Population variance (**$\boldsymbol{\Sigma}^{\boldsymbol{2}}$**)** |  |
| **Parameters** | | **Median (2.5%, 97.5%)** | $\hat{\mathbf{R}}$ | **Median (2.5%, 97.5%)** | $\hat{\mathbf{R}}$ |
| ***5 nm*** | |  |  |  |  |
| lnKLRESrelease | | 2.25 (1.44, 3.09) | 1.00 | 3.55 (0.59, 15.84) | 1.05 |
| lnKLRESmax | | 2.84 (1.95, 3.67) | 1.05 | -2.44 (-32.8, 4.04) | 1.03 |
| lnKSRESrelease | | 0.13 (-0.93, 0.85) | 1.05 | 0.98 (-4.88, 6.73) | 1.03 |
| lnKSRESmax | | 1.50 (0.70, 1.68) | 1.00 | 1.87 (-3.40, 7.50) | 1.03 |
| lnKKRESrelease | | -2.42 (-3.26, -1.60) | 1.01 | -3.02 (-9.01, 0.05) | 1.05 |
| lnKKRESmax | | 2.61 (1.79, 3.48) | 1.01 | 3.49 (0.75, 12.30) | 1.01 |
| lnKpulRESrelease | | 1.78 (0.99, 2.68) | 1.01 | 1.48 (-4.77, 8.55) | 1.01 |
| lnKpulRESmax | | 3.03 (2.01, 3.73) | 1.01 | 34.24 (-3.05, 128) | 1.02 |
| lnKurineC | | -6.89 (-7.62, -6.08) | 1.05 | -6.95 (0.52, -5.79) | 1.01 |
| ***18 nm*** | |  |  |  |  |
| lnKLRESrelease | | 0.66 (-0.26, 1.51) | 1.03 | 0.96 (-2.32, 6.16) | 1.00 |
| lnKLRESmax | | -1.38 (-3.29, 1.78) | 1.11 | -2.68 (-14.4, 1.24) | 1.12 |
| lnKSRESrelease | | -2.11 (-3.25, -1.26) | 1.67 | -4.03 (12.6, 1.98) | 1.05 |
| lnKSRESmax | | 1.82 (0.69, 3.03) | 1.04 | 1.90 (-2.35, 10.13) | 1.03 |
| lnKKRESrelease | | -0.83 (-1.68, 1.00) | 1.01 | -1.00 (7.95, 2.82) | 1.01 |
| lnKKRESmax | | 0.27 (-0.96, 1.17) | 1.71 | 0.13 () | 1.01 |
| lnKpulRESrelease | | 0.85 (-1.03, 2.29) | 1.28 | 1.65 | 1.01 |
| lnKpulRESmax | | -0.88 (-1.62, 0.03) | 1.17 | -1.10 | 1.01 |
| lnKurineC | | -6.15 (-8.38, -1.84) | 1.12 | -5.17 | 1.18 |
| ***80 nm*** | |  |  |  |  |
| lnKLRESrelease | | -0.09 (-1.01, 0.84) | 1.00 | 3.25 (-2.70, 48.6) | 1.19 |
| lnKLRESmax | | -2.43 (-3.36, -1.46) | 1.00 | -3.25 (-10.05, -0.35) | 1.01 |
| lnKSRESrelease | | 0.59 (-0.33, 1.52) | 1.00 | 0.38 (-10.5, 11.31) | 1.00 |
| lnKSRESmax | | -2.00 (-2.93, -1.09) | 1.00 | -2.50 (-11.3, 2.11) | 1.00 |
| lnKKRESrelease | | -0.84 (-1.75, 0.06) | 1.00 | -1.25 (-7.05, 2.18) | 1.00 |
| lnKKRESmax | | 1.17 (0.25, 2.05) | 1.00 | 1.20 (-2.67, 5.52) | 1.01 |
| lnKpulRESrelease | | 0.07 (-0.84, 1.02) | 1.00 | 0.04 (-7.17, 7.12) | 1.00 |
| lnKpulRESmax | | 1.97 (1.03, 2.31) | 1.00 | 2.17 (-9.26, 13.54) | 1.00 |
| lnKurineC | | -7.04 (-7.97, -6.10) | 1.00 | -5.66 (-7.33, 1.00) | 1.01 |
| ***200*** | |  |  |  |  |
| lnKLRESrelease | | 1.37 (0.44, 2.28) | 1.00 | 3.19 (-0.83, 17.73) | 1.01 |
| lnKLRESmax | | 2.12 (1.24, 3.02) | 1.00 | -0.50 (-19.49, 3.80) | 1.03 |
| lnKSRESrelease | | 0.09 (-0.85, 1.05) | 1.00 | 0.87 (-7.23, 12.79) | 1.04 |
| lnKSRESmax | | 1.28 (0.36, 2.19) | 1.00 | 1.51 (-5.64, 9.66) | 1.00 |
| lnKKRESrelease | | -0.09 (-1.03, 0.83) | 1.00 | 0.32 (-5.66, 9.21) | 1.01 |
| lnKKRESmax | | 1.48 (0.60, 2.42) | 1.00 | 1.62 (-5.49, 10.0) | 1.02 |
| lnKpulRESrelease | | -0.80 (-1.72, 0.10) | 1.00 | -1.21 (-9.03, 4.95) | 1.02 |
| lnKpulRESmax | | 2.86 (1.97, 3.79) | 1.00 | 2.62 (-10.2, 9.43) | 1.00 |
| lnKurineC | | -7.10 (-7.98, -6.20) | 1.00 | -7.06 (-8.83, -5.20) | 1.00 |

Abbreviations: KLRESrelease, KSRESrelease, KKRESrelease and KpulRESrelease represent the release rate constants of phagocytic cells in liver, spleen, kidney and lung, respectively; KLRESmax, KSRESmax, KKRESmax and KpulRESmax represent the maximum uptake rate constants in liver, spleen, kidney and lung, respectively; KurineC is the urinary elimination rate constant. All the parameter values were log-transformed.

Note: Only critical and sensitive parameters to the model were considered in the Bayesian analysis.

| **Table S5** | Posterior uncertainty distributions for the population mean (µ) and variance ($\Sigma^{2}$) of the PBPK model parameters following intravenous administration. | | | | |
| --- | --- | --- | --- | --- | --- |
|  | | **Population Mean (µ)** |  | **Population variance (**$\boldsymbol{\Sigma}^{\boldsymbol{2}}$**)** |  |
| **Parameters** | | **Median (2.5%, 97.5%)** | $\hat{\mathbf{R}}$ | **Median (2.5%, 97.5%)** | $\hat{\mathbf{R}}$ |
| ***1.4 nm*** | |  |  |  |  |
| lnKLRESrelease | | -4.70 (-5.65, -3.81) | 1.01 | -5.09 (-12.27, -1.64) | 1.02 |
| lnKLRESmax | | -0.07 (-1.15, 0.90) | 1.13 | 0.29 (-1.91, 3.98) | 1.01 |
| lnKSRESrelease | | -4.70 (-5.62, -3.82) | 1.04 | -5.33 (-11.83, -2.00) | 1.01 |
| lnKSRESmax | | 1.67 (0.80, 2.64) | 1.04 | 3.44 (0.01, 18.4) | 1.09 |
| lnKKRESrelease | | -4.70 (-5.80, -3.84) | 1.04 | -4.96 (-10.6, -1.91) | 1.02 |
| lnKKRESmax | | 1.79 (0.92, 2.76) | 1.01 | 2.41 (-0.18, 9.12) | 1.02 |
| lnKpulRESrelease | | -4.71 (-5.61, -3.80) | 1.00 | -4.88 (-12.1, 1.21) | 1.02 |
| lnKpulRESmax | | -1.29 (-2.20, -0.38) | 1.00 | -1.29 (-9.36, 6.51) | 1.00 |
| lnKurineC | | -7.07 (-7.88, -6.29) | 1.00 | -7.10 (-8.05, -6.22) | 1.00 |
| ***5 nm*** | |  |  |  |  |
| lnKLRESrelease | | -4.49 (-5.38, -3.50) | 1.03 | -5.59 (-15.4, -3.07) | 1.03 |
| lnKLRESmax | | 4.68 (3.84, 5.47) | 1.02 | 8.91 (4.19, 18.3) | 1.14 |
| lnKSRESrelease | | -2.17 (-3.00, -1.30) | 1.01 | -7.60 (-96.8, -1.01) | 1.20 |
| lnKSRESmax | | 4.19 (3.21, 4.99) | 1.01 | 4.34 (3.28, 5.98) | 1.03 |
| lnKKRESrelease | | -4.77 (-5.66, -3.84) | 1.31 | -5.48 (-12.6, -2.26) | 1.01 |
| lnKKRESmax | | -0.91 (-1.74, 0.05) | 1.01 | -0.82 (-3.22, 4.67) | 1.12 |
| lnKpulRESrelease | | -3.64 (-4.45, -2.79) | 1.01 | -4.01 (-12.6, 0.62) | 1.04 |
| lnKpulRESmax | | -1.72 (-2.57, -0.85) | 1.05 | -2.42 (-9.70, 3.62) | 1.04 |
| lnKurineC | | -11.0 (-11.8, -10.3) | 1.06 | -11.02 (-11.83, -10.33) | 1.09 |
| ***18 nm*** | |  |  |  |  |
| lnKLRESrelease | | -2.20 (-3.01, -1.29) | 1.00 | -2.54 (-3.52, -1.56) | 1.00 |
| lnKLRESmax | | 3.86 (3.09, 4.61) | 1.00 | 3.93 (3.40, 4.70) | 1.00 |
| lnKSRESrelease | | -4.74 (-5.66, -3.80) | 1.00 | -6.12 (-19.8, -3.11) | 1.00 |
| lnKSRESmax | | 2.88 (2.05, 3.69) | 1.00 | 3.11 (2.22, 4.36) | 1.02 |
| lnKKRESrelease | | -2.71 (-3.80, -1.80) | 1.00 | -3.94 (-14.5, 0.11) | 1.04 |
| lnKKRESmax | | -2.34 (-3.28, -1.44) | 1.00 | -2.29 (-5.57, -0.14) | 1.02 |
| lnKpulRESrelease | | -2.58 (-3.49, -1.65) | 1.00 | -2.38 (-12.5, 8.31) | 1.02 |
| lnKpulRESmax | | -0.01 (-0.91, 0.89) | 1.00 | 0.01 (-15.3, 20.2) | 1.00 |
| lnKurineC | | -11.5 (-12.3, -10.8) | 1.00 | -11.36 (-12.1, -10.6) | 1.01 |
| ***80 nm*** | |  |  |  |  |
| lnKLRESrelease | | -4.52 (-5.45, -3.60) | 1.02 | -3.09 (-5.02, 0.04) | 1.01 |
| lnKLRESmax | | 4.63 (3.78, 5.49) | 1.00 | 5.27 (4.13, 8.07) | 1.01 |
| lnKSRESrelease | | -5.41 (-6.31, -4.47) | 1.00 | -6.86 (-18.2, -2.58) | 1.01 |
| lnKSRESmax | | 4.52 (3.65, 5.47) | 1.00 | 5.39 (3.26, 15.20) | 1.03 |
| lnKKRESrelease | | -3.13 (-4.04, -2.16) | 1.00 | -2.89 (-11.5, 4.55) | 1.02 |
| lnKKRESmax | | -0.24 (-1.19, 0.66) | 1.00 | -1.66 (-9.79, 0.78) | 1.02 |
| lnKpulRESrelease | | -3.45 (-4.42, -2.52) | 1.00 | -3.64 (-11.45, 4.55) | 1.01 |
| lnKpulRESmax | | 0.98 (0.07, 1.90) | 1.00 | 0.46 (-5.80, 7.27) | 1.01 |
| lnKurineC | | -13.14 (-13.9, -12.3) | 1.01 | -13.36 (-14.4, -12.4) | 1.01 |
| ***200 nm*** | |  |  |  |  |
| lnKLRESrelease | | -1.40 (-4.66, 1.51) | 1.06 | -1.91 (-4.69, 0.77) | 1.20 |
| lnKLRESmax | | 2.78 (0.33, 5.40) | 1.20 | 2.70 (-0.77, 7.12) | 1.19 |
| lnKSRESrelease | | -1.38 (-2.75, -0.74) | 1.17 | -1.57 (-5.50, 0.56) | 1.16 |
| lnKSRESmax | | 2.72 (-1.83, 6.55) | 1.07 | 3.83 (0.10, 11.10) | 1.11 |
| lnKKRESrelease | | -0.38 (-1.58, 1.19) | 1.14 | -0.63 (-4.71, 5.74) | 1.03 |
| lnKKRESmax | | -1.22 (-3.23, 0.65) | 1.14 | -1.37 (-10.1, 1.89) | 1.12 |
| lnKpulRESrelease | | -2.1 (-4.41, 0.50) | 1.12 | -2.18 (-8.06, 0.42) | 1.14 |
| lnKpulRESmax | | 0.99 (-0.73, 2.27) | 1.16 | 0.87 (-3.12, 6.01) | 1.04 |
| lnKurineC | | -5.99 (-0.73, 2.27) | 1.12 | -5.76 (-12.9, 1.09) | 1.18 |

Abbreviation: KLRESrelease, KSRESrelease, KKRESrelease and KpulRESrelease represent the release rate constants of phagocytic cells in liver, spleen, kidney and lung, respectively; KLRESmax, KSRESmax, KKRESmax and KpulRESmax represent the maximum uptake rate constants in liver, spleen, kidney and lung, respectively; KurineC is the urinary elimination constant rate. All the parameter values were log-transformed.

Note: Only critical and sensitive parameters to the model were considered in the Bayesian analysis.

| **Table S6** | Posterior uncertainty distributions for the population mean (µ) and variance ($\Sigma^{2}$) of the PBPK model parameters following intratracheal instillation. | | | | |
| --- | --- | --- | --- | --- | --- |
|  | | **Population Mean (µ)** |  | **Population variance (**$\boldsymbol{\Sigma}^{\boldsymbol{2}}$**)** |  |
| **Parameters** | | **Median (2.5%, 97.5%)** | $\hat{\mathbf{R}}$ | **Median (2.5%, 97.5%)** | $\hat{\mathbf{R}}$ |
| **1.4 nm** | |  |  |  |  |
| lnKLRESrelease | | -0.88 ( -1.76, 0.06) | 1.02 | -1.32 (-9.53, 7.57) | 1.03 |
| lnKLRESmax | | -4.85 ( -5.56, -3.92) | 1.11 | -6.14 (-21.2, -2.3) | 1.02 |
| lnKSRESrelease | | -4.57( -5.60, -3.87) | 1.19 | -3.43 (-11.6, 21.2) | 1.07 |
| lnKSRESmax | | -4.74 ( -5.55, -3.85) | 1.01 | -2.62 (-12.0, 5.78) | 1.04 |
| lnKKRESrelease | | -1.25 ( -2.06, -0.34) | 1.10 | -1.85 (-7.68, 0.65) | 1.27 |
| lnKKRESmax | | 3.73 (2.96, 4.73) | 1.15 | 4.52 (2.07, 13.16) | 1.02 |
| lnKpulRESrelease | | -4.84 ( -5.55, -3.88) | 1.04 | -5.36 (-12.1, -0.40) | 1.12 |
| lnKpulRESmax | | 4.00 ( 3.05, 4.83) | 1.03 | -0.45 (-2.47, 4.01) | 1.00 |
| lnKurineC | | -6.99 ( -7.91, -6.17) | 1.02 | -5.54 (-6.77, -4.53) | 1.03 |
| ***5 nm*** | |  |  |  |  |
| lnKLRESrelease | | -0.84 (-1.35, -0.30) | 1.38 | -2.19 (-3.50, 0.92) | 1.02 |
| lnKLRESmax | | -4.29 (-5.18, -3.84) | 1.74 | -4.50 (-7.90, -2.23) | 1.08 |
| lnKSRESrelease | | -2.04 (-2.77, -1.38) | 1.76 | -2.52 (-3.86, -0.52) | 1.13 |
| lnKSRESmax | | -4.81 (-5.67, -4.16) | 1.72 | -5.12 (-32.74, -0.95) | 1.19 |
| lnKKRESrelease | | -2.45 (-3.50, -2.08) | 1.95 | -3.46 (-5.43, -1.66) | 1.33 |
| lnKKRESmax | | -3.57 (-4.30, -2.27) | 1.58 | -3.11 (-4.72, -0.97) | 1.29 |
| lnKpulRESrelease | | -6.24 (-6.84, -5.79) | 1.37 | -5.75 (-8.69, -1.17) | 1.10 |
| lnKpulRESmax | | 5.99 (5.10, 6.68) | 1.41 | 10.44 (4.51, 13.12) | 1.14 |
| lnKurineC | | -10.05 (-10.87, -9.20) | 1.20 | -10.09 (-11.20, -8.65) | 1.01 |
| ***18 nm*** | |  |  |  |  |
| lnKLRESrelease | | -0.77 (-1.68, 0.24) | 1.01 | 0.83 (-8.06, 30.95) | 1.00 |
| lnKLRESmax | | -4.72 (-5.63, -3.82) | 1.02 | -5.23 (-12.78, -1.73) | 1.04 |
| lnKSRESrelease | | -0.80 (-1.72, 0.10) | 1.00 | -1.13 (-10.9, 6.92) | 1.01 |
| lnKSRESmax | | -4.70 (-5.62, -5.62) | 1.00 | -4.92 (-12.1, 0.44) | 1.03 |
| lnKKRESrelease | | -0.82 (-1.67, 0.11) | 1.00 | -0.83 (-16.9, 9.47) | 1.00 |
| lnKKRESmax | | -3.12 (-4.03, -2.22) | 1.00 | -3.73 (-13.5, 0.14) | 1.01 |
| lnKpulRESrelease | | -4.72 (-5.03, -3.80) | 1.00 | -6.09 (-16.4, -2.67) | 1.00 |
| lnKpulRESmax | | 1.63 (0.72, 2.58) | 1.00 | 4.52 (3.81, 5.26) | 1.01 |
| lnKurineC | | -11.86 (-12.7, -10.9) | 1.00 | -8.33 (-9.98, -6.81) | 1.00 |
| ***80 nm*** | |  |  |  |  |
| lnKLRESrelease | | -1.03 (-1.80, 0.08) | 1.46 | -0.94 (-8.09, 4.68) | 1.00 |
| lnKLRESmax | | -4.76 (-5.55, -3.74) | 1.28 | -5.20 (-9.63, -2.30) | 1.01 |
| lnKSRESrelease | | -0.80 (-1.61, 0.08) | 1.24 | -1.67 (-9.89, 6.80) | 1.03 |
| lnKSRESmax | | -4.70 (-5.53, -3.86) | 1.10 | -10.11 (-25.3, 0.01) | 1.03 |
| lnKKRESrelease | | -1.14 (-2.20, 0.04) | 1.02 | -1.33 (-7.21, 3.96) | 1.00 |
| lnKKRESmax | | -4.78 (-5.58, -3.91) | 1.01 | -5.13 (-11.8, -1.79) | 1.04 |
| lnKpulRESrelease | | -4.71 (-5.62, -3.84) | 1.02 | -5.38 (-12.2, -3.06) | 1.04 |
| lnKpulRESmax | | 5.26 (4.52, 6.21) | 1.05 | 7.23 (3.17, 13.28) | 1.01 |
| lnKurineC | | -12.21 (-13.07, -11.34) | 1.00 | -8.29 (-10.8, -4.87) | 1.03 |
| ***200 nm*** | |  |  |  |  |
| lnKLRESrelease | | -0.78 (-1.70, 0.15) | 102 | -0.57 (-10.71, 9.38) | 1.00 |
| lnKLRESmax | | -4.75 (-5.84, -3.82) | 1.01 | -5.57 ( -14.5, -1.28) | 1.00 |
| lnKSRESrelease | | -0.80 (-1.75, 0.09) | 1.01 | -0.83 (-9.25, 6.26) | 1.02 |
| lnKSRESmax | | -4.75 (-5.67, -3.79) | 1.01 | -2.61 (-15.4, 31.4) | 1.00 |
| lnKKRESrelease | | -1.24 (-2.24, -0.32) | 1.00 | -0.63 (-9.86, 10.8) | 1.14 |
| lnKKRESmax | | -4.74 (-5.59, -3.83) | 1.06 | -4.99 (-11.24, -0.82) | 1.01 |
| lnKpulRESrelease | | -3.69 (-4.53, -2.79) | 1.00 | -4.44 (-11.97, -1.76) | 1.00 |
| lnKpulRESmax | | 1.63 (0.64, 2.56) | 1.01 | 6.27 (4.60, 8.00) | 1.00 |
| lnKurineC | | -12.91 (-13.8, -12.01) | 1.04 | -7.88 (-11.8, -4.88) | 1.00 |

Abbreviation: KLRESrelease, KSRESrelease, KKRESrelease and KpulRESrelease represent the release rate constants of phagocytic cells in liver, spleen, kidney and lung, respectively; KLRESmax, KSRESmax, KKRESmax and KpulRESmax represent the maximum uptake rate constants in liver, spleen, kidney and lung, respectively; KurineC is the urinary elimination constant rate. All the parameter values were log-transformed.

Note: Only critical and sensitive parameters to the model were considered in the Bayesian analysis.

| **Table S7** | Posterior uncertainty distributions for the population mean (µ) and variance ($\Sigma^{2}$) of the PBPK model parameters following endotracheal inhalation for 23 nm AuNPs. | | | | |
| --- | --- | --- | --- | --- | --- |
|  | | **Population Geometric Mean (µ)** |  | **Population Geometric Standard Deviation (**$\boldsymbol{\Sigma}^{\boldsymbol{2}}$**)** |  |
| **Parameters** | | **Median (2.5%, 97.5%)** | $\hat{\mathbf{R}}$ | **Median (2.5%, 97.5%)** | $\hat{\mathbf{R}}$ |
| **23 nm** | |  |  |  |  |
| lnKLRESrelease | | -0.28 (-1.38, 0.72) | 1.01 | 0.86 (-0.44, 1.54) | 1.01 |
| lnKLRESmax | | 1.28 (0.26, 2.09) | 1.01 | 1.39 (1.13, 1.86) | 1.01 |
| lnKSRESrelease | | 0.03 (-0.91, 1.29) | 1.01 | -0.07 (-1.92, 1.55) | 1.01 |
| lnKSRESmax | | -0.44 (-1.86, 0.62) | 1.01 | -0.48 (-1.11, 0.35) | 1.01 |
| lnKKRESrelease | | 0.17 (-1.65, 1.37) | 1.01 | 0.46 (-1.96, 1.54) | 1.01 |
| lnKKRESmax | | 0.25 (-0.11, 1.45) | 1.01 | 0.54 (-1.25, 1.99) | 1.01 |
| lnKpulRESrelease | | -0.01 (-1.92, 1.30) | 1.01 | 0.27 ( -0.17, 1.42) | 1.01 |
| lnKpulRESmax | | 0.40 (-0.59, 1.23) | 1.01 | 0.51 (-1.01, 1.18) | 1.01 |
| lnKurineC | | 0.43 (-1.09, 1.26) | 1.01 | -0.55 (-1.99, 0.79) | 1.01 |

Abbreviation: KLRESrelease, KSRESrelease, KKRESrelease and KpulRESrelease represent the release rate constant of phagocytic cells in liver, spleen, kidney and lung, respectively; KLRESmax, KSRESmax, KKRESmax and KpulRESmax represent the maximum uptake rate constants in liver, spleen, kidney and lung, respectively; KurineC is the urinary elimination constant rate. All the parameter values were log-transformed.

Note: Only critical and sensitive parameters to the model were considered in the Bayesian analysis.

| **Table S8** | Normalized sensitivity coefficients (NSCs) of highly influential parameters after oral administration to 5 nm AuNPs. | | | | | | | | | | | | | | |
| --- | --- | --- | --- | --- | --- | --- | --- | --- | --- | --- | --- | --- | --- | --- | --- |
|  | Short term (24 hours) | | | | | | |  | Long term (672 hours) | | | | | | |
|  | AUCB | AUCLu | AUCGI | AUCS | AUCRt | AUCL | AUCK |  | AUCB | AUCLu | AUCGI | AUCS | AUCRt | AUCL | AUCK |
| **Maximum endocytosis/phagocytosis uptake rate** | | | | | | | |  |  |  |  |  |  |  |  |
| KGIRESmax | -0.01 | -0.01 | 0.00 | -0.01 | -0.01 | -0.01 | 0.00 |  | -0.05 | -0.05 | 0.01 | -0.04 | -0.05 | -0.05 | -0.05 |
| KPulRESmax | 0.00 | 0.00 | 0.00 | 0.00 | 0.00 | 0.00 | 0.00 |  | 0.00 | 0.00 | 0.00 | 0.00 | 0.00 | 0.00 | 0.00 |
| KKRESmax | -0.02 | -0.02 | 0.00 | -0.01 | -0.02 | -0.02 | **0.23** |  | -0.01 | -0.01 | 0.00 | -0.03 | -0.01 | -0.01 | **0.95** |
| KLRESmax | **-0.16** | **-0.16** | 0.00 | **-0.19** | **-0.18** | **0.46** | **-0.19** |  | 0.00 | 0.00 | 0.00 | -0.01 | 0.00 | **0.62** | 0.00 |
| KSRESmax | 0.00 | 0.00 | 0.00 | 0.05 | 0.00 | 0.00 | 0.00 |  | -0.01 | -0.01 | 0.00 | **0.69** | -0.01 | -0.01 | -0.01 |
| KRRESmax | -0.04 | -0.04 | 0.00 | -0.03 | 0.08 | -0.04 | -0.02 |  | 0.00 | 0.00 | 0.00 | -0.01 | **0.28** | 0.00 | 0.00 |
| **Endocytosis/phagocytosis uptake capacity** | | | | | | | |  |  |  |  |  |  |  |  |
| AGIREScap | 0.00 | 0.00 | 0.00 | 0.00 | 0.00 | 0.00 | 0.00 |  | 0.00 | 0.00 | 0.00 | 0.00 | 0.00 | 0.00 | 0.00 |
| APulREScap | 0.00 | 0.00 | 0.00 | 0.00 | 0.00 | 0.00 | 0.00 |  | 0.00 | 0.00 | 0.00 | 0.00 | 0.00 | 0.00 | 0.00 |
| AKREScap | 0.00 | 0.00 | 0.00 | 0.00 | 0.00 | 0.00 | 0.00 |  | 0.00 | 0.00 | 0.00 | 0.00 | 0.00 | 0.00 | 0.01 |
| ALREScap | 0.00 | 0.00 | 0.00 | 0.00 | 0.00 | 0.00 | 0.00 |  | 0.00 | 0.00 | 0.00 | 0.00 | 0.00 | 0.00 | 0.00 |
| ASREScap | 0.00 | 0.00 | 0.00 | 0.00 | 0.00 | 0.00 | 0.00 |  | 0.00 | 0.00 | 0.00 | 0.00 | 0.00 | 0.00 | 0.00 |
| ARREScap | 0.00 | 0.00 | 0.00 | 0.00 | 0.00 | 0.00 | 0.00 |  | 0.00 | 0.00 | 0.00 | 0.00 | 0.00 | 0.00 | 0.00 |
| **Release rate constant of phagocytic cells** | | | | | | | |  |  |  |  |  |  |  |  |
| KGIRESrelease | 0.00 | 0.00 | 0.00 | 0.00 | 0.00 | 0.00 | 0.00 |  | 0.02 | 0.02 | 0.00 | 0.01 | 0.02 | 0.02 | 0.02 |
| KPulRESrelease | 0.00 | 0.00 | 0.00 | 0.00 | 0.00 | 0.00 | 0.00 |  | 0.00 | 0.00 | 0.00 | 0.00 | 0.00 | 0.00 | 0.00 |
| KKRESrelease | 0.01 | 0.01 | 0.00 | 0.01 | 0.01 | 0.01 | **-0.11** |  | 0.01 | 0.01 | 0.00 | 0.03 | 0.01 | 0.01 | **-0.94** |
| KLRESrelease | **0.16** | **0.16** | 0.00 | **0.18** | **0.18** | **-0.46** | **0.19** |  | 0.00 | 0.00 | 0.00 | 0.01 | 0.00 | **-0.62** | 0.00 |
| KSRESrelease | 0.00 | 0.00 | 0.00 | -0.02 | 0.00 | 0.00 | 0.00 |  | 0.01 | 0.01 | 0.00 | **-0.68** | 0.01 | 0.01 | 0.01 |
| KRRESrelease | 0.02 | 0.02 | 0.00 | 0.02 | -0.05 | 0.02 | 0.01 |  | 0.00 | 0.00 | 0.00 | 0.01 | **-0.28** | 0.00 | 0.00 |
| **Urinary, fecal and biliary excretion constant rate** | | | | | | | |  |  |  |  |  |  |  |  |
| KurineC | -0.05 | -0.05 | 0.00 | -0.04 | -0.04 | -0.05 | -0.03 |  | **-0.24** | **-0.24** | -0.01 | **-0.22** | **-0.24** | **-0.24** | **-0.24** |
| KfecesC | **-0.93** | **-0.93** | **-0.99** | **-0.87** | **-0.89** | **-0.93** | **-0.86** |  | **-0.99** | **-0.99** | **-0.99** | **-0.99** | **-0.99** | **-0.99** | **-0.98** |
| KbileC | -0.01 | -0.01 | 0.00 | 0.00 | 0.00 | -0.01 | 0.00 |  | -0.03 | -0.03 | 0.00 | -0.03 | -0.03 | -0.04 | -0.03 |

AUCB, AUCLu, AUCGI, AUCS, AUCRt, AUCL, and AUCK represent area-under-the-concentration curves of gold nanoparticles (AuNPs) in artery blood, lungs, gastrointestinal (GI) tract, spleen, rest of body, liver, and kidneys, respectively. K_max_ is maximum uptake rate constant for endocytosis/phagocytosis; K_release_ is the release rate constant of phagocytic cells; A_cap_ is endocytosis/phagocytosis uptake capacity. GI, Pul, K, L, R, and S stand for the GI tract, Pulmonary, Kidneys, Livers, Rest of body and Spleen, respectively. The absolute values of NSC large or equal to 0.1 were highlighted in bold. Parameter value of around or greater than 0.5 were considered highly sensitive.

| **Table S9.** | Normalized sensitivity coefficients (NSCs) of highly influential parameters after intravenous (IV) administration to 5 nm AuNPs. | | | | | | | | | | | | | | |
| --- | --- | --- | --- | --- | --- | --- | --- | --- | --- | --- | --- | --- | --- | --- | --- |
|  | Short term (24 hours) | | | | | | |  | Long term (672 hours) | | | | | | |
|  | AUCB | AUCLu | AUCGI | AUCS | AUCRt | AUCL | AUCK |  | AUCB | AUCLu | AUCGI | AUCS | AUCRt | AUCL | AUCK |
| **Maximum endocytosis/phagocytosis uptake rate** | | | | | | | |  |  |  |  |  |  |  |  |
| KGIRESmax | -0.01 | -0.01 | **0.23** | -0.01 | -0.01 | 0.00 | -0.01 |  | 0.00 | 0.00 | 0.48 | 0.00 | 0.00 | 0.00 | 0.00 |
| KPulRESmax | 0.00 | 0.00 | 0.00 | 0.00 | 0.00 | 0.00 | 0.00 |  | 0.00 | 0.00 | 0.00 | 0.00 | 0.00 | 0.00 | 0.00 |
| KKRESmax | 0.00 | 0.00 | 0.00 | 0.00 | 0.00 | 0.00 | **0.52** |  | 0.00 | 0.00 | 0.00 | 0.00 | 0.00 | 0.00 | **0.62** |
| KLRESmax | **-0.56** | **-0.55** | **-0.55** | **-0.47** | **-0.52** | 0.02 | **-0.46** |  | **-0.78** | **-0.78** | **-0.78** | **-0.42** | **-0.76** | 0.04 | **-0.55** |
| KSRESmax | -0.01 | -0.01 | -0.01 | **0.33** | -0.01 | -0.01 | -0.01 |  | -0.04 | -0.04 | -0.04 | 0.43 | -0.04 | -0.01 | -0.03 |
| KRRESmax | 0.00 | 0.00 | 0.00 | 0.00 | 0.07 | 0.00 | 0.00 |  | -0.04 | -0.04 | -0.04 | -0.02 | **0.33** | -0.01 | -0.03 |
| **Endocytosis/phagocytosis uptake capacity** | | | | | | | |  |  |  |  |  |  |  |  |
| AGIREScap | 0.00 | 0.00 | 0.00 | 0.00 | 0.00 | 0.00 | 0.00 |  | 0.00 | 0.00 | 0.00 | 0.00 | 0.00 | 0.00 | 0.00 |
| APulREScap | 0.00 | 0.00 | 0.00 | 0.00 | 0.00 | 0.00 | 0.00 |  | 0.00 | 0.00 | 0.00 | 0.00 | 0.00 | 0.00 | 0.00 |
| AKREScap | 0.00 | 0.00 | 0.00 | 0.00 | 0.00 | 0.00 | 0.13 |  | 0.00 | 0.00 | 0.00 | 0.00 | 0.00 | 0.00 | 0.29 |
| ALREScap | **-1.11** | **-1.02** | **-0.99** | **-0.79** | **-0.89** | 0.03 | **-0.82** |  | **-2.15** | **-2.15** | **-2.16** | **-1.17** | **-2.08** | 0.11 | **-1.53** |
| ARREScap | 0.00 | 0.00 | 0.00 | 0.00 | 0.00 | 0.00 | 0.00 |  | 0.00 | 0.00 | 0.00 | 0.00 | 0.01 | 0.00 | 0.00 |
| ASREScap | 0.00 | 0.00 | 0.00 | 0.09 | 0.00 | 0.00 | 0.00 |  | -0.04 | -0.04 | -0.04 | **0.43** | -0.03 | -0.01 | -0.03 |
| **Release rate constant of phagocytic cells** | | | | | | | |  |  |  |  |  |  |  |  |
| KGIRESrelease | 0.00 | 0.00 | **-0.20** | 0.00 | 0.00 | 0.00 | 0.00 |  | 0.00 | 0.00 | **-0.47** | 0.00 | 0.00 | 0.00 | 0.00 |
| KPulRESrelease | 0.00 | 0.00 | 0.00 | 0.00 | 0.00 | 0.00 | 0.00 |  | 0.00 | 0.00 | 0.00 | 0.00 | 0.00 | 0.00 | 0.00 |
| KKRESrelease | 0.00 | 0.00 | 0.00 | 0.00 | 0.00 | 0.00 | -0.07 |  | 0.00 | 0.00 | 0.00 | 0.00 | 0.00 | 0.00 | **-0.55** |
| KLRESrelease | **0.21** | **0.18** | **0.15** | **0.11** | **0.13** | 0.00 | **0.13** |  | **0.75** | **0.74** | **0.75** | **0.39** | **0.70** | -0.04 | **0.52** |
| KSRESrelease | 0.00 | 0.00 | 0.00 | -0.04 | 0.00 | 0.00 | 0.00 |  | 0.03 | 0.03 | 0.03 | **-0.37** | 0.03 | 0.01 | 0.02 |
| KRRESrelease | 0.00 | 0.00 | 0.00 | 0.00 | -0.01 | 0.00 | 0.00 |  | 0.04 | 0.04 | 0.04 | 0.02 | **-0.29** | 0.01 | 0.02 |
| **Urinary, fecal and biliary excretion constant rate** | | | | | | | |  |  |  |  |  |  |  |  |
| KurineC | 0.00 | 0.00 | 0.00 | 0.00 | 0.00 | 0.00 | 0.00 |  | 0.00 | 0.00 | 0.00 | 0.00 | 0.00 | 0.00 | 0.00 |
| KfecesC | 0.00 | 0.00 | -0.04 | 0.00 | 0.00 | 0.00 | 0.00 |  | 0.00 | 0.00 | -0.07 | 0.00 | 0.00 | 0.00 | 0.00 |
| KbileC | 0.00 | 0.00 | 0.04 | 0.00 | 0.00 | 0.00 | 0.00 |  | 0.00 | 0.00 | 0.05 | 0.00 | 0.00 | 0.00 | 0.00 |

AUCB, AUCLu, AUCGI, AUCS, AUCRt, AUCL, and AUCK represent area-under-the-concentration curves of gold nanoparticles (AuNPs) in artery blood, lungs, gastrointestinal (GI) tract, spleen, rest of body, liver, and kidneys, respectively. K_max_ is maximum uptake rate constant for endocytosis/phagocytosis; K_release_ is the release rate constant of phagocytic cells; A_cap_ is endocytosis/phagocytosis uptake capacity. GI, Pul, K, L, R, and S stand for the GI tract, Pulmonary, Kidneys, Livers, Rest of body and Spleen, respectively. The absolute values of NSC large or equal to 0.1 were highlighted in bold. Parameter value of around or greater than 0.5 were considered highly sensitive.

| **Table S10** | Normalized sensitivity coefficients (NSCs) of highly influential parameters after intratracheal instillation to 5 nm AuNPs. | | | | | | | | | | | | | | |
| --- | --- | --- | --- | --- | --- | --- | --- | --- | --- | --- | --- | --- | --- | --- | --- |
|  | Short term (24 hours) | | | | | | |  | Long term (672 hours) | | | | | | |
|  | AUCB | AUCLu | AUCGI | AUCS | AUCRt | AUCL | AUCK |  | AUCB | AUCLu | AUCGI | AUCS | AUCRt | AUCL | AUCK |
| **Maximum endocytosis/phagocytosis uptake rate** | | | | | | | |  |  |  |  |  |  |  |  |
| KGIRESmax | **0.24** | **-0.11** | **0.35** | **0.24** | **0.22** | **0.24** | **0.23** |  | 0.02 | -0.09 | 0.03 | 0.02 | 0.02 | 0.02 | 0.02 |
| KPulRESmax | 0.09 | **-0.12** | **0.38** | 0.09 | 0.05 | 0.08 | 0.08 |  | 0.03 | **-0.10** | 0.03 | 0.03 | 0.03 | 0.03 | 0.03 |
| KKRESmax | 0.00 | 0.00 | 0.00 | 0.00 | 0.00 | 0.00 | 0.11 |  | 0.00 | 0.00 | 0.00 | 0.00 | 0.00 | 0.00 | 0.21 |
| KLRESmax | -0.07 | 0.03 | -0.10 | -0.07 | -0.07 | -0.06 | -0.07 |  | -0.01 | 0.03 | -0.01 | -0.01 | -0.01 | 0.01 | -0.01 |
| KSRESmax | **0.21** | **-0.09** | **0.30** | **0.23** | **0.20** | **0.21** | **0.21** |  | 0.03 | -0.08 | 0.02 | 0.05 | 0.03 | 0.03 | 0.03 |
| KRRESmax | **0.24** | **-0.11** | **0.35** | **0.24** | **0.22** | **0.24** | **0.23** |  | 0.02 | -0.09 | 0.03 | 0.02 | 0.02 | 0.02 | 0.02 |
| **Endocytosis/phagocytosis uptake capacity** | | | | | | | |  |  |  |  |  |  |  |  |
| AGIREScap | -0.01 | 0.00 | -0.01 | -0.01 | 0.00 | -0.01 | -0.01 |  | 0.00 | 0.00 | 0.00 | 0.00 | 0.00 | 0.00 | 0.00 |
| APulREScap | **-7.14** | -0.09 | **0.30** | **-7.17** | **-8.27** | **-7.26** | **-7.46** |  | **-0.13** | **-0.21** | 0.07 | **-0.13** | **-0.13** | **-0.13** | **-0.13** |
| AKREScap | -0.01 | 0.00 | -0.01 | -0.01 | -0.01 | -0.01 | -0.01 |  | 0.00 | 0.00 | 0.00 | 0.00 | 0.00 | 0.00 | 0.00 |
| ALREScap | **0.26** | **-0.11** | **0.37** | **0.26** | **0.25** | **0.26** | **0.26** |  | 0.03 | -0.10 | 0.03 | 0.03 | 0.03 | 0.03 | 0.03 |
| ASREScap | 0.00 | 0.00 | -0.01 | 0.00 | 0.00 | 0.00 | 0.00 |  | 0.00 | 0.00 | 0.00 | 0.00 | 0.00 | 0.00 | 0.00 |
| ARREScap | 0.00 | 0.00 | 0.00 | 0.00 | 0.00 | 0.00 | 0.00 |  | 0.00 | 0.00 | 0.00 | 0.00 | 0.00 | 0.00 | 0.00 |
| **Release rate constant of phagocytic cells** | | | | | | | |  |  |  |  |  |  |  |  |
| KGIRESrelease | -0.01 | 0.00 | -0.01 | -0.01 | -0.01 | -0.01 | -0.01 |  | 0.01 | 0.01 | 0.00 | 0.01 | 0.01 | 0.01 | 0.01 |
| KPulRESrelease | -0.01 | 0.01 | -0.03 | -0.01 | -0.01 | -0.01 | -0.01 |  | 0.00 | 0.01 | 0.00 | 0.00 | 0.00 | 0.00 | 0.00 |
| KKRESrelease | **0.25** | **-0.11** | **0.35** | **0.25** | **0.23** | **0.25** | **0.20** |  | 0.03 | -0.09 | 0.03 | 0.03 | 0.03 | 0.03 | -0.17 |
| KLRESrelease | 0.01 | 0.00 | 0.01 | 0.01 | 0.01 | -0.01 | 0.01 |  | 0.00 | 0.00 | 0.00 | 0.00 | 0.00 | -0.02 | 0.00 |
| KSRESrelease | **0.28** | **-0.12** | **0.40** | **0.27** | **0.26** | **0.28** | **0.28** |  | 0.04 | -0.10 | 0.03 | 0.01 | 0.04 | 0.04 | 0.04 |
| KRRESrelease | 0.02 | -0.01 | 0.03 | 0.02 | 0.01 | 0.02 | 0.02 |  | 0.00 | -0.01 | 0.00 | 0.00 | -0.01 | 0.00 | 0.00 |
| **Urinary, fecal and biliary excretion constant rate** | | | | | | | |  |  |  |  |  |  |  |  |
| KurineC | **0.22** | -0.10 | **0.31** | **0.22** | **0.21** | **0.22** | **0.22** |  | 0.02 | -0.08 | 0.03 | 0.02 | 0.03 | 0.02 | 0.03 |
| KfecesC | 0.00 | 0.00 | 0.00 | 0.00 | 0.00 | 0.00 | 0.00 |  | -0.03 | 0.00 | -0.03 | -0.03 | -0.03 | -0.03 | -0.03 |
| KbileC | 0.01 | -0.01 | 0.02 | 0.01 | 0.01 | -0.01 | 0.01 |  | -0.02 | -0.01 | 0.00 | -0.02 | -0.02 | -0.05 | -0.02 |

AUCB, AUCLu, AUCGI, AUCS, AUCRt, AUCL, and AUCK represent area-under-the-concentration curve of gold nanoparticles (AuNPs) in artery blood, lungs, gastrointestinal (GI) tract, spleen, rest of body, liver, and kidneys, respectively. K_max_ is maximum uptake rate for endocytosis/phagocytosis; K_release_ is the Release rate constant of phagocytic cells; A_cap_ is endocytosis/phagocytosis uptake capacity. GI, Pul, K, L, R, and S indicated the GI tract, Pulmonary, Kidneys, Livers, Rest of body and Spleen, respectively. The absolute values of NSC large or equal to 0.1 was highlighted in bold. Parameter value of around or greater than 0.5 were considered highly sensitive.

| **Table S11** | Normalized sensitivity coefficients (NSCs) of highly influential parameters after inhalation administration to 23 nm AuNPs. | | | | | | | | | | | | | | |
| --- | --- | --- | --- | --- | --- | --- | --- | --- | --- | --- | --- | --- | --- | --- | --- |
|  | Short term (24 hours) | | | | | | |  | Long term (672 hours) | | | | | | |
|  | AUCB | AUCLu | AUCGI | AUCS | AUCRt | AUCL | AUCK |  | AUCB | AUCLu | AUCGI | AUCS | AUCRt | AUCL | AUCK |
| **Maximum endocytosis/phagocytosis uptake rate** | | | | | | | |  |  |  |  |  |  |  |  |
| KGIRESmax | 0.00 | 0.00 | 0.00 | 0.00 | 0.00 | 0.00 | 0.00 |  | 0.00 | 0.00 | 0.00 | 0.00 | 0.00 | 0.00 | 0.00 |
| KPulRESmax | **-0.20** | **0.17** | **-0.71** | **-0.25** | **-0.26** | **-0.24** | **-0.22** |  | **0.76** | 0.09 | -0.01 | **0.72** | **0.74** | **0.74** | **0.72** |
| KKRESmax | **-0.50** | 0.18 | **-0.74** | **-0.51** | **-0.40** | **-0.51** | **-0.50** |  | **-0.14** | **0.10** | -0.01 | **-0.16** | **0.58** | **-0.15** | **-0.16** |
| KLRESmax | -0.06 | 0.00 | 0.00 | -0.06 | -0.05 | **0.32** | -0.06 |  | -0.09 | 0.00 | 0.00 | -0.09 | -0.09 | **0.81** | -0.09 |
| KSRESmax | 0.00 | 0.00 | 0.00 | 0.15 | 0.00 | 0.00 | 0.00 |  | 0.00 | 0.00 | 0.00 | **0.31** | 0.00 | 0.00 | 0.00 |
| KRRESmax | 0.00 | 0.00 | 0.00 | 0.00 | 0.00 | 0.00 | 0.23 |  | 0.00 | 0.00 | 0.00 | 0.00 | 0.00 | 0.00 | **0.87** |
| **Endocytosis/phagocytosis uptake capacity** | | | | | | | |  |  |  |  |  |  |  |  |
| AGIREScap | 0.00 | 0.00 | 0.00 | 0.00 | 0.00 | 0.00 | 0.00 |  | 0.00 | 0.00 | 0.00 | 0.00 | 0.00 | 0.00 | 0.00 |
| APulREScap | **-0.44** | **0.14** | **-0.58** | **-0.45** | **-0.45** | **-0.45** | **-0.44** |  | **-0.15** | 0.08 | 0.00 | **-0.17** | **-0.16** | **-0.16** | **-0.17** |
| AKREScap | 0.00 | 0.00 | 0.00 | 0.00 | 0.00 | 0.00 | 0.00 |  | 0.00 | 0.00 | 0.00 | 0.00 | 0.00 | 0.00 | 0.00 |
| ALREScap | **-0.46** | **0.18** | **-0.74** | **-0.48** | **-0.48** | **-0.48** | **-0.47** |  | -0.01 | 0.10 | -0.01 | -0.02 | -0.01 | -0.01 | -0.02 |
| ASREScap | 0.00 | 0.00 | 0.00 | 0.00 | 0.00 | 0.00 | 0.00 |  | 0.00 | 0.00 | 0.00 | 0.00 | 0.00 | 0.00 | 0.00 |
| ARREScap | **-0.46** | **0.18** | **-0.74** | **-0.48** | **-0.48** | **-0.48** | **-0.47** |  | -0.01 | 0.10 | -0.01 | -0.02 | 0.00 | -0.02 | -0.02 |
| **Release rate constant of phagocytic cells** | | | | | | | |  |  |  |  |  |  |  |  |
| KGIRESrelease | **-0.49** | **0.19** | **-0.79** | **-0.51** | **-0.52** | **-0.51** | **-0.50** |  | -0.01 | **0.11** | -0.01 | -0.02 | -0.01 | -0.01 | -0.02 |
| KPulRESrelease | **-0.20** | **0.17** | **-0.71** | **-0.25** | **-0.26** | **-0.24** | **-0.22** |  | **0.76** | **0.09** | -0.01 | **0.72** | **0.74** | **0.74** | **0.72** |
| KKRESrelease | 0.00 | 0.00 | 0.00 | 0.00 | 0.00 | 0.00 | 0.00 |  | 0.00 | 0.00 | 0.00 | 0.00 | 0.00 | 0.00 | -0.02 |
| KLRESrelease | 0.00 | 0.00 | 0.00 | 0.00 | 0.00 | -0.01 | 0.00 |  | 0.05 | 0.00 | 0.00 | 0.04 | 0.05 | **-0.43** | 0.04 |
| KSRESrelease | **-0.46** | **0.18** | **-0.74** | **-0.48** | **-0.48** | **-0.48** | **-0.47** |  | -0.01 | 0.10 | -0.01 | -0.08 | -0.01 | -0.01 | -0.02 |
| KRRESrelease | 0.02 | 0.00 | 0.00 | 0.01 | -0.03 | 0.01 | 0.02 |  | **0.13** | 0.00 | 0.00 | **0.13** | **-0.58** | **0.14** | **0.13** |
| **Urinary, fecal and biliary excretion constant rate** | | | | | | | |  |  |  |  |  |  |  |  |
| KurineC | **-0.14** | 0.00 | 0.00 | **-0.12** | **-0.12** | **-0.13** | **-0.13** |  | **-0.42** | 0.00 | 0.00 | **-0.35** | **-0.39** | **-0.37** | **-0.35** |
| KfecesC | **-0.20** | -0.02 | **-0.38** | **-0.15** | **-0.14** | **-0.17** | **-0.18** |  | **-0.19** | -0.01 | **-0.98** | **-0.20** | **-0.19** | **-0.19** | **-0.20** |
| KbileC | 0.00 | 0.00 | 0.00 | 0.00 | 0.00 | -0.01 | 0.00 |  | -0.01 | 0.00 | 0.00 | -0.01 | -0.01 | -0.01 | -0.01 |

AUCB, AUCLu, AUCGI, AUCS, AUCRt, AUCL, and AUCK represent area-under-the-concentration curves of gold nanoparticles (AuNPs) in artery blood, lungs, gastrointestinal (GI) tract, spleen, rest of body, liver, and kidneys, respectively. K_max_ is maximum uptake rate constant for endocytosis/phagocytosis; K_release_ is the release rate constant of phagocytic cells; A_cap_ is endocytosis/phagocytosis uptake capacity. GI, Pul, K, L, R, and S stand for the GI tract, Pulmonary, Kidneys, Livers, Rest of body and Spleen, respectively. The values of |NSC| > 0.1 were highlighted in bold. Parameter value of around or greater than 0.5 were considered highly sensitive.

| **Table S12.** | Final multivariate linear regression models describing relationships between the physicochemical properties of AuNPs and biodistribution parameters following oral gavage |
| --- | --- |

| Tissue/Organ | *β*_0_ | *β*_1_ | *β*_2_ | *β*_3_ | *β*_4_ | *β*_5_ | *β*_6_ | *β*_7_ | *BIC* | Adj-*R*^2^ | *p*-value |
| --- | --- | --- | --- | --- | --- | --- | --- | --- | --- | --- | --- |
| ***Intravenous injection (IV)*** | | | | | | | | | | | |
| **Maximum endocytosis/phagocytosis uptake rate** | | | | | | | | |  | | |
| Lung | -468 |  |  |  | 31.6 |  |  | 15.2 | 16.2 | 0.99 | 0.028 |
| GI tract |  |  |  |  |  |  |  |  |  |  |  |
| Liver | -41.8 |  | 1.04 |  |  |  | 13.2 |  | -8.25 | 0.99 | 0.006 |
| Spleen | -52.7 |  |  |  |  | -4.91 |  | 2.32 | 11.1 | 0.93 | 0.152 |
| Kidney | 19.5 |  | 1.03 |  |  |  |  | -0.78 | -27.2 | 0.99 | 0.001 |
| Rest of body | 0.33 | 0.008 | -0.02 |  |  |  |  |  | -25.7 | 0.99 | 0.007 |
| **Endocytosis/phagocytosis uptake capacity** | | | | | | | | |  |  |  |
| Lung |  |  |  |  |  |  |  |  |  |  |  |
| GI tract |  |  |  |  |  |  |  |  |  |  |  |
| Liver |  |  |  |  |  |  |  |  |  |  |  |
| Spleen |  |  |  |  |  |  |  |  |  |  |  |
| Kidney |  |  |  |  |  |  |  |  |  |  |  |
| Rest of body |  |  |  |  |  |  |  |  |  |  |  |
| **Release rate constant of phagocytic cells** | | | | | | | | |  | | |
| Lung | 18.5 | 0.07 |  |  | -5.17 |  |  |  | 4.54 | 0.96 | 0.113 |
| GI tract |  |  |  |  |  |  |  |  |  |  |  |
| Liver | -0.237 |  | 0.872 |  |  | -1.84 |  |  | -11.85 | 0.99 | 0.008 |
| Spleen | 11.8 |  |  |  |  | 0.92 |  | -0.44 | -4.55 | 0.96 | 0.114 |
| Kidney | -0.007 |  | -0.02 | -0.02 |  |  |  |  | -27.15 | 0.99 | 0.015 |
| Rest of body | 3.43 |  |  |  | -0.77 | -0.43 |  |  | -1.25 | 0.896 | 0.186 |
| **Urinary and fecal excretion constant rate** | | | | | | | | |  |  |  |
| Urine | -7.76E-5 |  | 3.8E-5 |  | 1.7E-4 |  |  |  | -88.3 | 0.99 | 0.009 |
| Feces | 0.266 | -0.009 |  | -0.06 |  |  |  |  | -31.9 | 0.99 | 0.006 |
| Biliary | 0.325 | 0.002 |  |  | 0.014 |  |  |  | -35.3 | 0.99 | 0.02 |

*β*_0_ and *β_i_* represent intercept and slope of variables included in the multivariate linear regression model, respectively [1: hydrodynamic diameter (HD); 2: surface area (SA); 3: Zeta potential (Z); 4: log(HD); 5: log(SA); 6: log(Z); 7: log-transformed number of NPs, log(NPs)]. The values in bold represent statistical significance of *p*<0.05.

**Abbreviations:** BIC, Bayesian information criterion; Adj-*R*^2^, adjusted *R*^2^.

| **Table S13.** | Final multivariate linear regression models describing relationships between the physicochemical properties of AuNPs and biodistribution parameters following intratracheal instillation. |
| --- | --- |

| Tissue/Organ | *β*_0_ | *β*_1_ | *β*_2_ | *β*_3_ | *β*_4_ | *β*_5_ | *β*_6_ | *β*_7_ | *BIC* | Adj-*R*^2^ | *p*-value |
| --- | --- | --- | --- | --- | --- | --- | --- | --- | --- | --- | --- |
| ***Intravenous injection (IV)*** | | | | | | | | | | | |
| **Maximum endocytosis/phagocytosis uptake rate** | | | | | | | | |  | | |
| Lung | -2.16E3 |  |  |  | 229 |  |  | 60.6 | 47.5 | 0.96 | 0.019 |
| GI tract |  |  |  |  |  |  |  |  |  |  |  |
| Liver |  |  |  |  |  |  |  |  |  |  |  |
| Spleen | 0.013 |  |  |  | -3.5E-4 |  |  | 1.13E-4 | -94.4 | 0.99 | 0.003 |
| Kidney | 20.5 |  | -5.48 |  |  | 32.4 |  |  | 32.16 | 0.94 | 0.028 |
| Rest of body |  |  |  |  |  |  |  |  |  |  |  |
| **Endocytosis/phagocytosis uptake capacity** | | | | | | | | |  |  |  |
| Lung |  |  |  |  |  |  |  |  |  |  |  |
| GI tract |  |  |  |  |  |  |  |  |  |  |  |
| Liver |  |  |  |  |  |  |  |  |  |  |  |
| Spleen |  |  |  |  |  |  |  |  |  |  |  |
| Kidney |  |  |  |  |  |  |  |  |  |  |  |
| Rest of body |  |  |  |  |  |  |  |  |  |  |  |
| **Release rate constant of phagocytic cells** | | | | | | | | |  | | |
| Lung | 0.009 |  | -5.3E-4 |  |  | 0.001 |  |  | -63.3 | 0.98 | 0.006 |
| GI tract |  |  |  |  |  |  |  |  |  |  |  |
| Liver |  |  |  |  |  |  |  |  |  |  |  |
| Spleen | 1.82 | -0.001 |  |  |  |  |  | -0.06 | -17.12 | 0.97 | 0.014 |
| Kidney | 0.39 |  | -0.02 |  | 0.017 |  |  |  | -29.7 | 0.99 | 0.002 |
| Rest of body |  |  |  |  |  |  |  |  |  |  |  |
| **Urinary and fecal excretion constant rate** | | | | | | | | |  |  |  |
| Urine | 3.9E-4 |  | -1.03E-4 |  |  |  | 6.21E-4 |  | -76.4 | 0.94 | 0.03 |
| Feces | 2.6E-4 | -1.69E-8 |  |  |  |  | -9.55E-7 |  | -117 | 0.274 | 0.362 |
| Biliary | 0.09 | 7.25E-4 |  | 4.5E-3 |  |  |  |  | -46.8 | 0.99 | 0.002 |

*β*_0_ and *β_i_* represent intercept and slope of variables included in the multivariate linear regression model, respectively [1: hydrodynamic diameter (HD); 2: surface area (SA); 3: Zeta potential (Z); 4: log(HD); 5: log(SA); 6: log(Z); 7: log-transformed number of NPs, log(NPs)]. The values in bold represent statistical significance of *p*<0.05.

**Abbreviations:** BIC, Bayesian information criterion; Adj-*R*^2^, adjusted *R*^2^.

| **Table S14.** | Main physicochemical characteristics of the AuNPs in the selected pharmacokinetic studies used for model evaluation in this study | | | | | | |
| --- | --- | --- | --- | --- | --- | --- | --- |
|  | Morais et al. (2012) | | | | |  | Fraga et al. (2012) |
| Surface coating | Citrate | 11-MUA | CALNN | CALND | CALNS |  | Citrate |
| Size (nm) | 18 ± 5 | 18 ± 5 | 18 ± 5 | 18 ± 5 | 18 ± 5 |  | 16.1 ± 2.8 |
| HD (nm) | 22.4 | 33.2 | 33.7 | 35.1 | 35.1 |  | 23.31 |
| Zeta (mV) | −44.7 ± 7.5 | −37.3 ± 8.4 | −47.1 ± 6.0 | −43.6 ± 9.2 | −40.5 ± 7.0 |  | -49.4 ± 12.8 |
| Mass of AuNPs (mg/kg) | 0.6–1 | 0.6–1 | 0.6–1 | 0.6–1 | 0.6–1 |  | 0.7 |
| NPs (#/mL) | - | - | - | - | - |  | 2.7 × 10^12^ |
| SA (cm^2^) | NA | NA | NA | NA | NA |  | 839.0 ± 283.2 |

Abbreviations: Size, core diameter; HD, Hydrodynamic radius; Zeta, Zeta potential; Mass of AuNPs, the administered AuNPs dose; NPs, the number of AuNPs in the media; 11-MUA, 11-mercaptoundecanoic acid; CALND, Cys-Ala-Leu-Asn-Asp; CALNN, Cys-Ala-Leu-Asn-Asn; CALNS, Cys-Ala-Leu-Asn-Ser.

Note: all values were collected from Morais et al. (2012) [17] and Fraga et al. (2014) [18].

| **Table S15** | Model-predicted and measured amount of AuNPs in blood, GI, Kidneys, Liver, Lungs and spleen | | | | | | | | | | | |
| --- | --- | --- | --- | --- | --- | --- | --- | --- | --- | --- | --- | --- |
|  | **Blood** | | **GI** | | **Kidneys** | | **Liver** | | **Lungs** | | **Spleen** | |
| **Study 1^a^** | Mod | Obs | Mod | Obs | Mod | Obs | Mod | Obs | Mod | Obs | Mod | Obs |
| **Citrate** | 0.50±0.61^c^ | 1.27±0.43 | 0.37±0.31 | 0.02±0.01 | 0.03±0.02 | 0.08±0.02 | 95±3.16 | 58±2.57 | 0.04±0.02 | 0.72±0.02 | 1.48±0.59 | 1.34±0.23 |
| **11-MUA** | 0.43±0.50 | 1.13±0.7 | 0.32±0.24 | 0.01±0.03 | 0.03±0.02 | 0.03±0.08 | 95±2.67 | 59±4.08 | 0.04±0.00 | 0.44±0.00 | 1.43±0.56 | 2.26±0.52 |
| **CALNN** | 0.53±0.65 | 0.32±0.13 | 0.35±0.28 | 0.24±0.01 | 0.03±002 | 0.2±0.2 | 95±3.28 | 86±7.13 | 0.04±0.00 | 0.53±0.00 | 1.48±0.60 | 2.79±0.30 |
| **CALND** | 0.49±0.60 | 0.19±0.08 | 0.33±0.27 | 0.01±0.01 | 0.03±0.02 | 0.05±0.11 | 95±3.10 | 77±2.52 | 0.03±0.00 | 0.88±0.01 | 1.48±0.59 | 2.51±0.59 |
| **CALNS** | 0.47±0.56 | 0.38±0.07 | 0.32±0.25 | 0.52±0.00 | 0.03±0.02 | 0.01±0.04 | 96±2.66 | 74±4.48 | 0.03±0.00 | 0.52±0.00 | 0.39±0.17 | 1.88±0.13 |
| **Study 2^b^** |  |  |  |  |  |  |  |  |  |  |  |  |
| **Citrate** | 0.21±0.35 | NA | 0.17±0.16 | NA | 0.026±0.02 | 0.003±0.03 | 98±1.01 | 28.31±3.4 | 0.03±0.00 | 0.03±0.01 | 0.71±0.76 | 0.89±0.31 |

Note: NA, value not available in the experimental study; Mod, model predicted AuNPs amount in specific tissues (expressed as % of the injected dose).; Obs, observed AuNPs amount in specific tissues (expressed as % of the injected dose); MUA, 11-mercaptoundecanoic acid; CALND, Cys-Ala-Leu-Asn-Asp; CALNN, Cys-Ala-Leu-Asn-Asn; CALNS, Cys-Ala-Leu-Asn-Ser.

^a^ Male Wistar rats with the body weight ranging from 200 to 300 g were intravenously administrated with AuNPs of 1 mg/kg with the coating of 11-MUA, CALNN, CALND and CALNS. The data were collected at 24 hours after injection. Data are from Morais et al. (2012) [17].

^b^ Wistar rats were exposed to AuNPs of 0.7 mg/kg with the single injection in the rat tail vein. The AuNPs concentration was then determined at 28 days post-injection. Data are from Fraga et al. (2014) [18].

^c^ Measured and simulated values are expressed as means ± SD (expressed as % of the injected dose).

1. **Supplementary Figures**

**
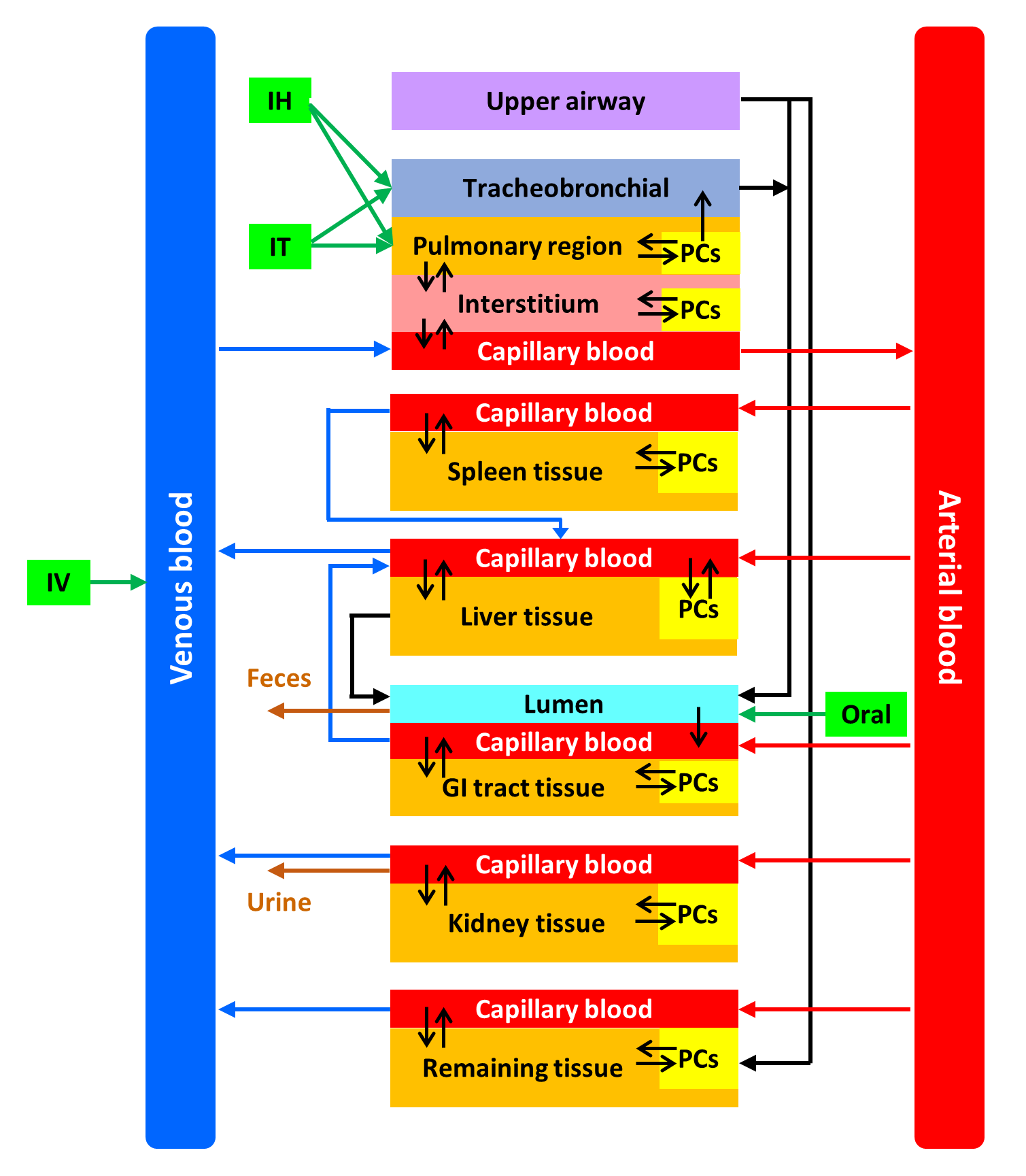
**

| **Fig. S1** | Schematic diagram of the multi-route physiologically based pharmacokinetic (PBPK) model for 1.4, 5, 18, 23, 80, and 200 nm gold nanoparticles (AuNPs) in adult rats. Four administration routes are presented in this model: intravenous (IV), oral gavage (Oral), intratracheal instillation (IT), and endotracheal inhalation (IH). Except blood, each compartment is divided into three major parts: capillary blood, tissue interstitium, and endocytic/phagocytic cells (PCs). The dashed arrow from “IH” to “Upper airway” was added to the model structure so that the model is generic and can be used to simulate both whole-body inhalational exposure (i.e., placing the rat in an inhalational chamber) and endotracheal inhalational exposure (i.e., the rat is intubated with an endotracheal tube). Please note that in our study, we used endotracheal inhalational exposure, so extrathoracic airways and the pharynx, larynx, and the first third of the trachea were bypassed and no AuNPs were deposited in the upper airway. Therefore, we set this fraction to be zero. |
| --- | --- |

**

**

| **Fig. S2** | PBPK model simulation results compared with 24-h IV-based pharmacokinetic data of 1.4, 5, 18, 80, and 200 nm AuNPs in healthy rats from Hirn et al. (2011) [10]. Symbols represent measured data (mean ± SD) (percentage of initial dose, %ID) in blood (red), liver (purple), and spleen (green) with dashed lines displaying corresponded simulation results. |
| --- | --- |

**

**

| **Fig. S3** | PBPK model simulation results compared with 24-h pharmacokinetic data of 5, 18, 80, and 200 nm AuNPs in rats following oral gavage [11]. Symbols demonstrate measured data (mean ± SD) (%ID) in blood (red) and gastrointestinal (GI) tract (orange) with dashed lines displaying simulation results. |
| --- | --- |

**

**

| **Fig. S4** | PBPK model calibration with 24-h pharmacokinetic data for 1.4, 5, 18, 80, and 200 nm AuNPs in rats after intratracheal instillation [13]. Symbols are measured data (mean ± SD) (percentage of received initial peripheral lung dose, %IPLD) in lungs (pink) and GI tract (orange) and dashed lines represent simulation results. |
| --- | --- |





| **Fig. S5** | PBPK model calibration with 28-d pharmacokinetic data [12] in rats after inhalation of 23 nm aerosolized AuNPs via endotracheal tube for 2 h. Symbols represent measured data (mean ± SD) (%IPLD) in lungs (pink), GI tract (orange), liver (purple), spleen (green), and remaining (gray) with dashed lines demonstrating simulation results. |
| --- | --- |

**
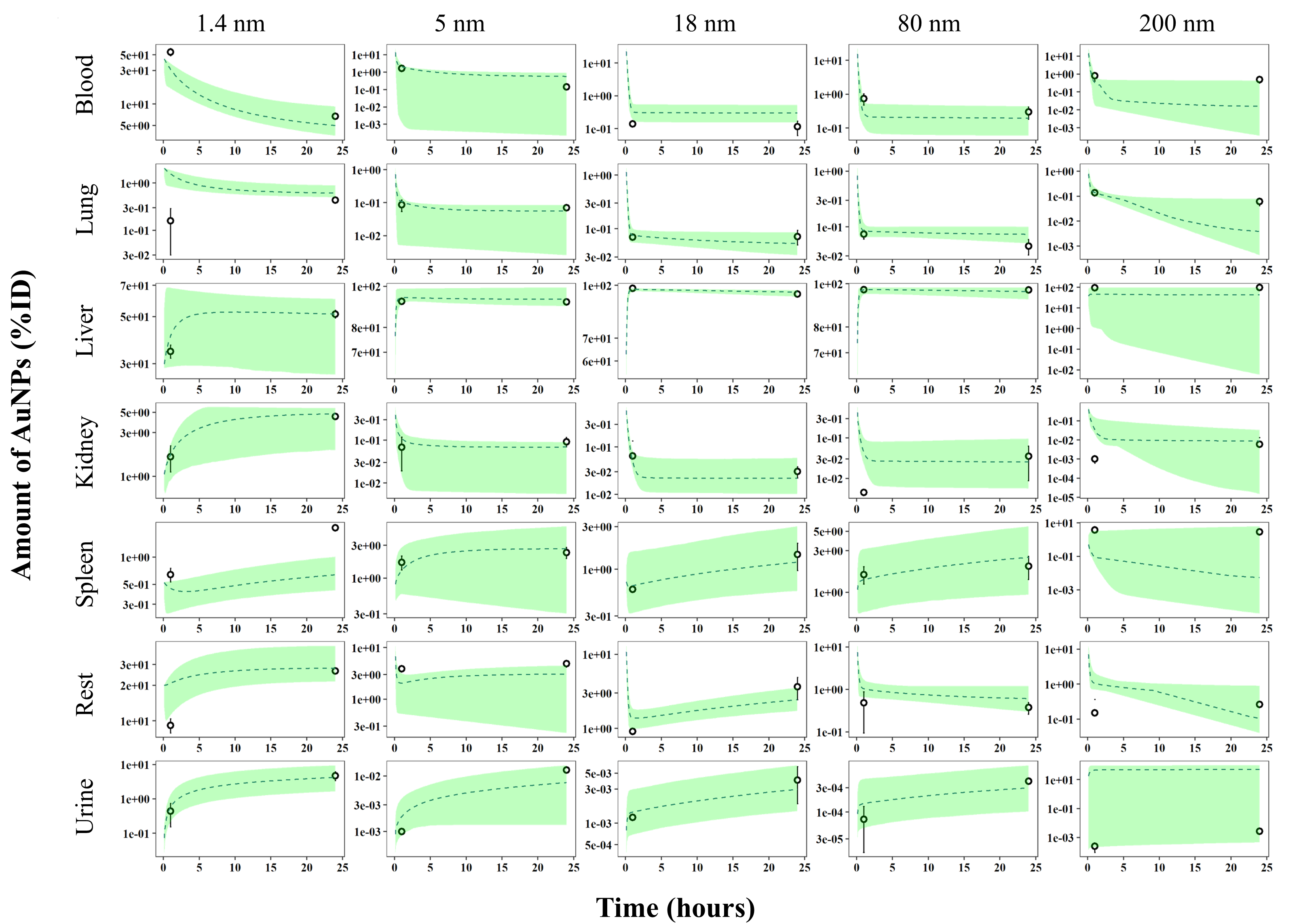
**

| **Fig. S6** | PBPK model calibration results with pharmacokinetic data of 1.4, 5, 18, 80 and 200 nm AuNPs in rats after intravenous injection. Symbols and error bars (mean ± SD) represent measured amounts and dashed lines represent simulated amounts of AuNPs (percent of the initial dose [%ID]) in blood, lungs, liver, kidney, spleen, remaining tissues (rest) and urine. |
| --- | --- |

**
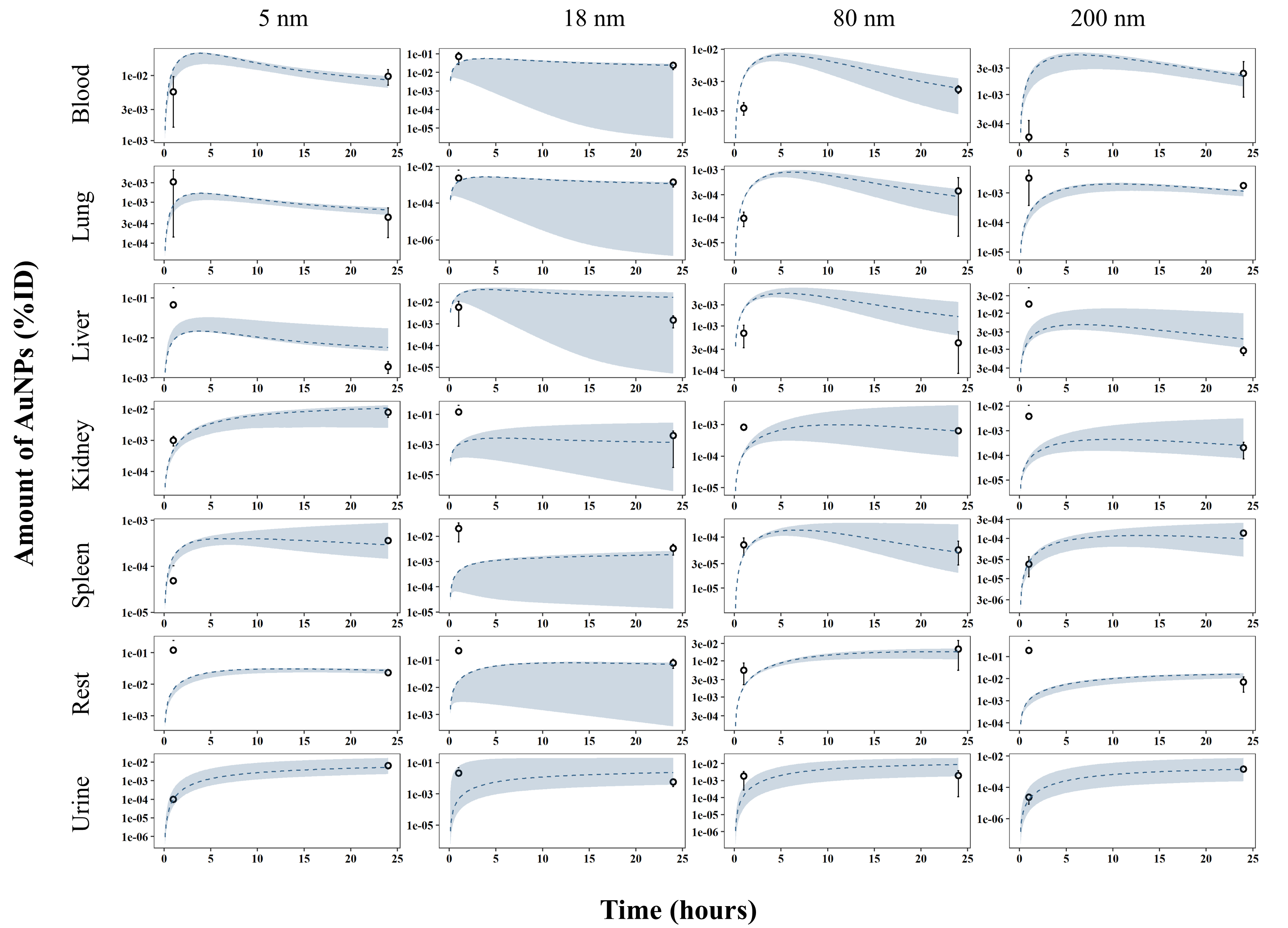
**

| **Fig. S7** | PBPK model calibration results with pharmacokinetic data of 5, 18, 80 and 200 nm AuNPs in rats after oral administration. Symbols and error bars (mean ± SD) represent measured amounts and dashed lines represent simulated amounts of AuNPs (percent of the initial dose [%ID]) in blood, lungs, liver, kidney, spleen, remaining tissues (rest) and urine. |
| --- | --- |

**
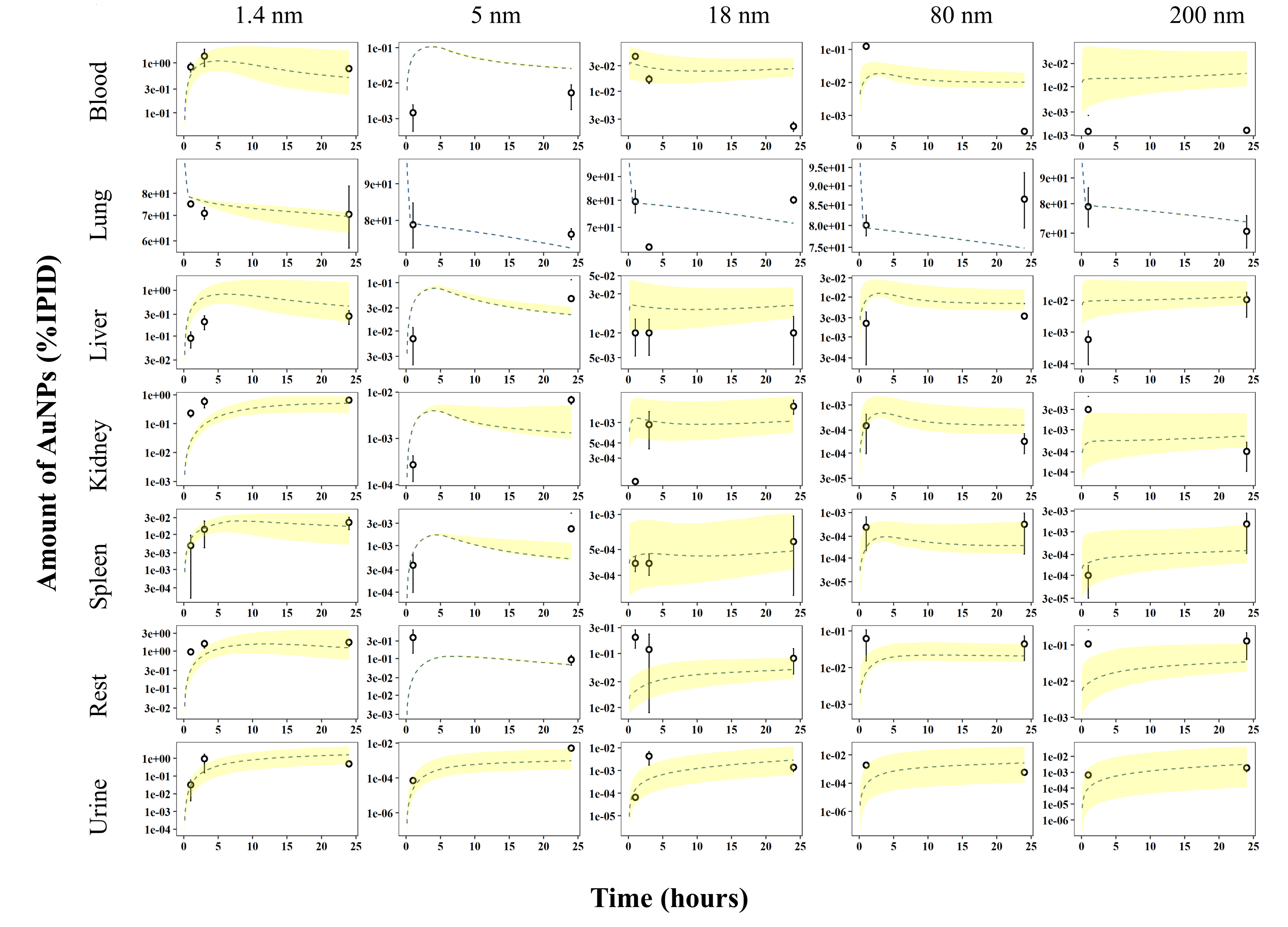
**

| **Fig. S8** | PBPK model calibration results with pharmacokinetic data of 1.4, 5, 18, 80 and 200 nm AuNPs in rats after intratracheal instillation. Symbols and error bars (mean ± SD) represent measured amounts and dashed lines represent simulated amounts of AuNPs (percent of the initial peripheral lung dose [%IPLD]) in blood, lungs, liver, kidney, spleen, remaining tissues (rest) and urine. |
| --- | --- |


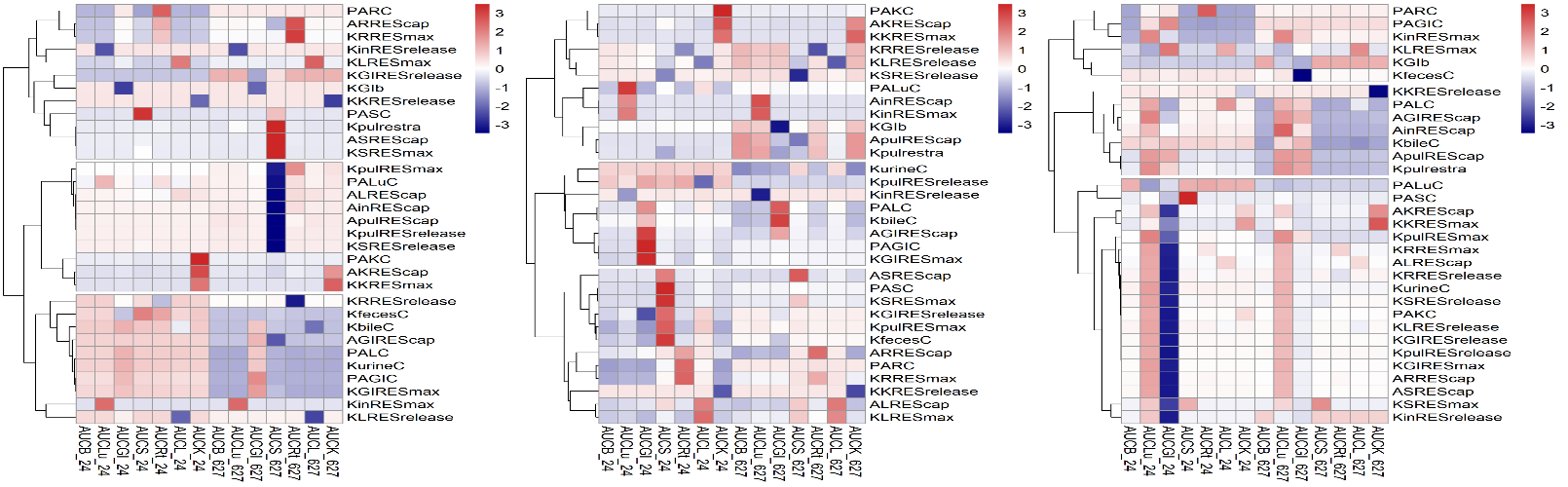

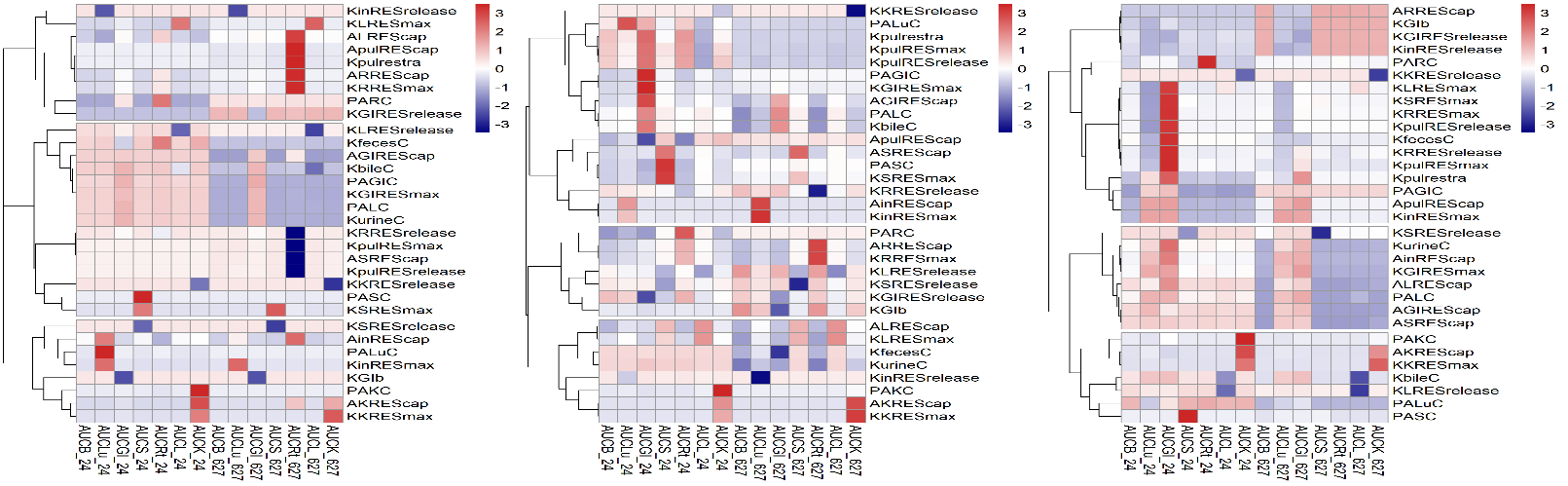

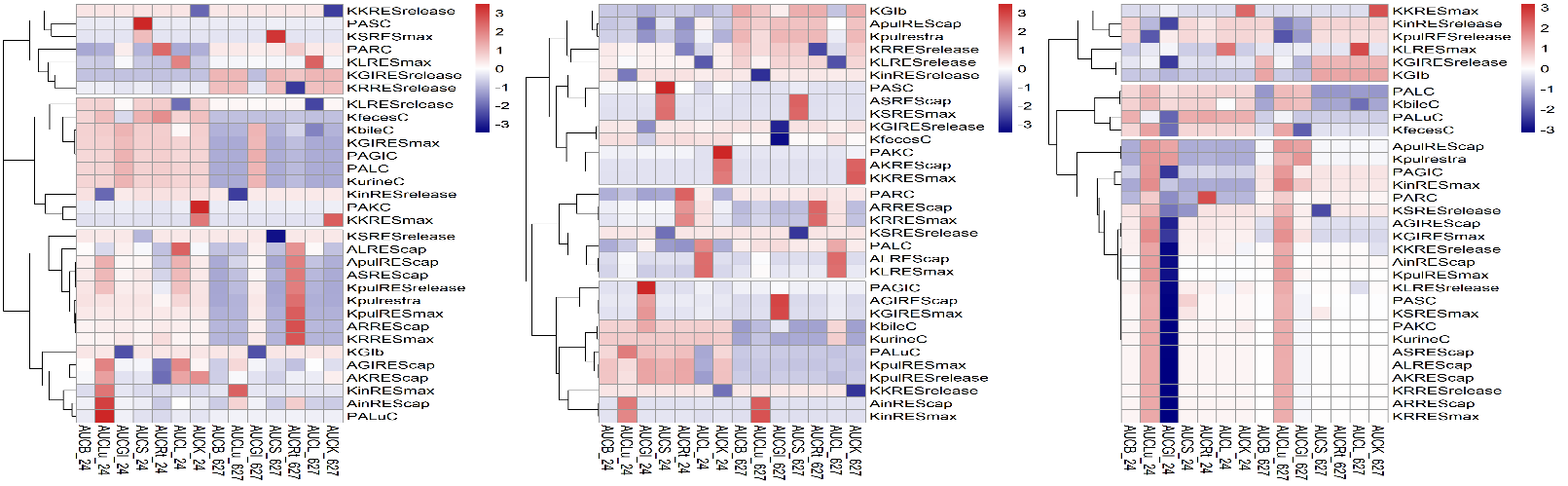


**(A)**

**(B)**

**(C)**

**(A)**

**Oral-18 nm**

**IV-18 nm**

**IT-18 nm**

**(E)**

**(F)**

**(D)**

**(G)**

**(H)**

**(I)**

**Oral-80 nm**

**Oral-200 nm**

**IV-80 nm**

**IV-200 nm**

**IT-80 nm**

**IT-200 nm**

| **Fig. S9** | Heat map plot of the normalized sensitivity coefficient (NSC) for comparative sensitivity analyses for (A, B, C) 18, (D, E, F) 80 and (G, H, I) 200 nm AuNPs at 24 h and 672 h following (A, D, G) oral administration (Oral), (B, E, H) intravenous injection (IV) and (C, F, I) intratracheal instillation (IT). The plot identifies highly influential parameters with darker colors (red or blue) to the 24-h and 672-h dose metrics. AUCB, AUCLu, AUCGI, AUCS, AUCL, AUCK and AUCRt represent area-under-the-concentration curve of gold nanoparticles (AuNPs) in blood, lungs, gastrointestinal (GI) tract, spleen, liver, and kidneys and remaining tissues, respectively. |
| --- | --- |

**a**

**Observed values (%ID)**

**b**

**O/P ratios**

**Predicted values (%ID or %IPLD)**

| **Fig. S10** | Comparisons of model prediction (x-axis) with observed data (y-axis) for the validation datasets. a. Global evaluation of goodness of model fit from the PBPK model validation results for pharmacokinetic data from rats after IV administration of AuNPs with different surface coatings, including: citrate (the same coating with our calibration data), 11-mercaptoundecanoic acid (11-MUA), Cys-Ala-Leu-Asn-Asn (CALNN), Cys-Ala-Leu-Asn-Asp (CALND) and Cys-Ala-Leu-Asn-Ser (CALNS). b. Observed-to-predicted (O/P) ratio versus model prediction plot. In both panels, the different symbol shapes and colors are used for different tissues (blood, GI, kidney, liver, lung and spleen) and surfactants (citrate, 11-MUA, CALNN, CALND and CALNS), respectively. In panel b, the dashed line represents over a O/P ratio of 2 or lower 0.5. |
| --- | --- |

1. **Tutorial for Nano-iPBPK**
   1. **Introduction**

The final PBPK model was converted to a web-based interface using the R Shiny. The objective of this web interface, i.e., Nano-iPBPK is to provide a tool to predict biodistribution of different types of AuNPs following different routes of exposure in rats based on the physicochemical properties of the studied AuNPs. Below is detailed instruction on how to use this web interface.

- 1. **Instruction**

1. Open the App

Currently, the App has been published and available online on the ShinyApps web server (<http://www.shinyapps.io/>). The user can access through https://pbpk.shinyapps.io/NanoiPBPK/.

1. Interface Structure


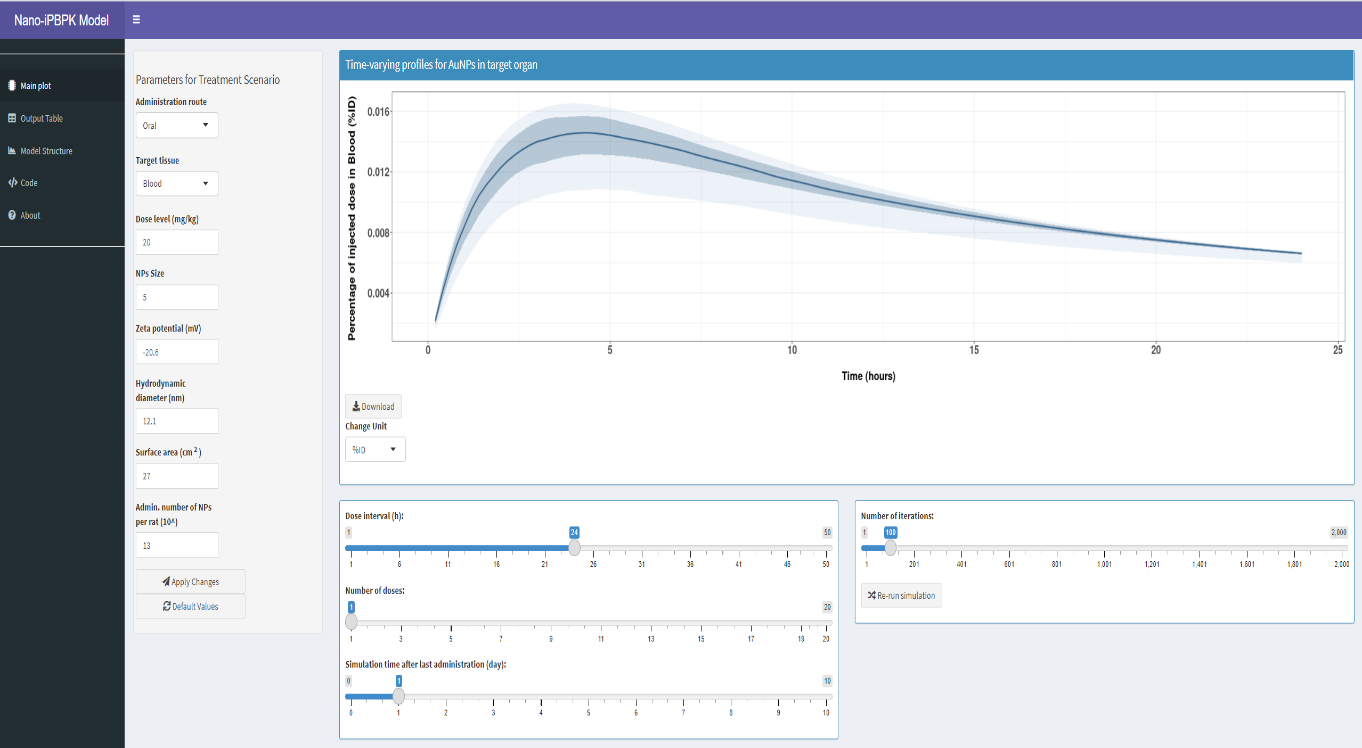
 The Nano-iPBPK interface contains four major parts including ‘Side Panel’, ‘Parameter Input Panel’, ‘Slider Controls Panel’ and ‘Output Panel’.

**Output Panel**

**Parameter input Panel**

**Side Panel**

**Slider Controls Panel**

The Side Panel contains ‘Model Parameters’, ‘Output Table’, ‘Model Structure’, ‘Code’, and ‘About’ tabs. By clicking the tab name, you can switch among these tabs.

The ‘Model Parameter’ tabs have parameter input panel. Four different types of controllers in this part, such as dropdown lists, switching buttons, input boxes and slider controllers. By scrolling down the dropdown button, the user can choose different choices to simulate the result. For switching buttons, the user can choose in-between different conditions. For input boxes, the user can directly input the value or use the arrow to increase or decrease the default value provided. For the slider controllers, the user can change the simulation by adjusting the range of values.


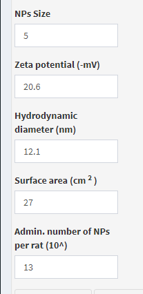

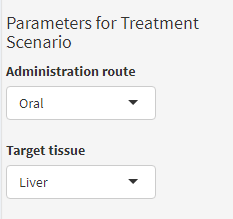


**Dropdown List**

**Switching Button**

**Input box**

**Slider Controllers**


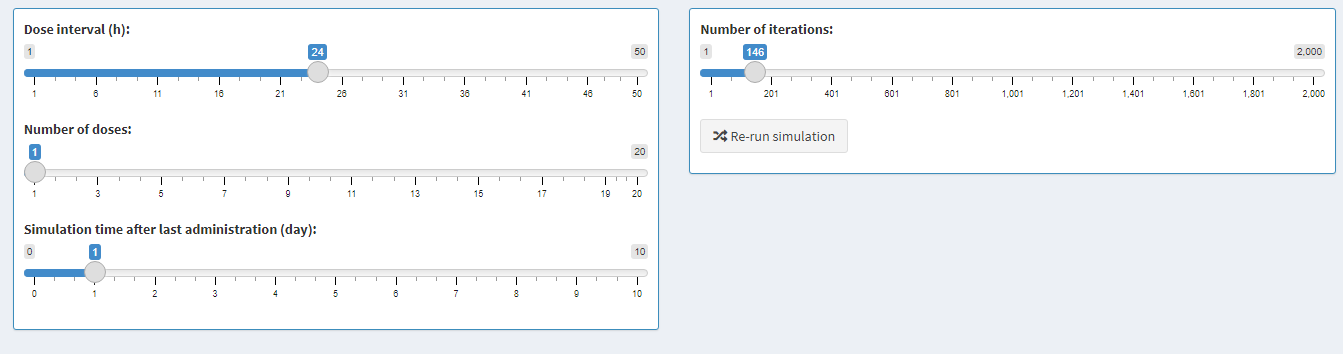


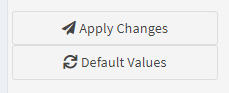


For ‘Main Plot’, and ‘Output Table’, the simulation results can be downloaded to your local computer by clicking the output buttons ‘Download’. This will show the simulation results using the parameter values you choose. The output panels in ‘Model Structure’, ‘Code’, and ‘About’ tabs provide additional information about the model. You can download the table by using the ‘Download’ buttons.


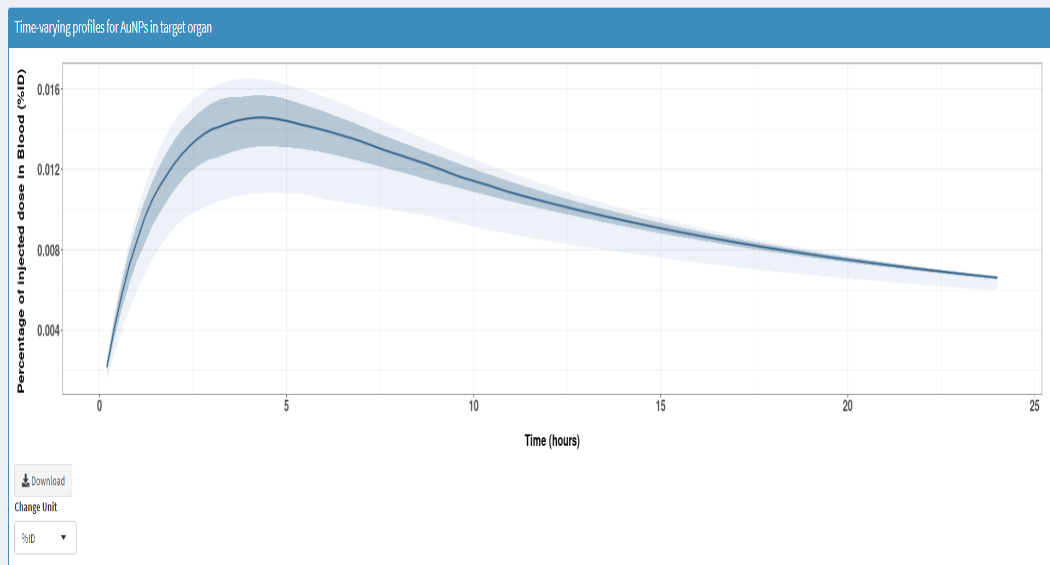


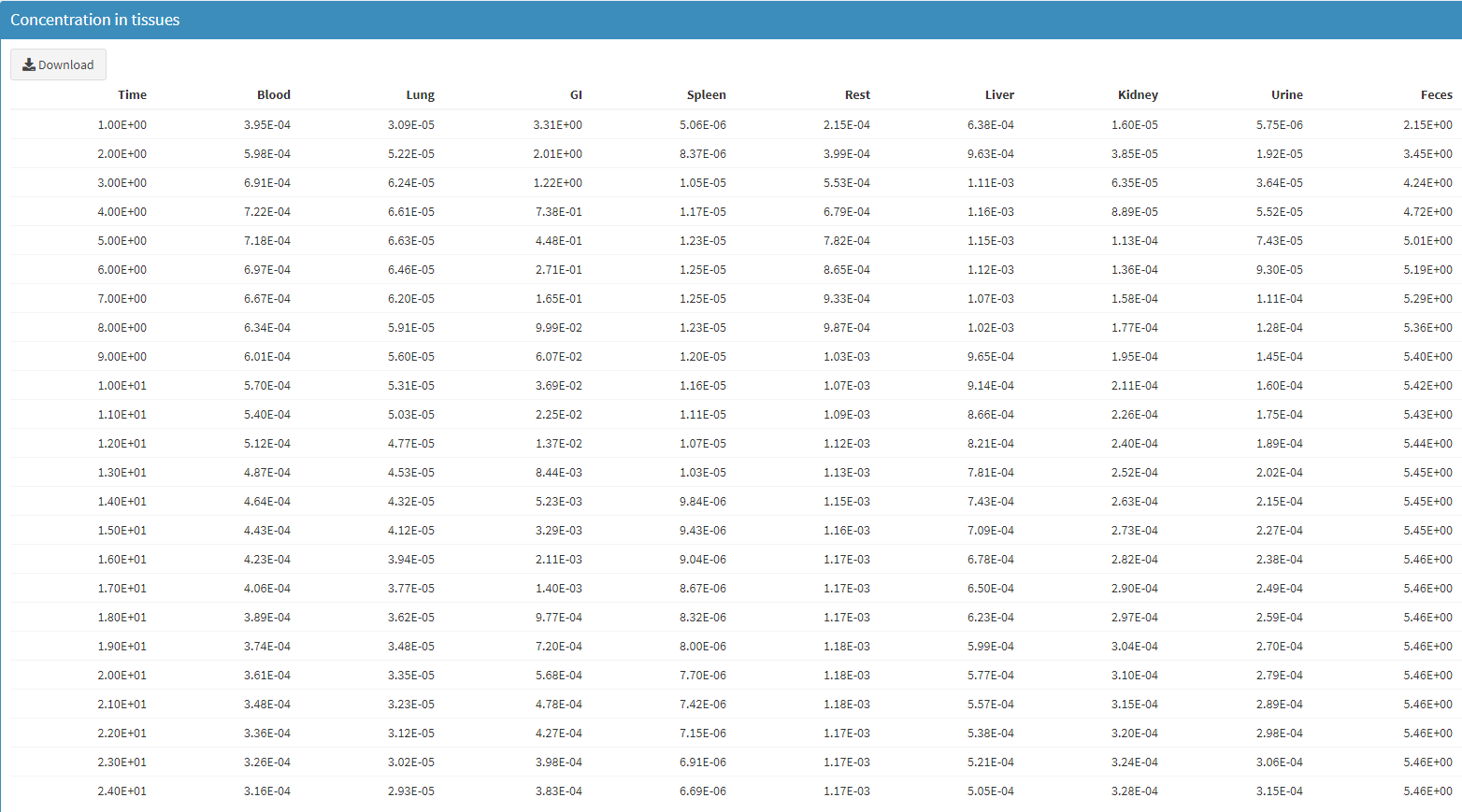


**Download button**

- 1. **Example**

The user can use the main tab (‘Main plot’) to predict the biodistribution in multiple organs following IV, oral, IT and IH administration to AuNPs. By changing values of related key parameters, the model will provide the time-varying profiles with 95% CI in selected organs as output.

**Step 1:** Choose “Main Plot” tab (this is the default page, when you open the simulator)


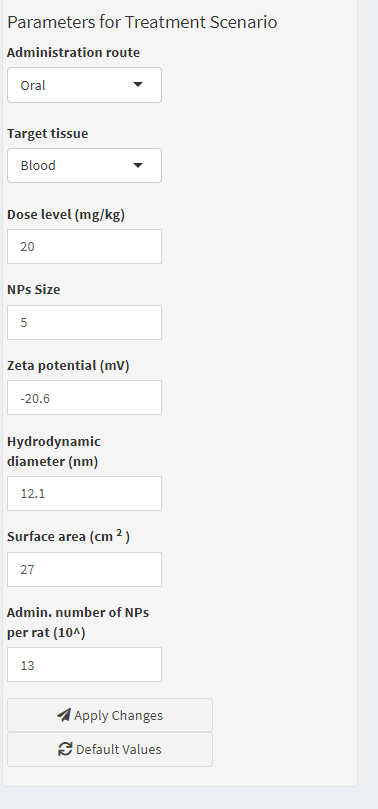

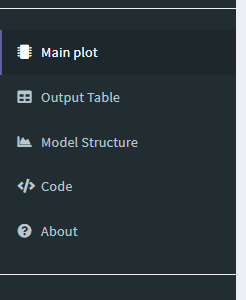


**Step Two**: The user can run the model by using default values or changing parameter values and then click the ‘Apply Changes’ button at the bottom of the Parameter Input Panel. When the model starts running, you will see a progress bar “Creating plot Please Wait” at the right bottom corner. The default setting includes 100 iterations in a Monte Carlo simulation, so the simulation will be completed within a few seconds depending on individual computer configurations.


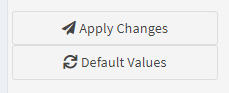


**Step Three**: The user can change the parameter values by using dropdown lists or input boxes. For example, if the user wants to know how much AuNPs accumulate in the liver following repeated oral administration with 20 mg/kg/day AuNPs for 3 days with 24-hour intervals, the user can choose “Oral” in Administration route, “Liver” in Target tissue, “20 mg/kg” in Dose level. Other parameters can be default values. In the slider controller, the user can pull the slider to “3” in the “Number of doses”. All other values are set up as default. After clicking the ‘Apply Changes’ button, the model starts to run simulation with the parameter values you choose. When the simulation finishes, the model will output a time-varying profile in liver with 100 iterations in Monte Carlo simulation.


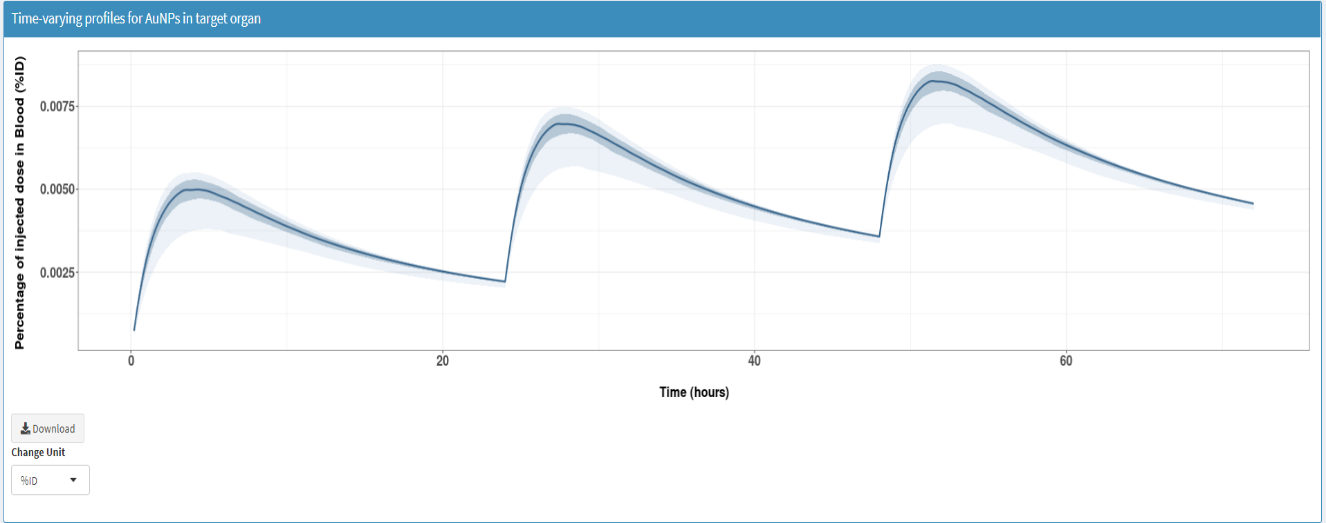


Here, the y-axis and x-axis were presented as percentage of total injected dose (%ID) and time (hours). We also can change the unit from “%ID” to “mg/L” as the figure listed below:


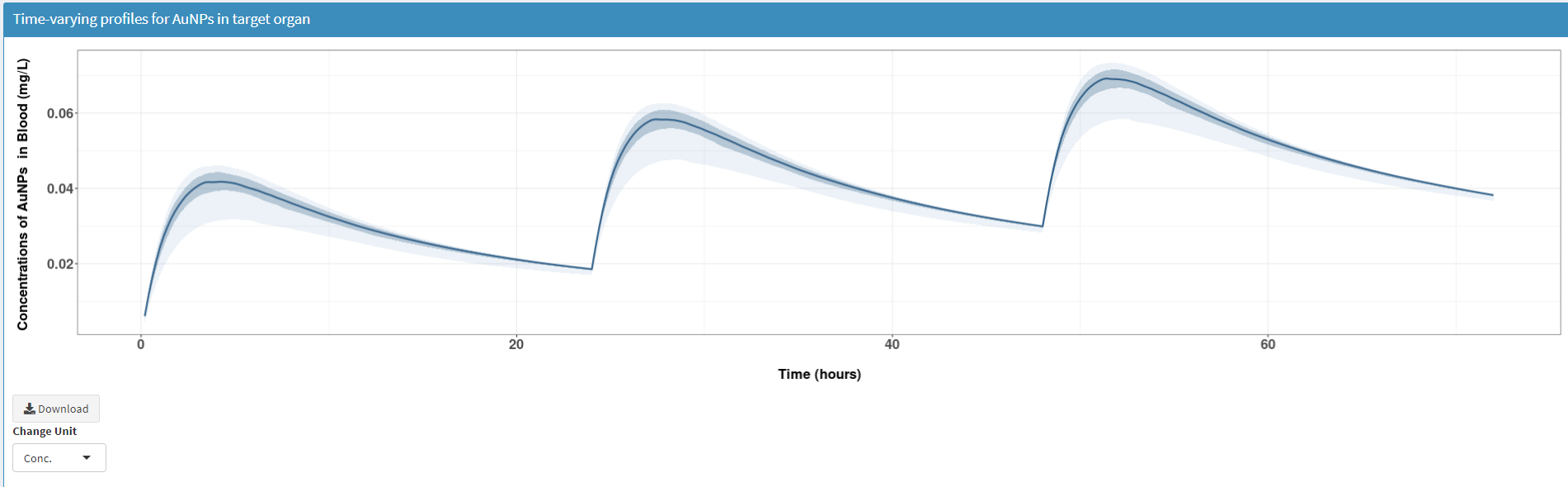


**Step Four:** The user can predict the biodistribution of AuNPs with different physicochemical properties of nanoparticles despite their biodistribution parameters are unknown. For example, the user may want to know the biodistribution profiles of AuNPs with the hydrodynamic diameter of 20 nm and the Zeta potential of -40 mV. You can input parameters values in the ‘Hydrodynamic Diameter’ and ‘Zeta Potential (mV)’ in the input boxes and then click the ‘Apply Changes’ button. All other parameters were the same as “Step Three” and run the model. The output results as below:


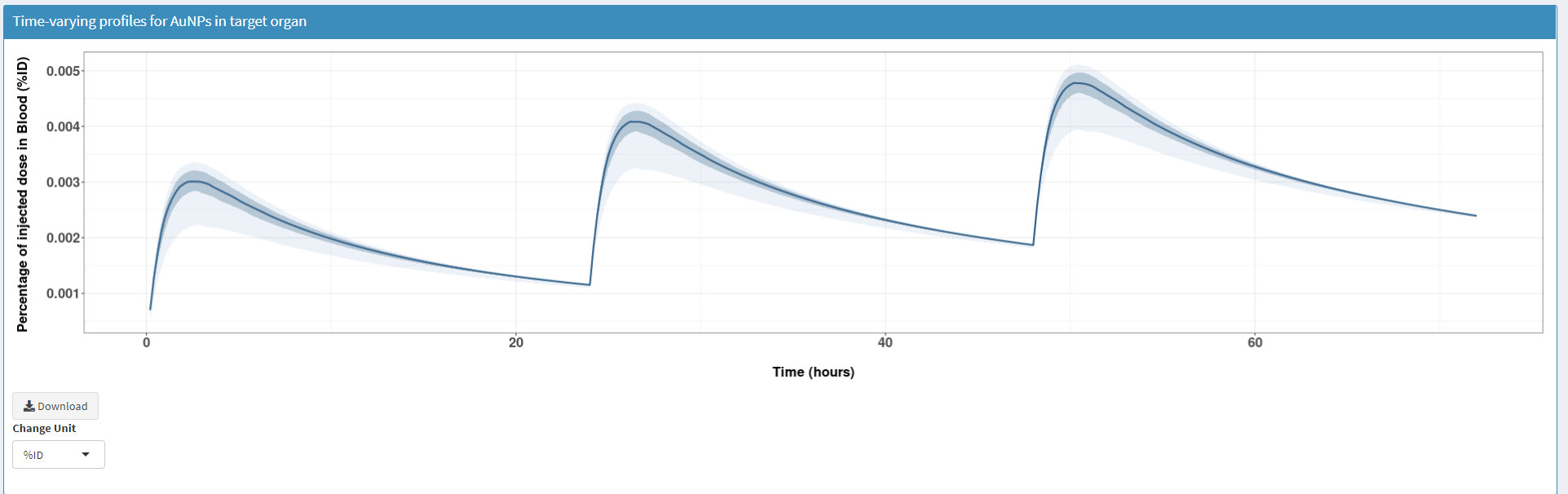


1. **References**

1. Lin Z, Monteiro-Riviere NA, Riviere JE: **A physiologically based pharmacokinetic model for polyethylene glycol-coated gold nanoparticles of different sizes in adult mice**. *Nanotoxicology* 2016, **10**(2):162-172.

2. Lin Z, Monteiro-Riviere NA, Kannan R, Riviere JE: **A computational framework for interspecies pharmacokinetics, exposure and toxicity assessment of gold nanoparticles**. *Nanomedicine (Lond)* 2016, **11**(2):107-119.

3. Owens DE, Peppas NA: **Opsonization, biodistribution, and pharmacokinetics of polymeric nanoparticles**. *Int J Pharmaceut* 2006, **307**(1):93-102.

4. Liang X, Wang H, Grice JE, Li L, Liu X, Xu ZP, Roberts MS: **Physiologically Based Pharmacokinetic Model for Long-Circulating Inorganic Nanoparticles**. *Nano Lett* 2016, **16**(2):939-945.

5. Sweeney LM, MacCalman L, Haber LT, Kuempel ED, Tran CL: **Bayesian evaluation of a physiologically-based pharmacokinetic (PBPK) model of long-term kinetics of metal nanoparticles in rats**. *Regul Toxicol Pharm* 2015, **73**(1):151-163.

6. Li M, Zou P, Tyner K, Lee S: **Physiologically Based Pharmacokinetic (PBPK) Modeling of Pharmaceutical Nanoparticles**. *Aaps J* 2017, **19**(1):26-42.

7. Kreyling WG, Semmler-Behnke M, Takenaka S, Moller W: **Differences in in the Biokinetics of Inhaled Nano- versus Micrometer-Sized Particles**. *Accounts Chem Res* 2013, **46**(3):714-722.

8. Davies B, Morris T: **Physiological-parameters in laboratory-animals and humans**. *Pharmaceut Res* 1993, **10**(7):1093-1095.

9. Brown RP, Delp MD, Lindstedt SL, Rhomberg LR, Beliles RP: **Physiological parameter values for physiologically based pharmacokinetic models**. *Toxicol Ind Health* 1997, **13**(4):407-484.

10. Hirn S, Semmler-Behnke M, Schleh C, Wenk A, Lipka J, Schaffler M, Takenaka S, Moller W, Schmid G, Simon U *et al*: **Particle size-dependent and surface charge-dependent biodistribution of gold nanoparticles after intravenous administration**. *Eur J Pharm Biopharm* 2011, **77**(3):407-416.

11. Schleh C, Semmler-Behnke M, Lipka J, Wenk A, Hirn S, Schaffler M, Schmid G, Simon U, Kreyling WG: **Size and surface charge of gold nanoparticles determine absorption across intestinal barriers and accumulation in secondary target organs after oral administration**. *Nanotoxicology* 2012, **6**(1):36-46.

12. Kreyling WG, Moller W, Holzwarth U, Hirn S, Wenk A, Schleh C, Schaffler M, Haberl N, Gibson N, Schittny JC: **Age-dependent rat lung deposition patterns of inhaled 20 nanometer gold nanoparticles and their quantitative biokinetics in adult rats**. *ACS Nano* 2018, **12**(8):7771-7790.

13. Kreyling WG, Hirn S, Moller W, Schleh C, Wenk A, Celik G, Lipka J, Schaffler M, Haberl N, Johnston BD *et al*: **Air-blood barrier translocation of tracheally instilled gold nanoparticles inversely depends on particle size**. *ACS Nano* 2014, **8**(1):222-233.

14. Carpenter B, Gelman A, Hoffman MD, Lee D, Goodrich B, Betancourt M, Riddell A, Guo JQ, Li P, Riddell A: **Stan: A Probabilistic Programming Language**. *J Stat Softw* 2017, **76**(1):1-29.

15. Brooks SP, Gelman A: **General methods for monitoring convergence of iterative simulations**. *J Comput Graph Stat* 1998, **7**(4):434-455.

16. Saiki C, Matsuoka T, Mortola JP: **Metabolic-Ventilatory Interaction in Conscious Rats - Effect of Hypoxia and Ambient-Temperature**. *J Appl Physiol* 1994, **76**(4):1594-1599.

17. Morais T, Soares ME, Duarte JA, Soares L, Maia S, Gomes P, Pereira E, Fraga S, Carmo H, Bastos MDL: **Effect of surface coating on the biodistribution profile of gold nanoparticles in the rat**. *Eur J Pharm Biopharm* 2012, **80**(1):185-193.

18. Fraga S, Brandao A, Soares ME, Morais T, Duarte JA, Pereira L, Soares L, Neves C, Pereira E, Bastos MD *et al*: **Short- and long-term distribution and toxicity of gold nanoparticles in the rat after a single-dose intravenous administration**. *Nanomed-Nanotechnol* 2014, **10**(8):1757-1766.
